# Integrated Transcriptomic and Bioinformatics Analyses Reveal the Molecular Mechanisms for the Differences in Seed Oil and Starch Content Between *Glycine max* and *Cicer arietinum*

**DOI:** 10.3389/fpls.2021.743680

**Published:** 2021-10-26

**Authors:** Kun Cheng, Yi-Fan Pan, Lü-Meng Liu, Han-Qing Zhang, Yuan-Ming Zhang

**Affiliations:** Crop Information Center, College of Plant Science and Technology, Huazhong Agricultural University, Wuhan, China

**Keywords:** lipid synthesis, starch synthesis, gene regulation network, soybean, chickpea

## Abstract

The seed oil and starch content of soybean are significantly different from that of chickpea. However, there are limited studies on its molecular mechanisms. To address this issue, we conducted integrated transcriptomic and bioinformatics analyses for species-specific genes and acyl-lipid-, starch-, and carbon metabolism-related genes. Among seven expressional patterns of soybean-specific genes, four were highly expressed at the middle- and late oil accumulation stages; these genes significantly enriched fatty acid synthesis and carbon metabolism, and along with common acetyl CoA carboxylase (ACCase) highly expressed at soybean middle seed development stage, common starch-degrading enzyme beta-amylase-5 (BAM5) was highly expressed at soybean early seed development stage and oil synthesis-related genes *ACCase, KAS, KAR, ACP*, and *long-chain acyl-CoA synthetase* (*LACS*) were co-expressed with *WRI1*, which may result in high seed oil content and low seed starch content in soybean. The common ADP-glucose pyrophosphorylase (AGPase) was highly expressed at chickpea middle seed development stage, along with more starch biosynthesis genes co-expressed with four-transcription-factor homologous genes in chickpea than in soybean, and the common WRI1 was not co-expressed with oil synthesis genes in chickpea, which may result in high seed starch content and low seed oil content in chickpea. The above results may be used to improve chickpea seed oil content in two ways. One is to edit *CaWRI1* to co-express with oil synthesis-related genes, which may increase carbon metabolites flowing to oil synthesis, and another is to increase the expression levels of miRNA159 and miRNA319 to inhibit the expression of *MYB33*, which may downregulate starch synthesis-related genes, making more carbon metabolites flow into oil synthesis. Our study will provide a basis for future breeding efforts to increase the oil content of chickpea seeds.

## Introduction

Legumes are an indispensable part of the human diet. They provide one-third of dietary protein and are also an important source of animal feed and edible oil (Zhu et al., 2005). As we know, there are cold- and warm-season legumes. The former, such as chickpea and pea, are rich in starch, while the latter, such as soybean and peanut, are rich in oil and protein (Rathi et al., 2016). Although seed oil and starch content in soybean and chickpea depend on the supply of carbon in plants (Weselake et al., 2009), there are limited studies on the molecular mechanisms for the differences in seed oil and starch content of the two crops.

In recent decades, efforts have been made to dissect the molecular mechanism of seed oil and starch synthesis. In seed oil synthesis, there are four main steps: fatty acid *de novo* synthesis, acyl elongation and editing, triacylglycerol (TAG) assembly, and oil droplet formation. The expression levels of the relevant genes in these steps affect seed oil content, e.g., acetyl CoA carboxylase (ACCase) in rapeseed (Roesler et al., 1997) and potato (Klaus et al., 2004), β-Ketoacyl-[acyl carrier protein] synthase (KASI) in *Arabidopsis*, rice, and soybean (Wu and Xue, 2010; Ding et al., 2015; Dobbels et al., 2017), diacylglycerol acyltransferase (DGAT) in peanut, *Arabidopsis*, and oil palm (Saha et al., 2006; Hernández et al., 2012; Rosli et al., 2018), and oleosins (OLE) in *Arabidopsis* (Shimada and Hara-Nishimura, 2010). Seed starch synthesis begins with the generation of adenosine diphosphate (ADP) glucose *via* the enzyme ADP-glucose pyrophosphorylase (AGPase). AGPase (Tang et al., 2016), soluble starch synthase (SS) (Cuesta-Sejio et al., 2016), granule-bound starch synthases (GBSS) (Denyer et al., 1999), starch branching enzyme (SBE) (Lu et al., 2015), and isoamylase (ISA) (Bustos et al., 2004) are key enzymes in starch synthesis. Transcription factors (TFs) can simultaneously regulate the expression of multiple genes. Key TFs in the regulation of lipid biosynthesis include WRI1 (Ma et al., 2015), LEC1 (Tan et al., 2011), LEC2 (Baud et al., 2007), FUS3 (Wang et al., 2007), and ABI3 (Crowe et al., 2000). TFs in the biosynthesis of starch include SUSH33A2 (Sun et al., 2003), OsbZIP58 (Wang et al., 2013), OsMADS6 and OsMADS29 (Nayar et al., 2013), ABI4 (Hu et al., 2012), and ZmMYB138 and ZmMYB115 (Hu et al., 2021). The above enzymes and TFs may help us to dissect the molecular mechanisms for the differences in seed oil and starch content of soybean and chickpea.

In recent years, some studies have focused on the molecular mechanism of complex trait formation. To dissect the photosynthetic mechanism of C_4_ and C_3_ crops, for example, Wang et al. (2014) compared their genomes and transcriptomes and found that higher expression levels of orthologous genes related to photosynthetic development at the base of rice leaves compared to that in maize, *cis* element (RGCGR; R = A/G) that is only present in promoters of maize, and 118 TFs that contributed to the expression levels of C_4_ photosynthetic cell-type-specific genes, may result in the photosynthesis differences between C_4_ and C_3_ crops. To dissect the molecular mechanism of seed starch content between adzuki bean and soybean, Yang et al. (2015) performed comparative genomic and transcriptome analyses and found no significant variation of starch biosynthesis genes in the two genomes, but the increased expressional levels of these genes at the mature seed stage in adzuki bean and the decreased expressional levels of these genes at the seed filling and mature stages in soybean may result in the difference of seed starch content. To dissect the molecular mechanism of seed oil content between soybean and rapeseed, Zhang et al. (2018) compared genomes, transcriptomes, and miRNA regulation and found that the inhibition of *BnPEPC1* by bna-miR169, high expression levels of rape-specific genes encoding acetyl-coenzyme β-CT, and biotin carboxyl carrier protein-1 (BCCP1) subunits at the seed development stage, along with the expansion of rapeseed lipid storage-related genes and the contraction of lipid degradation-related genes, may result in high seed oil content in rapeseed. However, the molecular mechanisms for the differences of seed oil and starch content in soybean and chickpea are unclear.

To dissect the molecular mechanism for the differences of seed oil and starch content in soybean and chickpea, integrated transcriptomic and bioinformatics analyses for species-specific genes and acyl-lipid-, starch-, and carbon metabolism-related genes, along with miRNA, TF, and co-expression network analysis, were carried out in this study. As a result, it was found that ACCase, BAM5, AGPase, and WRI1 may be related to the above difference. In addition, we discussed how to improve seed oil content in chickpeas. The results provide an explanation for the differences in seed oil and starch content of the two crops.

## Materials and Methods

### Genomic and Proteomic Data

The protein-coding sequences of all the genes in *Arabidopsis*, soybean, peanut, pea, and chickpea were downloaded from https://www.arabidopsis.org/index.jsp (TAIR10), https://www.soybase.org/ (Wm82.a2.v1), https://legumeinfo.org/data/public/Arachis_hypogaea (arahy.Tifrunner.CCJH), https://legumeinfo.org/data/public/Pisum_sativum (Cameor.gnm1. ann1.7SZR), and https://phytozome.jgi.doe.gov/pz/portal.html (Carietinum_492_v1.0), respectively. If there were multiple transcripts for a gene, the longest one was selected.

### Transcriptome Data

Transcriptome datasets in soybean, chickpea, peanut, and pea were downloaded from https://www.ncbi.nlm.nih.gov/geo/query/acc.cgi?acc=GSE42871 (GSE42871; Jones and Vodkin, 2013), https://www.ncbi.nlm.nih.gov/geo/query/acc.cgi?acc=GSE79719 and https://www.ncbi.nlm.nih.gov/geo/query/acc.cgi?acc=GSE79720 (GSE79719 & GSE79720; Garg et al., 2017), https://peanutbase.org/gene_expression/ atlas (Clevenger et al., 2016 and Alves-Carvalho et al., 2015), respectively. The soybean datasets included seven developmental stages: whole seed 4 days after fertilization (DAF) (R2); whole seed 12–14 DAF (R3); whole seed 22–24 DAF (R4); whole seed 5–6 mg in weight (R5); cotyledons 5–6 mg in weight, seed coats 5–6 mg in weight, cotyledons 100–200 mg in weight (R6); cotyledons 400–500 mg in weight (R7); and dry whole seed (R8). The chickpea datasets included S1–S7 seed development stages and leaf. The stages S1–S3 were the embryo development stages (embryogenesis), stages S4 and S5 were early and mid-maturation stages, respectively (grain filling), and stages S6 and S7 were late mature stages (seed desiccation). Based on the definition of soybean vegetative and reproductive growth (Ritchie et al., 1982), whole seed 12–14 DAF (R3), whole seed 22–24 DAF (R4), whole seed 5–6 mg in weight (R5), cotyledons 400–500 mg in weight (R7), and dry whole seed (R8) in soybean were almost consistent with the stages S2, S3, S4, S5, and S7 in chickpea, respectively. These stages were uniformly defined as the stages t1, t2, t3, t4, and t5 in this study, respectively. Thus, the five stages were selected to conduct expressional trend analysis. The peanut datasets included Pattee 5 seed, Pattee 6 seed, Pattee 7 seed, Pattee 8 seed, and Pattee 10 seed. The pea datasets included Stem_BC_LN, Pods_C_LN, seeds_5dai, seeds_5dai_mut, Seeds_12dap, Seeds_12dap_mut, RootSys_A_HN, RootSys_A_LN, Leaf_B_LN, and Flower_B_LN. The gene expression levels [reads per kilobase per million mapped (RPKM) reads] were normalized and quantified by the DESeq package in Bioconductor. The package DESeq provides methods to test for differential expression by estimating variance-mean dependence in count data from high-throughput sequencing assays and test for differential expression based on a model.

Zhang et al. (2018) described that the relative expression level was the ratio of the expression level of each gene to the average expression level of all genes in this species.

### Identification of Common and Specific Genes in Soybean and Chickpea

In this study, all the non-redundant whole-genome protein sequences in *Arabidopsis*, soybean, peanut, pea, and chickpea were clustered by the software package OrthoFinder 2.0 (Emms and Kelly, 2019) to identify orthologous gene families. All the genes of soybean and chickpea were analyzed by MCscan (Tang et al., 2008) to identify homologous collinearity gene fragments.

### Co-expressional Network Analysis for Specific Genes in Soybean and Chickpea

The expressional levels of soybean- and chickpea-specific genes at the above seven different seed development stages and tissues were analyzed by R package WGCNA v1.68 to construct co-expressional networks. When calculating the scale-free topological overlap matrix (TOM), the parameters “unsigned” and “bicor” were used. The optimal soft thresholds in soybean and chickpea were calculated by the function “pickSoftThreshold,” and their thresholds were 10. The minimum module size was set to 30, and the “merge cut height” parameter of the module was set to 0.3 (Langfelder and Horvath, 2008). The KEGG enrichment analysis for the genes in the above co-expressional networks was conducted by KOBAS (http://kobas.cbi.pku.edu.cn/index.php, version 2.0; Xie et al., 2011).

### Cluster Analysis for Expressional Levels of Common Genes in Soybean and Chickpea

The expressional levels of soybean and chickpea common genes at the t1–t5 stages were used to perform cluster analysis, as described in Zhang et al. (2018), using Short Time-series Expression Miner (STEM, http://www.cs.cmu.edu/~jernst/stem/) (Ernst and Bar-Joseph, 2006) with the following parameters: log normalize a time-series vector of gene expression values (*v*_0_, *v*_1_, *v*_2_,…, *v*_*n*_) to [0, log_2_(*v*_1_/*v*_0_), log_2_(*v*_2_/*v*_0_), …, log_2_(*v*_*n*_/*v*_0_)], minimum absolute expression change 2, –*p* 0.05.

### Copy Number Analysis of Gene Families Related to Carbon, Lipid, and Starch Metabolism

One hundred and thirty-five genes related to fatty acid synthesis, TAG synthesis, lipid droplet assembly, and storage were downloaded from ARALIP (http://aralip.plantbiology.msu.edu/; Li-Beisson et al., 2013; Zhang et al., 2018). Thirty-seven starch metabolism-related genes in *Arabidopsis* were mined from Schwarte et al. (2015). Using the method of Troncoso-Ponce et al. (2011), 238 genes related to carbon metabolism were obtained. The copy number difference analysis was carried out for the above 410 genes (Supplementary Table 4). Meanwhile, the 410 genes were compared with the ones in categories three and four of the STEM analysis to mine common genes related to carbon, lipid, and starch metabolism.

### Identification of microRNA, TFs, and Their Target Genes

The microRNAs in soybean and chickpea were downloaded from miRbase (21st edition, http://www.mirbase.org/) and Jain et al. (2014), respectively. The online software psRNATarget (http://plantgrn.noble.org/psRNATarget) (Dai and Zhao, 2011) was used to identify miRNA targets with default parameters except for the Expectation (e), which was set at 3. The TFs were downloaded from PlantTFDB 3.0 (http://planttfdb.cbi.pku.edu.cn/) (Jin et al., 2017). The expressional levels of 88 gene families obtained from copy number analysis and the target TFs of microRNA were used to calculate the Pearson correlation coefficient.

### Gene Family Phylogenetic Tree Construction

The gene families of *WRI1* in soybean, chickpea, *Arabidopsis*, peanut, and pea were used to construct a gene phylogenetic tree using the neighbor-joining (NJ) method, implemented by software MEGA7 (http://www.megasoftware.net/, Kumar et al., 2016); the bootstrap test was repeated 1,000 times, using Poisson distribution model and “pairwise deletion” option. The iTOL (http://itol.embl.de/) was used to visualize and post-beautify the final result tree file.

### Conserved Domain Analysis of Gene and Protein Sequences

The online tool MEME (http://meme-suite.org/tools/meme, v4.11.2) was used to predict the conserved domains of key protein-coding genes (Bailey and Elkan, 1994). The Clustalw program in the MEGA7 software was used to perform a multiple sequence alignment of amino acid sequences of the *WRI1* homologous gene family in *Arabidopsis*, soybean, chickpea, peanut, and pea with default parameters. The GeneDoc software (https://github.com/karlnicholas/GeneDoc, Nicholas et al., 1997) was used to compare and beautify the results.

## Results

### Identification of Common and Specific Genes in Soybean and Chickpea

The collinearity analysis tool MCscan was used to analyze the collinearity of soybean and chickpea genomes. As a result, there were 31,519 and 1,443 repeated gene pairs in the soybean and chickpea genomes, respectively. The latter was far fewer than the former, which may be due to the soybean-specific tetraploid (Wang et al., 2017). In soybean, 472, 305, and 152 blocks had more than 10, 20, and 50 collinear genes, which contained 29,101 (52.8%), 26,689 (48.4%), and 21,172 (39.5%) collinear genes, respectively. In chickpea, 45, 21, and 5 blocks had more than 10, 20, and 50 collinear genes, which contained 1,214 (4.3%), 830 (2.9%), and 380 (1.3%) collinear genes, respectively (Supplementary Table 2).

All the 223,609 protein-coding genes in soybean, chickpea, *Arabidopsis*, peanut, and pea were clustered by OrthoFinder to identify homologous gene families in soybean and chickpea. As a result, these genes were clustered into 26,575 orthologous groups (OGs) (Supplementary Table 1), and each OG represents a gene family. Among these OGs, there were 335 OGs with both soybean single-copy and chickpea multi-copy genes, 8,675 OGs with both soybean multi-copy and chickpea single-copy genes, 3,261 OGs with soybean and chickpea multi-copy genes, and 2,396 OGs with soybean and chickpea single-copy genes, which are regarded as common genes, while 18,657 (33.29%) were defined as soybean-specific paralogous gene clusters without chickpea genes and only 8,010 (28.33%) were chickpea-specific genes (Supplementary Table 3; Zhang et al., 2018).

### Co-expression and KEGG Analyses of Specific Genes in Soybean or Chickpea

**Figure 1:**
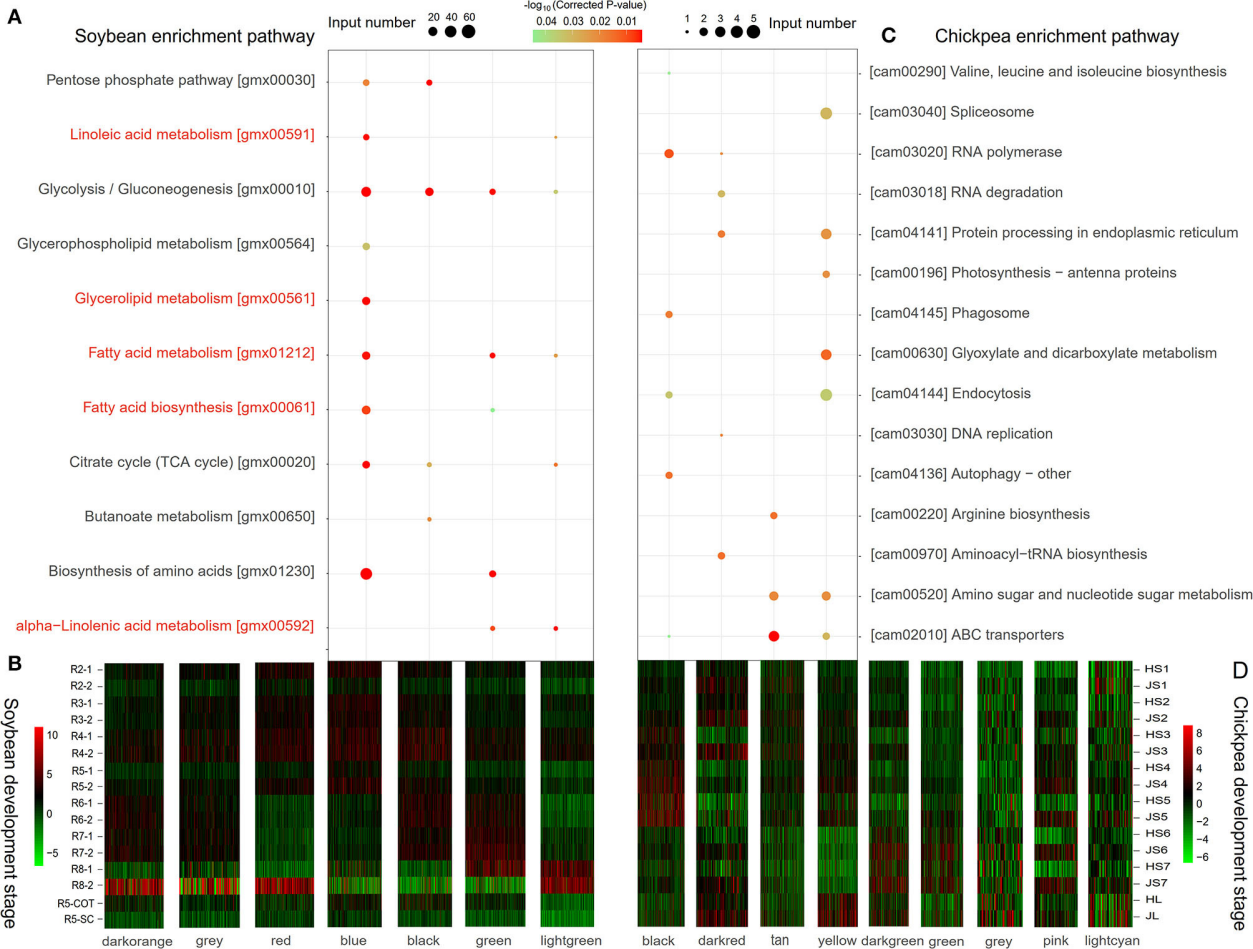
The KEGG pathway enrichment analysis **(A,C)** and expression heatmap **(B,D)** of soybean- **(A,B)** and chickpea-specific **(C,D)** genes. Among seven modules in soybean-specific genes, all the genes in blue, black, green, and light green modules were significantly enriched in oil synthesis-related pathways. The pathways marked with red color were related to lipid metabolism. Among nine modules in chickpea-specific genes, all the genes in black, dark red, tan, and yellow modules were significantly enriched in cell replication-related pathways.

The expressional levels of 18,657 soybean-specific genes at seven different seed development stages and tissues (Jones and Vodkin, 2013) were analyzed by R package WGCNA v1.68 to obtain different co-expression modules. As a result, seven co-expression modules with different expression patterns were identified. The genes in each module were used to conduct KEGG enrichment analysis. Results showed that four modules, blue (3,318 genes), black (900 genes), green (563 genes), and light green (250 genes), significantly enriched multiple lipid metabolism pathways (*P* < 0.05; Figure 1B). The genes in the blue module were highly expressed between R2 and R5 seed development stages, and significantly enriched not only the carbohydrate metabolism pathways of pyruvate metabolism (gmx00620), pentose phosphate pathway (gmx00030), carbon metabolism (gmx01200), and glycolysis (gmx00010), but also the glycerolipid metabolism pathway (gmx00561), glycerophospholipid metabolism (gmx00564), linoleic acid metabolism (gmx00591), and other metabolic pathways. Meanwhile, these genes enriched the photosynthesis-antenna protein (gmx00196) and carbon fixation (gmx00710) metabolic pathways in photosynthetic organisms. Therefore, the blue module is considered to be a co-expression module for oil accumulation during soybean seed storage material accumulation (Figure 1A).

The expressional levels of 8,010 chickpea-specific genes at seven different seed development stages and tissues (Garg et al., 2017) were also analyzed. As a result, nine co-expression modules with different expression patterns were identified. The genes in the black (573 genes), tan (190 genes), yellow (335 genes), and dark red (101 genes) modules were highly expressed at S3–S5 stages, and they enriched cell replication and synthesis-related metabolic pathways. This may be related to the rapid expansion of the number of seed cells (Figures 1C,D).

### Expressional Trends of Common Genes Between Soybean and Chickpea

To obtain the expressional trends of soybean and chickpea common genes, the standardized expression levels of 37,387 soybean and 20,259 chickpea common genes at different seed development stages (Supplementary Figure 1) were used to conduct cluster analysis with the *k*-means algorithm *via* the STEM software. As a result, these common genes were clustered into six clusters. Among these clusters, the expression of all the genes in Cluster3–Cluster5 was upregulated during the t2–t4 stages, while the expression of all the genes in Cluster1 was downregulated during the t2–t4 stages.

All the genes in each cluster were used to conduct KEGG metabolic pathway enrichment analysis. As a result, all the soybean genes in Cluster3 and Cluster4 enriched pentose phosphate pathway (gmx00030), fructose and mannose metabolism (gmx00051), galactose metabolism (gmx00052), fatty acid biosynthesis (gmx00061), fatty acid degradation (gmx00071), starch and sucrose metabolism (gmx00500), pyruvate metabolism (gmx00620), glyoxylic acid and dicarboxylic acid metabolism (gmx00630), carbon fixation in photosynthetic organisms (gmx00710), fatty acid metabolism (gmx01212), and other metabolic pathways (Supplementary Figure 2A). Meanwhile, all the chickpea genes in Cluster4 mainly enriched fatty acid biosynthesis (cam00061), fatty acid metabolism (cam01212), glycolysis/gluconeogenesis (cam00010), citrate cycle (TCA cycle) (cam00020), carbon fixation in photosynthetic organisms (cam00710), glycerophospholipids metabolism (cam00564), starch and sucrose metabolism (cam00500), glyoxylate and dicarboxylate metabolism (cam00630), and other pathways (Supplementary Figure 2B). Clearly, the above enriched metabolic pathways were found to be related to seed lipid and starch synthesis, and the expression of these genes was upregulated during the rapid accumulation stages of these substances, indicating the existence of fatty acids and starch in soybean and chickpea.

### Identification of Candidate Oil- and Starch Biosynthesis-Related Genes in Common Genes

Four hundred and ten carbon-, lipid-, and starch metabolism-related genes in *Arabidopsis thaliana* (Supplementary Table 4) were used as the seed to search for gene families in 26,575 OGs. As a result, 374 common gene families were mined. Among these gene families, six had a >2 copy number ratio of soybean to chickpea, while eight had a copy number ratio of <1. The former includes GRF2 during photosynthesis, CT-α and Acyl carrier protein (ACP) involved in the fatty acid synthesis, and CDS, PECT, and DGAT participating in TAG synthesis, while the latter includes STP7 involved in carbohydrate transport, PKp-β involved in glycolysis, Beta-hydroxyacyl-acyl carrier protein dehydratase (HAD) and β-Ketoacy-acyl carrier protein synthase II (KAS II) participating in the fatty acid synthesis, glycerol-3-phosphate acyltransferase (GPAT9), LPAAT3, and FAD2 participating in TAG synthesis, and Pyrophosphorylase (PPA) involved in starch metabolism (Supplementary Figure 3). Meanwhile, the above 410 genes were traversed and the genes in Cluster3 and Cluster4 were searched to obtain homologous gene families. As a result, 158 soybean genes and 121 chickpea genes were found to be homologous with the above 410 genes.

By merging the above 279 upregulated genes and the above 14 gene families with copy number difference, 184 soybean and 135 chickpea genes in 97 OGs were obtained (Supplementary Table 5), which are candidate oil- and starch biosynthesis-related common genes in soybean and chickpea, and some genes have been confirmed by molecular biological experiments (Table 1).

**Table 1:** The confirmed candidate genes for the differences of soybean and chickpea seed oil and starch content in previous studies.

**Biological process**	**Known genes in** ***Arabidopsis***			**Comparative genomics analysis**		**References**
	***Arabidopsis***	**Gene**	**Functional annotation**	**Homologs in soybean**	**Homologs in chickpea**	
Plastid fatty acid synthesis from pyruvate	*AT5G16390*	*BCCP1*	Biotin Carboxyl Carrier Protein of Heteromeric ACCase	*Glyma.18G243500* *Glyma.09G248900*	*Ca_06111*	Klaus et al., 2004
	*AT5G35360*	*BC*	Biotin Carboxylase of Heteromeric ACCase	*Glyma.08G027600* *Glyma.05G221100*	*Ca_05874*	Roesler et al., 1997
	*AT5G46290*	*KASI*	Ketoacyl-ACP Synthase I	*Glyma.08G084300*	*Ca_05157*	Yang et al., 2016
	*AT1G74960*	*KASII*	Ketoacyl-ACP Synthase II	*Glyma.17G047000*	*Ca_03125* *Ca_19284*	Aslan et al., 2015
	*AT1G62640*	*KASIII*	Ketoacyl-ACP Synthase III	*Glyma.18G211400* *Glyma.09G277400*	*Ca_02927*	Choi et al., 2000
	*AT1G77590*	*LACS*	Long-Chain Acyl-CoA Synthetase	*Glyma.06G112900* *Glyma.13G079900* *Glyma.20G060100*	*Ca_12668 Ca_13132*	Zhao et al., 2010
TAG synthesis	*AT5G60620*	*GPAT9*	Glycerol-3-Phosphate Acyltransferase	*Glyma.09G119200*	*Ca_17353* *Ca_25130*	Singer et al., 2016
	*AT1G51260*	*LPAAT3*	1-Acylglycerol-3-Phosphate Acyltransferase	*Glyma.15G034100*	*Ca_02646* *Ca_05840*	Liu et al., 2015
	*AT2G19450*	*DGAT1*	Acyl-CoA: Diacylglycerol Acyltransferase	*Glyma.13G106100* *Glyma.09G065300* *Glyma.17G053300*	*Ca_03178*	Routaboul et al., 1999
	*AT3G51520*	*DGAT2*	Acyl-CoA: Diacylglycerol Acyltransferase	*Glyma.01G156000*	*Ca_10695*	Gao et al., 2021
	*AT5G13640*	*PDAT1*	Phospholipid: Diacylglycerol Acyltransferase	*Glyma.07G036400* *Glyma.13G108100* *Glyma.16G005800*	*Ca_03160* *Ca_10036*	Fan et al., 2013
	*AT1G12640*	*LPCAT*	1-acylglycerol-3-phosphocholine Acyltransferase	*Glyma.17G131500*	*Ca_08736*	Bates et al., 2012
Starch metabolism	*AT5G48300*	*ApS1*	ADP-glucose pyrophosphorylase	*Glyma.02G304500*	*Ca_07632* *Ca_09767*	Li et al., 2011
	*AT5G19220*	*ApL1*	Glucose-1-phosphate adenylyltransferase	*Glyma.07G258500*	*Ca_03357*	Tuncel et al., 2014
	*AT5G24300*	*SSI*	Starch synthase	*Glyma.04G235200*	*Ca_03985*	Nakamura, 2002
	*AT3G01180*	*SSII*	Starch synthase	*Glyma.13G062700* *Glyma.19G022900*	*Ca_10512*	Craig et al., 1998
	*AT1G11720*	*SSIV*	Starch synthase	*Glyma.05G127800*	*Ca_05169*	Szydlowski et al., 2009
	*AT4G18240*	*SSIII*	Starch synthase	*Glyma.13G204700*	*Ca_00636*	Gao et al., 1998
	*AT1G32900*	*GBSS*	Granule-bound starch synthase	*Glyma.20G218100* *Glyma.07G049900*	*Ca_22418*	Ceballos et al., 2007
	*AT2G39930*	*ISA1*	Isoamylase1	*Glyma.08G028400*	*Ca_05882*	Delatte et al., 2005
	*AT3G20440*	*BE2*	Isoamylase3	*Glyma.18G092600*	*Ca_20526*	Dumez et al., 2006
	*AT4G15210*	*BAM5*	Beta-amylase	*Glyma.12G102900* *Glyma.06G301500*	*Ca_22584*	Andriotis et al., 2010

### Expression Trends of Candidate Oil and Starch Synthesis Genes

To compare the expressional profile difference of each gene in the above 97 OGs at t1–t5 stages in soybean and chickpea, the relative expression level was used in this study (Supplementary Table 6). In soybean, most starch synthesis-related genes were highly expressed at the early seed development stage, while most oil synthesis-related genes were highly expressed in the middle seed development stage, which is also the rapid oil accumulation stage. In chickpea, most starch synthesis-related genes were highly expressed at the middle seed development stage.

**Figure 2:**
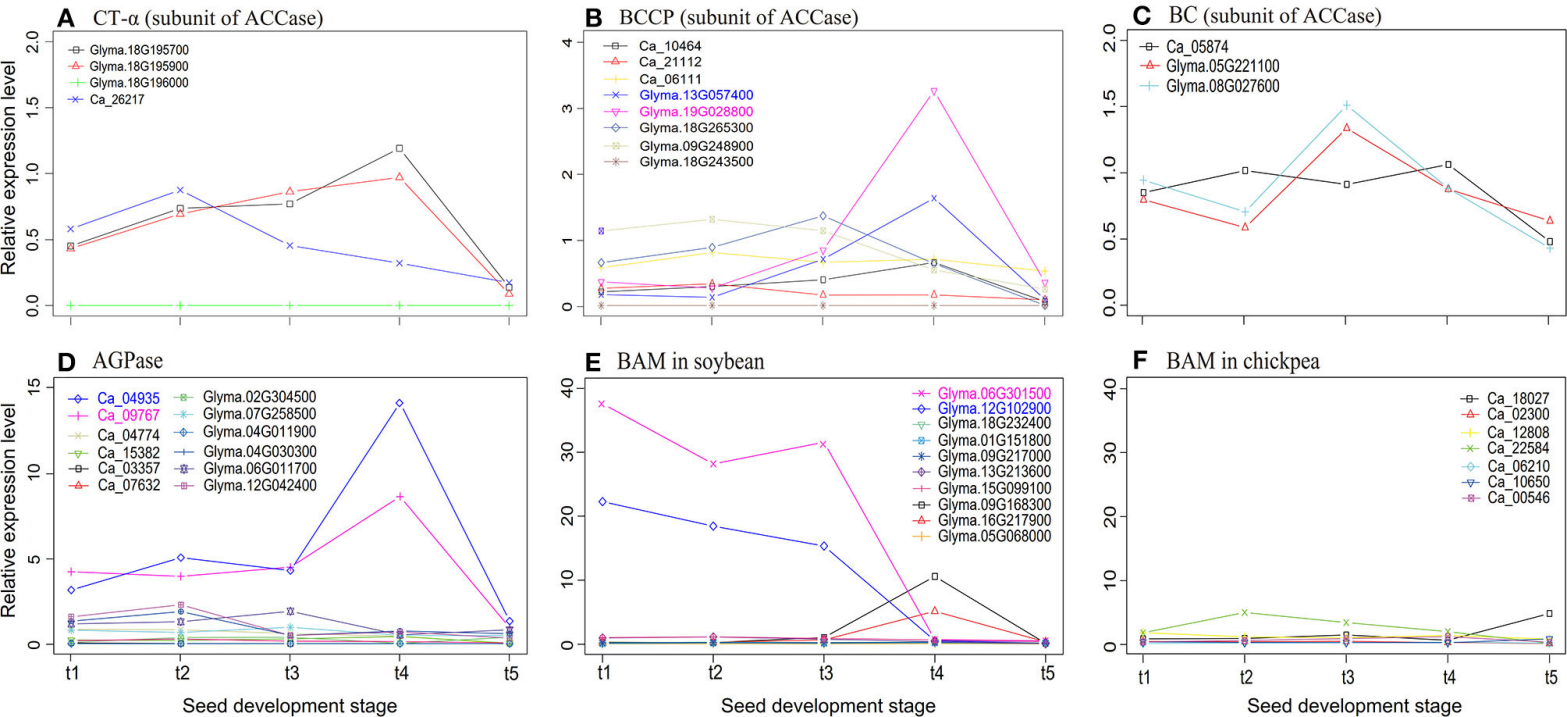
The relative expression levels of three subunits *CT-*α **(A)**, *BCCP* **(B)**, and *BC* **(C)** of ACCase, AGPase **(D)**, and *BAM* **(E,F)**, which are soybean and chickpea common genes, at five seed development stages. The relative expression level was defined by Zhang et al. (2018) as the ratio of the expression level of each gene to the average expression level of all genes in this species. AGPase, ADP-glucose pyrophosphorylase; BC, biotin carboxylase of heteromeric ACCase; BCCP, biotin carboxyl carrier protein; MAB-5, beta-amylase-5.

Based on the studies in Klaus et al. (2004), Tang et al. (2016), and Andriotis et al. (2010), common genes *ACCase, AGPase*, and *BAM5* were important genes in seed oil and starch metabolism. Thus, we compared the expressional profile differences of these genes in soybean and chickpea. First, the relative expression levels of genes encoding subunits α-CT, BCCP, and BC of ACCase at rapid oil accumulation stages were higher in soybean than in the chickpea (Figures 2A–C). Because ACCase catalyzes the first step of the fatty acid synthesis, which is the formation of malonyl-CoA from acetyl-CoA (Klaus et al., 2004), ACCase may make more carbon metabolites flow into the fatty acid synthesis in soybean seeds. Then, the relative expression levels of genes encoding subunits ADP-glucose pyrophosphorylase (APS) and glucose-1-phosphate adenylyltransferase (APL) of AGPase at the t4 stage were much higher in chickpea than in soybean (Figure 2D).

Because AGPase is the first key regulatory enzyme and rate-limiting enzyme in starch synthesis (Tang et al., 2016), AGPase may play a significant role in chickpea starch synthesis. Finally, the relative expression level of BAM5 at the early seed development stage was much higher in soybean than in chickpea (Figures 2E,F). Because BAM5 catalyzes the production of maltose from linear glucans (Andriotis et al., 2010), it may play a major role in plant starch hydrolysis. This may result in low starch content in soybean seeds. Therefore, we infer that *ACCase, AGPase*, and *BAM5* were more likely to be candidate key genes that affect the differences of seed oil and starch content in soybean and chickpea.

**Figure 3:**
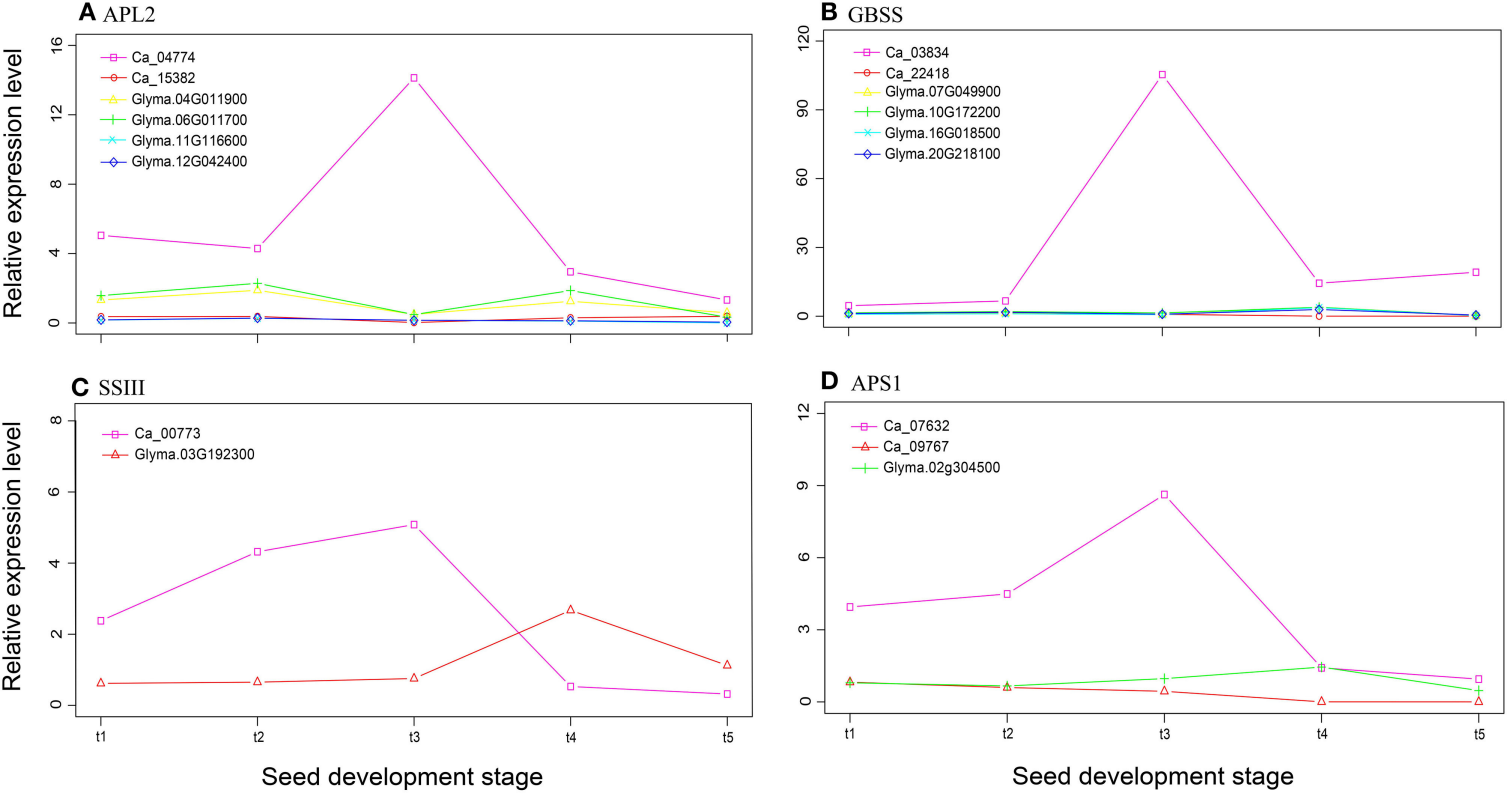
The relative expression levels of four starch synthesis genes *APL2* **(A)**, *GBSS* **(B)**, *SSIII* **(C)**, and *APS1* **(D)**, which are common genes in soybean and chickpea, at five seed development stages.

Using the starch biosynthesis genes in rice (17 genes; Ohdan et al., 2005; Tian et al., 2009) and *Arabidopsis* (13 genes; Schwarte et al., 2015) as bait, we performed an ortholog search in the soybean and chickpea genomes. In total, fewer starch biosynthesis genes were found in chickpea than in soybean (33 vs. 45; Supplementary Table 7), but there was no significant difference between these two genomes in the exact Fisher test (*P* = 1.00). The relative expression levels of these starch biosynthesis genes at seven seed development stages were used to compare their differences. We found that the average relative expression level in chickpea was significantly higher than that of soybean, e.g., Ca_03834, Ca_04774, Ca_07632, and Ca_00773 have a higher expressional level at stage t3 (Figure 3), although more starch biosynthesis genes were detected in the soybean genome (Supplementary Table 7). The above results are similar to those in Yang et al. (2015).

### Comparison of Regulatory Networks of miRNAs, TFs, and Their Target Genes in Soybean and Chickpea

#### miRNAs, TFs, and Their Target Genes

**Figure 4:**
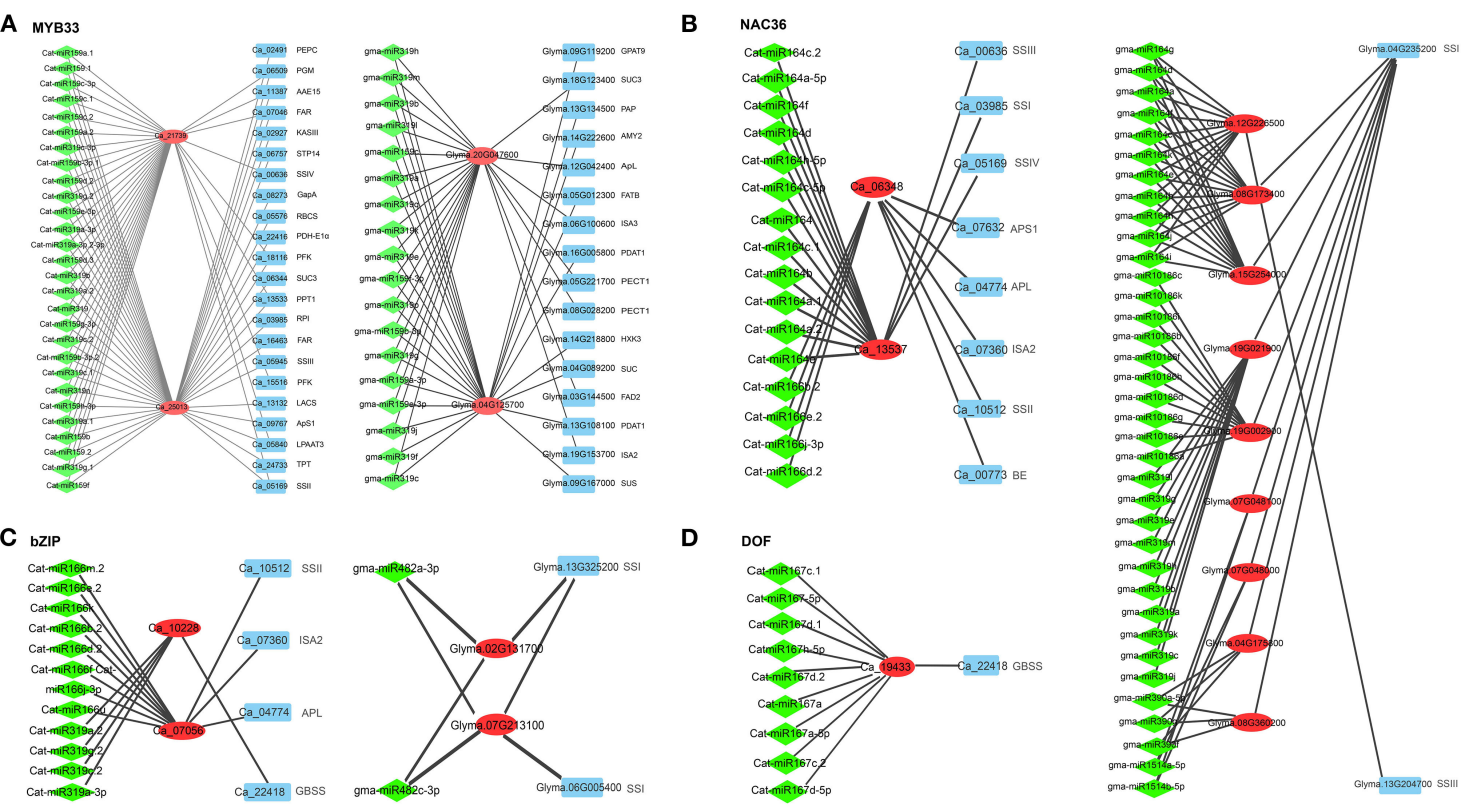
The regulation networks of soybean and chickpea genes (red) homologous to four transcription factors AtMYB33 in *Arabidopsis* **(A)**, NAC36 **(B)**, bZIP **(C)**, and DOF **(D)**. Homologous genes and miRNAs are, respectively, marked by red and cyan colors, while lipid- and starch metabolism-related genes are marked by blue color.

The target genes of 639 soybean and 440 chickpea mature miRNAs were predicted using the online software psRNATarget. As a result, the target genes in 2,303 soybean and 939 chickpea miRNA-target gene pairs were TFs, while the target genes in 90 soybean and 30 chickpea miRNA-target gene pairs were oil- or starch synthesis-related genes. In detail, in soybean gma-miR2111targeted *SSIII* (*Glyma.13G204700*), gma-miR5784- and gma-miR4347 targeted *APS1* (*Glyma.02G304500*), and gma-miR10413 targeted *PGM2* (*Glyma.20G018000*); gma-miR530, gma-miR160, and gma-miR319 were predicted to regulate the expression of the *ABI4, ARF*, and *MYB* gene families, respectively, while gma-miR156 and gma-miR159 were predicted to regulate the expression of the *SBP* gene family (Supplementary Table 8). In chickpea, Cat-miR156 and Cat-miR157 inhibited the expression of *phospholipid: diacylglycerol acyltransferase-2* (*PDAT2*; *Ca_24053*), while Cat-miR171 and Cat-miR5565 inhibited the expression of *FATB* (*Ca_06618*). Cat-miR166 and Cat-miR319 were predicted to regulate the expression of bZIP in chickpea, while gma-miR482 was predicted to regulate the expression of bZIP in soybean. Cat-miR164 and gma-miR164 were predicted to regulate the expression of NAC36 in chickpea and soybean, respectively. Cat-miR167 was predicted to regulate the expression of DOF in chickpea, while gma-miR172 was predicted to regulate the expression of DOF in soybean (Supplementary Table 9; Figure 4). PDAT transfers acyl moiety from PC to DAG to form TAG, which plays an important role in TAG synthesis. Cat-miR160, Cat-miR5674, and Cat-miR156 were predicted to regulate the expression of *ARF, GRAS*, and *SBP* gene families, respectively, while Cat-miR156, Cat-miR159, and Cat-miR319 were predicted to regulate the expression of the *MYB* gene family (Supplementary Table 8).

#### The Regulatory Networks of miRNAs, TFs, and Their Target Genes

As described by Hu et al. (2021), genes involved in a biological network are regulated by the same TF and co-expressed with each other. In the above 2,303 soybean and 939 chickpea miRNA-target gene pairs, there were 457 soybean TFs and 113 chickpea TFs. These TFs were merged with 11 oil synthesis-related TFs and 6 starch synthesis-related homologous gene families in soybean and chickpea. After removing unexpressed TFs, 282 soybean TFs and 93 chickpea TFs were identified. Soybean transcriptomic datasets of 282 TFs and 184 genes at seven different seed development stages and tissues (Jones and Vodkin, 2013) were used to calculate their correlation coefficients (*r*), while chickpea transcriptomic datasets of 93 TFs and 135 genes at seven different seed development stages and tissues (Garg et al., 2017) were used to calculate their correlation coefficients. With the thresholds of |*r*| ≥ 0.8 and *P* ≤ 0.05, a total of 1,905 soybean TF-gene pairs and 590 chickpea TF-gene pairs were found to be significant.

In the regulatory network, miR156, miR159, miR160, and miR319 families in chickpea and soybean commonly regulated MYB and SBP. We found that two chickpea genes and two soybean genes, homologous to AtMYB33, had different co-expressed genes, e.g., *Ca_21739* was co-expressed with *PFK5, FBA, LPAAT3, SSIV, SSIII*, and *AMY2*, and *Ca_25013* was co-expressed with *STP7, PFK5, FBA, GapA, TPT, PDH-E1*α, *KASIII, LPAAT3, LPEAT2, LACS, APS1, SSI*, and *SSIV*, while *Glyma.04G125700* was co-expressed with *AMY2, SUC, SUS, GPAT9, FATB*, and *PDAT1*, and *Glyma.20G047600* was co-expressed with *SUC3, FAD2, PECT1, APL, ISA2*, and *ISA3* (Figure 4A). The four genes were all regulated by miRNA159 and miRNA319. It is speculated that miRNA159 and miRNA319 may regulate the expression of oil- and starch synthesis-related genes *via* regulating MYB gene families in crops. Two chickpea genes, *Ca_10228* and *Ca_07056*, homologous to bZIP, had more co-expressed starch biosynthesis genes than two soybean genes, *Glyma.02G131700* and *Glyma.07G213100*, which are homologous to bZIP; in detail, bZIP was co-expressed with starch biosynthesis genes *APL, ISA2, SSII*, and *GBSS* in chickpea but only with starch biosynthesis gene *SSI* in soybean (Dong et al., 2019; Figure 4C). Two chickpea genes *Ca_06348* and *Ca_13537*, homologous to NAC36, had more co-expressed starch biosynthesis genes than three soybean genes, *Glyma.08G173400, Glyma.12G226500*, and *Glyma.15G254000*, which are homologous to NAC36; in detail, NAC36 was co-expressed with starch biosynthesis genes *APL, APS1, BE, ISA2, SSI, SSII, SSIII*, and *SSIV* in chickpea but only with starch biosynthesis genes *SSI* and *SSIII* in soybean (Zhang et al., 2014; Figure 4B). DOF was co-expressed with one starch biosynthesis gene *Ca_22418* (GBSS) in chickpea but with no starch biosynthesis genes in soybean (Qi et al., 2017; Supplementary Table 9; Figure 4D). It is speculated that miRNA164, miRNA166, miRNA167, miRNA 319, and miRNA482 may regulate the expression of starch synthesis-related genes *via* regulating bZIP, NAC36, and DOF gene families in chickpea and/or soybean. This is a possible reason that seed starch content is higher in chickpea than in soybean.

*GmWRI1* (*Glyma.08G227700* and *Glyma.15G221600*) were co-expressed with lipid synthesis genes *a-CT, BC, BCCP2, KASII, KAR, LACS, FATA, PKp-*α, *PKp-*β, *ACP*, and 1-acylglycerol-3-phosphocholine acyltransferase (*LPCAT)* in soybean, while no lipid synthesis genes in chickpea were found to be co-expressed with *CaWRI1* (*Ca_15711*). Surprisingly, no miRNA was found to regulate *WRI1*. The fact that no lipid synthesis genes co-expressed with WRI1 may be an important reason for low seed oil content in chickpea. Thus, we conducted further analysis for the *WRI1* gene family.

### Phylogenetic Analysis of *WRI1* Gene Family

#### Construction of Phylogenetic Tree of WRI1 Gene Family

**Figure 5:**
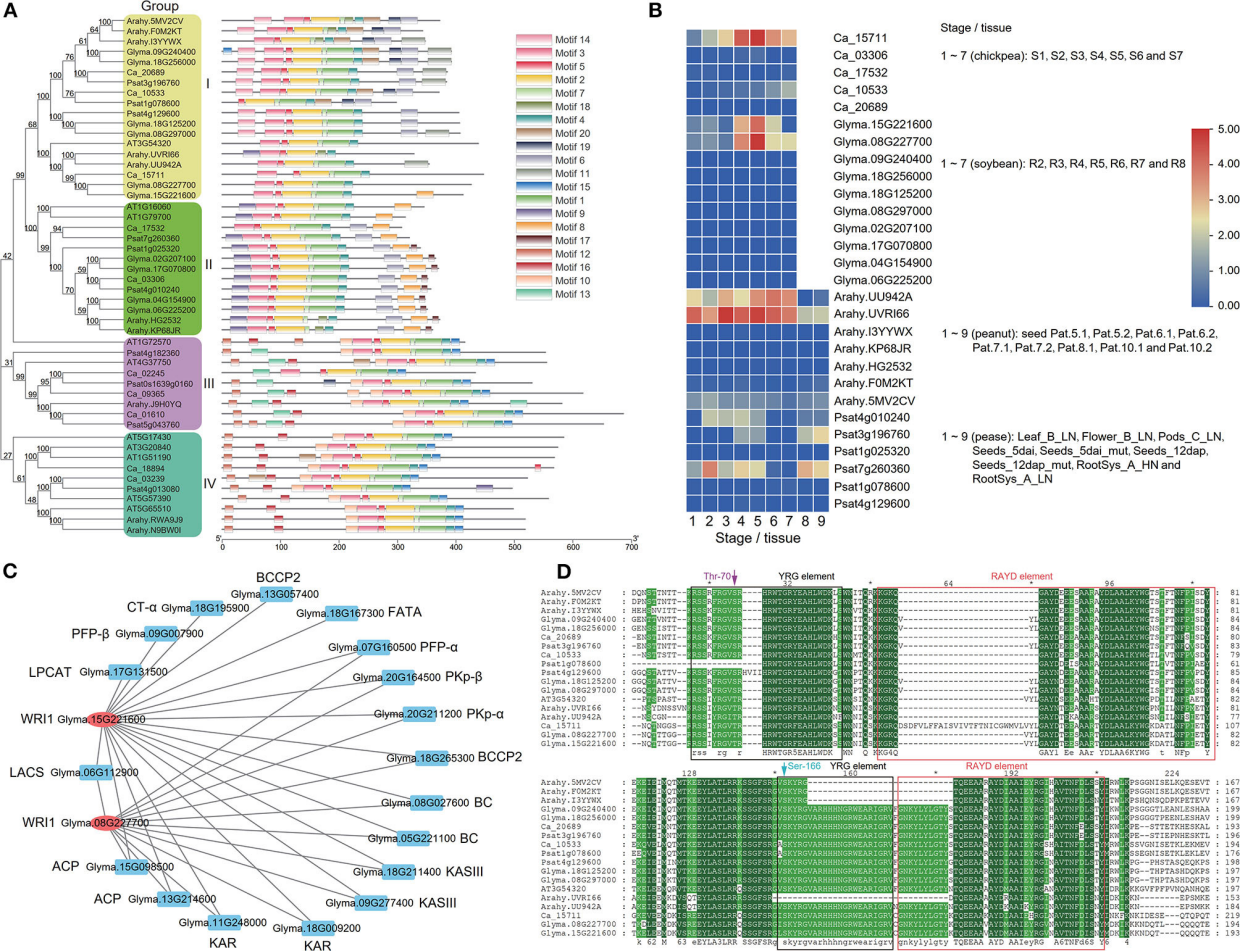
Phylogenetic **(A)**, expression **(B)**, co-expression network **(C)**, and protein sequence **(D)** analyses of the *WRI1* gene family in *Arabidopsis* **(A,D)**, soybean **(A–D)**, chickpea, peanut, and pea **(A,B,D)**. **(C)** The genes with blue color are oil synthesis -related genes, while those with red color are *WRI1* genes. **(D)** There are 24 extra amino acids before the VYL motif in the RAYD element in the first AP2 domain of Ca_15711.

As a member of the AP2 subfamily of the AP2/EREBP TF family, WRI1 plays an important role in the regulation of seed oil synthesis (Tang et al., 2019). To investigate the evolution of the *WRI1* gene family, all the WRI1 homologs in soybean, chickpea, *Arabidopsis*, peanut, and pea were searched by BLASTP using the Arabidopsis WRI1 amino-acid sequence as the query sequence. As a result, 50 sequences with E <1e-10 were downloaded for these species and were used to construct a phylogenetic tree using a NJ method *via* MEGA7. In the evolutionary tree, there were four *WRI* sub-families, which are named Groups I–IV. Among these subfamilies, AtWRI1 was in Group I, while AtWRI4 was in Group II (Figure 5A). To identify their conserved motifs, all the protein sequences were analyzed by the software MEME. The results are shown in Figure 5A. Although the sequence motifs were very similar in Groups I and II, some small differences existed, e.g., *Psat1g078600* in Group I lacked some front part sequences, and *Ca_15711* had a large gap between the first and second conserved motifs. Meanwhile, different sequence motifs in Groups III and IV were observed.

#### Expressional Patterns of WRI1 Subfamilies

To understand the functions of all the WRI genes in Groups I and II in soybean, chickpea, peanut, and pea, we compared the expressional patterns of all the WRI genes at different tissues or seed developmental stages. In Group I, *Ca_15711, Glyma.15G221600, Glyma.08G227700*, and *Arahy.UU942A* had low expression levels at the early seed development stage and high expression levels at lipid synthesis stages (Figure 5B), while *Psat4g010240* and *Psat4g010240* expressed at the early seed development stage and did not express at later stages. In addition, only Ca_10533 had low expression levels at late seed development stages, while no other soybean and chickpea WRI genes in Group I were expressed. In Group II, most WRI genes in soybean, peanut, and chickpea had low or no expression levels, while only *Psat7g260360* in pea was expressed at the early seed development stage. The results are consistent with those of Lee et al. (2009), in which the mRNAs of WRI3 and WRI4 were almost undetectable.

#### Correlation Analysis of WRI1 Genes With Lipid Synthesis Genes

To compare the correlation of *WRI1* genes in Groups I and II with lipid synthesis genes in warm- (peanut and soybean) and cool-season (chickpea and pea) legumes, we calculated correlation coefficients of *WRI1* genes in peanut and pea and lipid synthesis genes. Using the thresholds of |r| ≥ 0.8 and *P* ≤ 0.05, we identified a total of 64 TF-gene pairs. Among these pairs, there were 31, 31, 2, and 0 TF-gene pairs in peanut, soybean, pea, and chickpea, respectively, which were all in Group I rather than in Group II (Table 2; Figure 5C).

**Table 2:** Co-expressional gene pairs of *WRI1* with oil synthesis genes in peanut, soybean, and pea.

**Species**	**Transcription factor**	**Co-expressional gene**	**Gene annotation**	**r**	**P-value**	**Species**	**Transcription factor**	**Co-expressional gene**	**Gene annotation**	**r**	***P*-value**
*Arachis hypogea*	*Arahy.UU942A*	*Arahy.AG8095*	*BCCP2*	0.9576	4.97E-05	*Glycine max*	*Glyma.08G227700*	*Glyma.20G164500*	*PKp-β*	0.8896	3.98E-06
	*Arahy.UU942A*	*Arahy.7G97IV*	*SAD*	0.9546	6.28E-05		*Glyma.08G227700*	*Glyma.20G211200*	*PKp-α*	0.9200	4.55E-07
	*Arahy.UU942A*	*Arahy.QR9F0J*	*FATA*	0.8895	1.32E-03		*Glyma.08G227700*	*Glyma.18G265300*	*BCCP2*	0.9125	8.36E-07
	*Arahy.UU942A*	*Arahy.YI95N7*	*FATA*	0.8436	4.26E-03		*Glyma.08G227700*	*Glyma.08G027600*	*BC*	0.9215	3.99E-07
	*Arahy.UU942A*	*Arahy.0G9KRE*	*GPAT9*	0.8946	1.13E-03		*Glyma.08G227700*	*Glyma.05G221100*	*BC*	0.9347	1.15E-07
	*Arahy.UVRI66*	*Arahy.45ZUWH*	*CT-α*	0.8506	3.66E-03		*Glyma.08G227700*	*Glyma.18G211400*	*KASIII*	0.9013	1.88E-06
	*Arahy.UVRI66*	*Arahy.KBA53Y*	*BCCP2*	0.9054	7.80E-04		*Glyma.08G227700*	*Glyma.09G277400*	*KASIII*	0.8776	7.93E-06
	*Arahy.UVRI66*	*Arahy.1S754L*	*BC*	0.8433	4.28E-03		*Glyma.08G227700*	*Glyma.18G009200*	*KAR*	0.8806	6.74E-06
	*Arahy.UVRI66*	*Arahy.H4HC75*	*BC*	0.8813	1.69E-03		*Glyma.08G227700*	*Glyma.11G248000*	*KAR*	0.8677	1.33E-05
	*Arahy.UVRI66*	*Arahy.GCU9ZU*	*KASI*	0.8432	4.30E-03		*Glyma.08G227700*	*Glyma.13G214600*	*ACP*	0.8525	2.73E-05
	*Arahy.UVRI66*	*Arahy.X916ZF*	*KASI*	0.8408	4.52E-03		*Glyma.08G227700*	*Glyma.15G098500*	*ACP*	0.8290	7.21E-05
	*Arahy.UVRI66*	*Arahy.39SMC6*	*KASII*	0.8931	1.18E-03		*Glyma.08G227700*	*Glyma.06G112900*	*LACS*	0.9678	8.81E-10
	*Arahy.UVRI66*	*Arahy.F6FJSK*	*KASII*	0.8359	5.00E-03		*Glyma.15G221600*	*Glyma.09G007900*	*PFP-β*	0.8323	6.34E-05
	*Arahy.UVRI66*	*Arahy.40PBNN*	*KAR*	0.8709	2.24E-03		*Glyma.15G221600*	*Glyma.07G160500*	*PFP-α*	0.8730	1.02E-05
	*Arahy.UVRI66*	*Arahy.ICU8XT*	*KAR*	0.8390	4.69E-03		*Glyma.15G221600*	*Glyma.20G164500*	*PKp-β*	0.8375	5.17E-05
	*Arahy.UVRI66*	*Arahy.JC4ZCG*	*HAD*	0.9485	9.73E-05		*Glyma.15G221600*	*Glyma.20G211200*	*PKp-α*	0.8923	3.38E-06
	*Arahy.UVRI66*	*Arahy.KP3Y1K*	*HAD*	0.9457	1.17E-04		*Glyma.15G221600*	*Glyma.18G195900*	*CT-α*	0.8028	1.81E-04
	*Arahy.UVRI66*	*Arahy.EA8RPB*	*ACP*	0.9227	3.92E-04		*Glyma.15G221600*	*Glyma.13G057400*	*BCCP2*	0.8434	4.04E-05
	*Arahy.UVRI66*	*Arahy.FL5QND*	*ACP*	0.8600	2.94E-03		*Glyma.15G221600*	*Glyma.18G265300*	*BCCP2*	0.9521	1.36E-08
	*Arahy.UVRI66*	*Arahy.Z9XA8H*	*ACP*	0.9527	7.22E-05		*Glyma.15G221600*	*Glyma.08G027600*	*BC*	0.9386	7.52E-08
	*Arahy.UVRI66*	*Arahy.YI95N7*	*FATA*	0.8744	2.04E-03		*Glyma.15G221600*	*Glyma.05G221100*	*BC*	0.9523	1.33E-08
	*Arahy.UVRI66*	*Arahy.HDYS9D*	*LACS*	0.9433	1.35E-04		*Glyma.15G221600*	*Glyma.18G211400*	*KASIII*	0.8761	8.63E-06
	*Arahy.UVRI66*	*Arahy.L14VXE*	*ACC2*	0.8115	7.92E-03		*Glyma.15G221600*	*Glyma.09G277400*	*KASIII*	0.8922	3.39E-06
	*Arahy.UVRI66*	*Arahy.0G9KRE*	*GPAT9*	0.8691	2.35E-03		*Glyma.15G221600*	*Glyma.18G009200*	*KAR*	0.9060	1.35E-06
	*Arahy.UVRI66*	*Arahy.F7JGYB*	*DGAT3*	0.8782	1.84E-03		*Glyma.15G221600*	*Glyma.11G248000*	*KAR*	0.8948	2.88E-06
	*Arahy.UVRI66*	*Arahy.3LN7S1*	*LACS*	0.8336	5.23E-03		*Glyma.15G221600*	*Glyma.13G214600*	*ACP*	0.9018	1.82E-06
	*Arahy.5MV2CV*	*Arahy.M024AA*	*HAD*	0.8437	4.25E-03		*Glyma.15G221600*	*Glyma.15G098500*	*ACP*	0.8711	1.12E-05
	*Arahy.5MV2CV*	*Arahy.Q99U9Q*	*HAD*	0.8316	5.45E-03		*Glyma.15G221600*	*Glyma.18G167300*	*FATA*	0.8245	8.52E-05
	*Arahy.5MV2CV*	*Arahy.I1811Q*	*LACS*	0.8139	7.59E-03		*Glyma.15G221600*	*Glyma.06G112900*	*LACS*	0.9830	1.07E-11
	*Arahy.5MV2CV*	*Arahy.F78IM4*	*PDAT1*	0.8067	8.61E-03		*Glyma.15G221600*	*Glyma.17G131500*	*LPCAT*	0.8269	7.79E-05
	*Arahy.5MV2CV*	*Arahy.WLZ7Z3*	*FAD3*	0.8433	4.29E-03	*Pisum sativum*	*Psat3g196760*	*Psat7g144640*	*KAR*	0.9660	2.53E-04
*Glycine max*	*Glyma.08G227700*	*Glyma.07G160500*	*PFP-α*	0.8140	1.25E-04		*Psat3g196760*	*Psat7g130880*	*PDAT*	0.9916	2.04E-02

#### Protein Sequence Analysis of All the WRI1 Genes

To understand the structural variation of all the WRI1 genes, we performed multiple alignments for the protein sequence of all the WRI1 genes in Group I. The results are shown in Figure 5D. As described by Okamuro et al. (1997), WRI1 has two AP2/EREB domains, and in each domain, YRG and RAYD elements form the AP2 DNA-binding domain. We found that all the WRI1 genes, except Arahy.UVRI66 and Psat1g078600 included two YRG elements and two RAYD elements. The second YRG element was more conserved than the first one. The tyrosine (Y) in the first YRG motif is conserved in AT3G54320, Arahy.UVRI66, Arahy.UU942A, Ca_15711, Glyma.08G227700, and Glyma.5G221600, while the alanine (A) residue in the first RAYD motif and the arginine (R) residue in the second RAYD motif are not conserved. In the first AP2 domain of Psat1g078600, some sequences in the YRG element were lacking, while in the first AP2 domain of Ca_15711, 24 extra amino acids before the VYL motif in the RAYD element were observed. The above sequence variation may affect the functions of the WRI1 gene in different species.

## Discussion

### Molecular Mechanism of High Oil and Low Starch Content in Soybean Seed

As described above, the soybean-specific genes in four of seven modules were highly expressed at the middle and late oil accumulation stages and significantly enriched in fatty acid, glycerolipid, and linoleic acid metabolisms. Among these genes, main lipid synthesis-related genes include *Glyma.19G147400* (Lakhssassi et al., 2017), *Glyma.07G207200* (Deng et al., 2020), *Glyma.12G146700* (Carther et al., 2019), and *Glyma.06G058500* (Yang et al., 2021), which have been confirmed by molecular biology experiments. In detail, *Glyma.19G147400* is responsible for the conversion of oleic acid to linoleic acid, *Glyma.07G207200* catalyzes the synthesis of monounsaturated oleic acid or palmitoleic acid in plastids, *Glyma.12G146700* transforms the diacylglycerol into phosphatidic acid, and *Glyma.06G058500* is involved in long-chain fatty acids synthesis. Meanwhile, around stable loci for soybean seed oil content in Yao et al. (2020), *Glyma.05G226800, Glyma.05G227400, Glyma.08G102100, Glyma.10G063600, Glyma.11G148900, Glyma.11G190600, Glyma.13G112700, Glyma.13G148100, Glyma.14G150100, Glyma.15G051100*, and *Glyma.20G088000* were found to be soybean-specific genes in the above four modules. Thus, the above soybean-specific and highly expressed genes may result in high seed oil content.

Three soybean copies (*Glyma.13G057400, Glyma.18G265300*, and *Glyma.19G028800*) of common gene *BCCP2* in soybean and chickpea were highly expressed during t3–t4 stages; in particular, *Glyma.19G028800* was rapidly upregulated and reached a peak during t3–t4 stages. As described in Konishi and Sasaki (1994), BCCP2 is an important subunit of ACCase. Overexpression of ACCase can significantly increase fatty acid content in crop seed (Roesler et al., 1997; Klaus et al., 2004), while antisense expression of BCCP2 transcript in developing *Arabidopsis* seeds resulted in an average 38% reduction in BCCP2 protein, further leading to an average 9% reduction in fatty acid content in mature seeds (Thelen and Ohlrogge, 2002). It should be noted that *Glyma.13G057400* and *Glyma.19G028800* (BCCP2) in soybean had significantly higher expression than *Ca_21112* and *Ca_10464* (BCCP2) in chickpea. Thus, the high expression genes *Glyma.13G057400* and *Glyma.19G02880* in soybean may result in its high seed oil content.

In this study, *WRI1* was found to be co-expressed with oil synthesis-related genes *ACCase, KAS, KAR, ACP*, and *LACS* in soybean and peanut. This may result in high seed oil content in soybean. The evidence is as follows. Some target genes of AtWRI1 are involved in *Arabidopsis* glycolysis and fatty acid synthesis, while their mRNAs are accumulated in a synergistic manner and regulated by WRI1 (Baud et al., 2009). Twenty-eight transcripts, such as *KASIII, KAR*, and *LACS*, were found to have increased expression levels in the transgenic plants of *GmWRI1*, which can specifically bind the 500 bp upstream sequences of ATG codon of the above genes and effectively activate their transcription (Chen et al., 2018).

As described in Scheidig et al. (2002), BAM degrades starch and amylopectin to form maltose. In the study of Andriotis et al. (2010), seed starch content was 1.5–2.5 times higher in *Arabidopsis thaliana bam4* and *bam1234* mutants than in wild-type embryos, and the starch still existed in mature embryos, while seed starch content in *amy3, isa3*, and *phs1* mutants did not increase, indicating the necessity of BAM in embryo starch degradation. In this study, two *BAM5* copies, *Glyma.06G301500* and *Glyma.12G102900*, in soybean had more than 6 times expression levels at the early seed development stage than one chickpea *BAM5* gene *Ca_22584*. This indicates that seed starch, produced at the early seed development stage, maybe degraded by *Glyma.06G301500* and *Glyma.12G102900*. This results in low seed starch content in soybean. NAC36-, bZIP-, and DOF-homologous genes in chickpea have more co-expressed starch biosynthesis genes than those in soybean. This may result in higher seed starch content in chickpea than in soybean.

**Figure 6:**
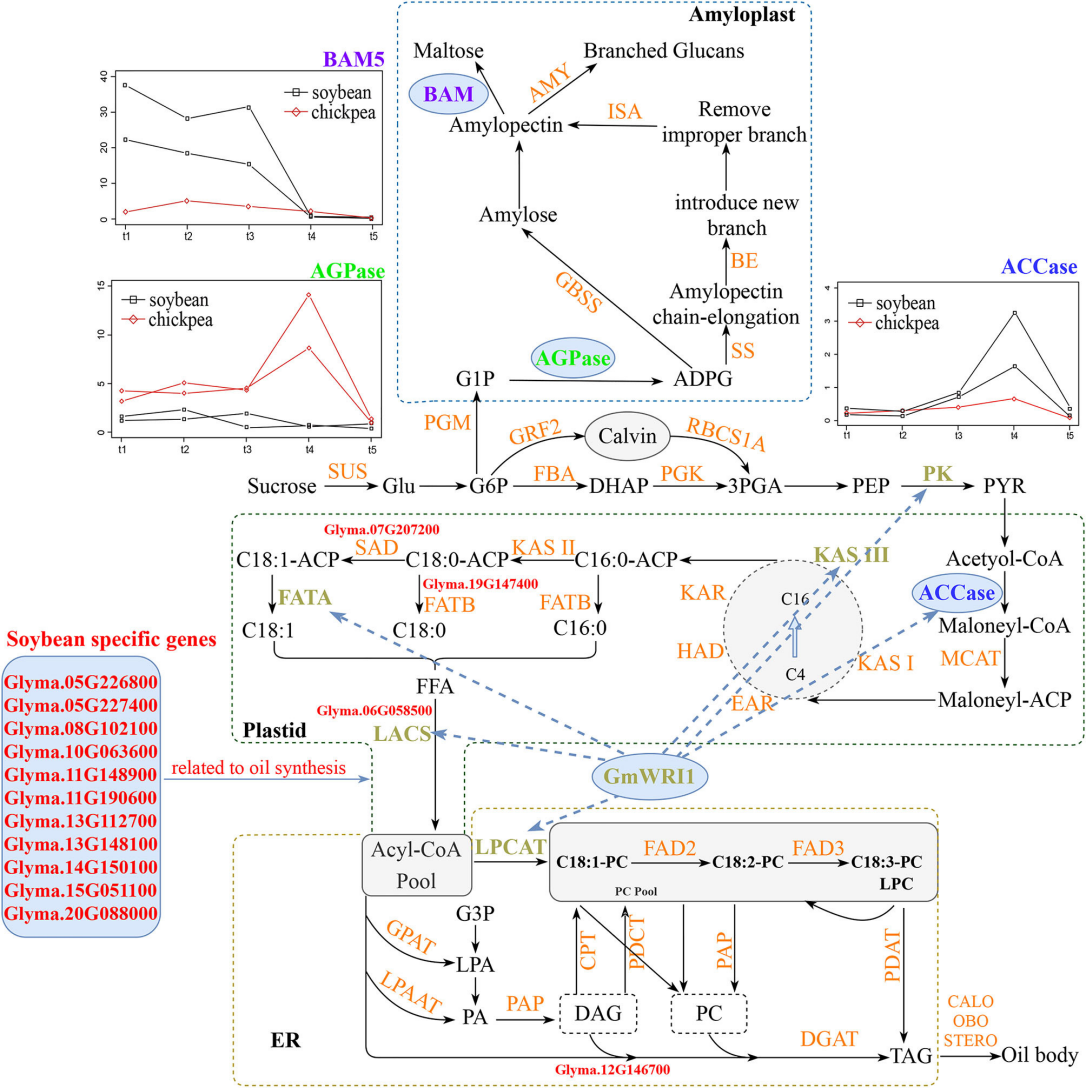
Molecular mechanisms for the difference in seed oil and starch content between soybean and chickpea. In soybean, soybean-specific fatty acid synthesis genes (red), common highly expressed ACCase (blue), and common oil synthesis-related genes *ACCase, KAS, KAR, ACP*, and *LACS* co-expressed with *WRI1* (brown) are responsible for high seed oil content, while common starch degrading enzyme BAM5 (purple) highly expressed at soybean early seed development stage is responsive for low seed starch content. In chickpea, common AGPase (green) highly expressed at chickpea middle seed development stage is responsible for high seed starch content, while no oil synthesis genes co-expressed with common WRI1 is responsible for low seed oil content. LACS, long-chain acyl-CoA synthetase; AGPase, ADP-glucose pyrophosphorylase; BAM-5, beta-amylase-5; ACCase, acetyl CoA carboxylase.

The above results are summarized in Figures 4, 6.

### Molecular Mechanism of High Starch and Low Oil Content in Chickpea Seed

Although *CaWRI1* in chickpea has a similar expression pattern as its homologous genes *Glyma.08G227700* and *Glyma.5G221600* in soybean, no chickpea oil synthesis-related genes were co-expressed with *CaWRI1*. The possible reason is 24 extra amino acids of the RAYD element of the first AP2 domain of CaWRI1 before the VYL motif. In previous studies, mutant seeds with T-DNA inserted in the 5th exon of *AtWRI1* decreased seed oil content 72% in *Arabidopsis* than in its wild type (An et al., 2017), while *wri1* mutant could not convert glucose and sucrose into precursors of fatty acid biosynthesis during seed development, resulting in the decrease of seed oil content (Focks and Benning, 1998). Thus, it is speculated that the structural variation of CaWRI1 leads to the failure to regulate glycolysis and lipid synthesis-related genes, which may be an important reason for low seed oil content in chickpea.

As described above, *AGPase* had more than 7 times relative expressional level in chickpea than in soybean. Its high expression is likely to catalyze more Glc-1-P to ADP glucose (Crevillén et al., 2005). As evidenced in rice (Tuncel et al., 2014) and potato (Hajirezaei et al., 2003), AGPase could effectively control starch flux. In molecular biology, the 100-grain weight of maize seeds with overexpressing AGPase was increased by 15%, and seed starch content was increased by 74% compared to its wild type (Li et al., 2011), while the weight of wheat seeds with overexpressing cytoplasmic AGPase large subunit gene was increased by 9.1%, and seed starch content was increased by 9.6% compared to its wild type (Kang et al., 2013). Thus, high expression of AGPase is more likely to result in higher seed starch content in chickpea.

The above results are summarized in Figure 6.

Yang et al. (2015) performed the comparative genomic and transcriptome analyses of seed starch and oil content between adzuki bean and soybean in the genome sequencing project of adzuki bean (*Vigna angularis*). In this project, they found no significant variation of starch biosynthesis genes in the two genomes, meanwhile, they checked the expression levels of five key genes that encode the proteins involved in the conversion from starch to oil synthesis. Although Yang et al. (2015) did not compare starch synthesis-related genes, key TFs, miRNAs, and their regulation networks, oil- and starch synthesis-related genes, TFs, miRNAs, and their regulatory networks in soybean and chickpea were investigated in this study to obtain the molecular mechanisms for the differences in seed oil and starch content.

### Breeding by Design for High Seed Oil Content in Chickpea

As pointed out by Dragičevi et al. (2015), increasing seed oil content in chickpea can reduce seed phytic acid content and improve its digestibility. However, there is low seed oil content in the present chickpea cultivars. To address this issue, it is necessary to improve seed oil content in chickpea. Based on the above results, two genetic improvement methods are proposed.

First, deleting the 24 extra amino acids of the RAYD element in Ca_15711 (CaWRI1) may make the edited CaWRI1 co-express with oil synthesis-related genes to increase seed oil content in chickpea. In this study, we found that the relative expressional level of Ca_15711 (CaWRI1) at stage S3 in chickpea is higher than those of soybean genes *Glyma.15G221600* and *Glyma.08G227700*, homologous to Ca_15711, at stage R4 (Figure 5B). Shen et al. (2010) had provided transgenic evidence. In detail, seed oil content in maize was significantly higher in transgenic *ZmWRI1* endosperm (0.81%) than in null endosperm (0.47%), but seed starch content was reduced approximately 60%, although the expression of *ZmWRI1* does not affect protein content in embryo and grain yield, indicating that *ZmWRI1* may enhance oil biosynthesis by reducing carbon metabolites in starch biosynthesis.

Second, increasing the expression of miRNA159 and miRNA319 in chickpea may decrease the expression of *MYB33*, which downregulates starch synthesis-related enzymes AGPase, SS, and ISA, making more carbon metabolites flow into fatty acid synthesis. Hu et al. (2021) provided experimental evidence. In detail, Zma-miR159k-3p could negatively regulate the expression of *ZmMYB138* and *ZmMYB115*, homologous to *AtMYB33*, in maize endosperm, while *ZmMYB138* and *ZmMYB115* may be positively correlated with amylopectin and amylose content in maize endosperm, respectively. Thus, Zma-miR159k-3p may indirectly regulate starch synthesis in maize endosperm by regulating the expression of *ZmMYBs*.

## Data Availability Statement

The original contributions presented in the study are included in the article/Supplementary Materials, further inquiries can be directed to the corresponding author.

## Author Contributions

Y-MZ conceived and supervised the study. KC, Y-FP, L-ML, and H-QZ carried out the experimental works and analyzed the data. KC, Y-FP, and Y-MZ wrote and revised the manuscript. All authors read and approved the final manuscript.

## Funding

This work was supported by grants from the National Natural Science Foundation of China (32070557 and 31871242) and the Huazhong Agricultural University Scientific and Technological Self-innovation Foundation (2014RC020).

## Conflict of Interest

The authors declare that the research was conducted in the absence of any commercial or financial relationships that could be construed as a potential conflict of interest.

## Publisher's Note

All claims expressed in this article are solely those of the authors and do not necessarily represent those of their affiliated organizations, or those of the publisher, the editors and the reviewers. Any product that may be evaluated in this article, or claim that may be made by its manufacturer, is not guaranteed or endorsed by the publisher.
