# Supplementary material for: Integrated Transcriptomic and Bioinformatics Analyses Reveal the Molecular Mechanisms for the Differences in Seed Oil and Starch Content Between Glycine max and Cicer arietinum
Source: Front Plant Sci. 2021 Oct 26;12:743680. doi: 10.3389/fpls.2021.743680 (PMC8576049; doi:10.3389/fpls.2021.743680)
Supplement: Supplementary file 2 [file Data_Sheet_2.pdf]

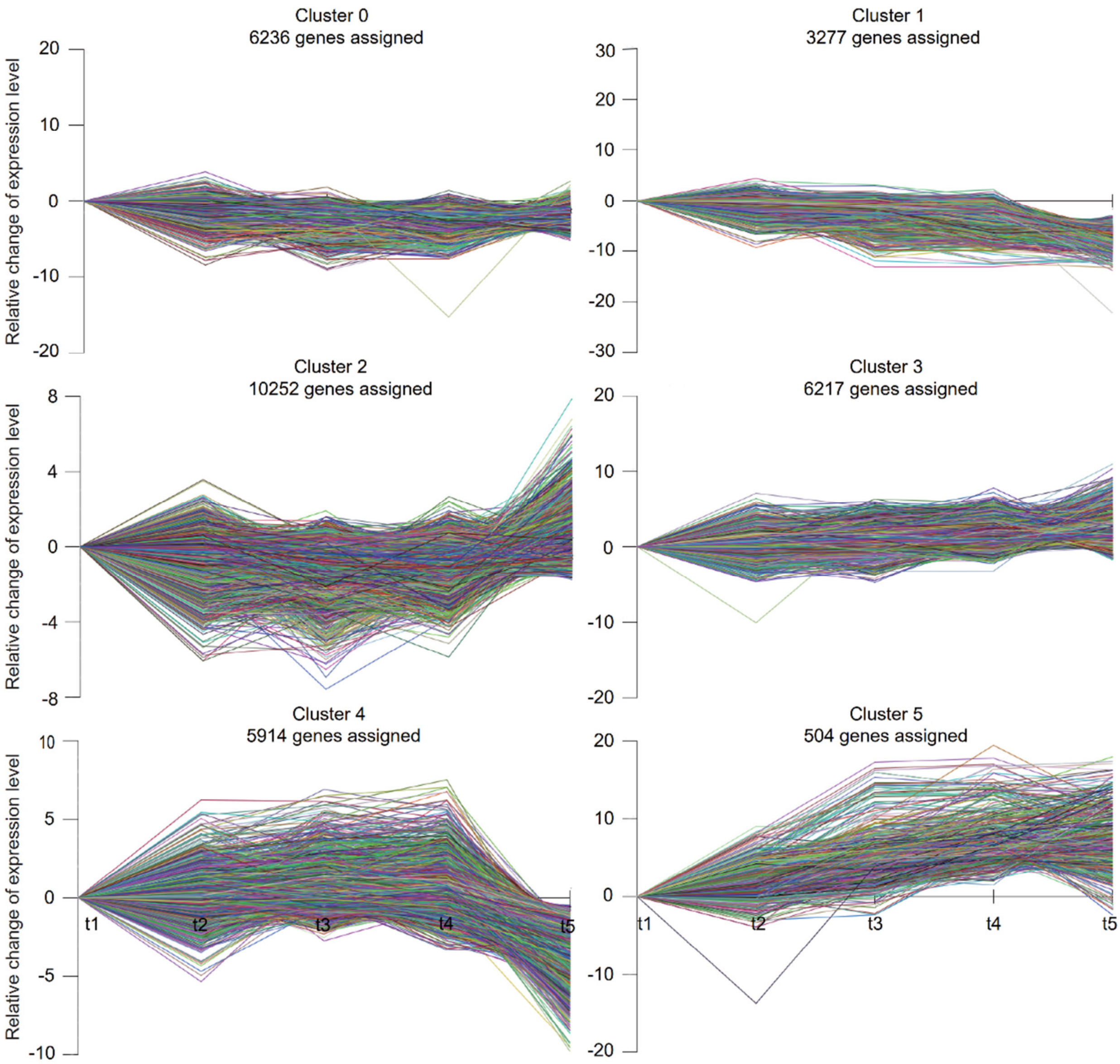

FIGURE S1 | The expressional profiles of all the common genes in six clusters of soybean and chickpea at stages t1 ~ t5

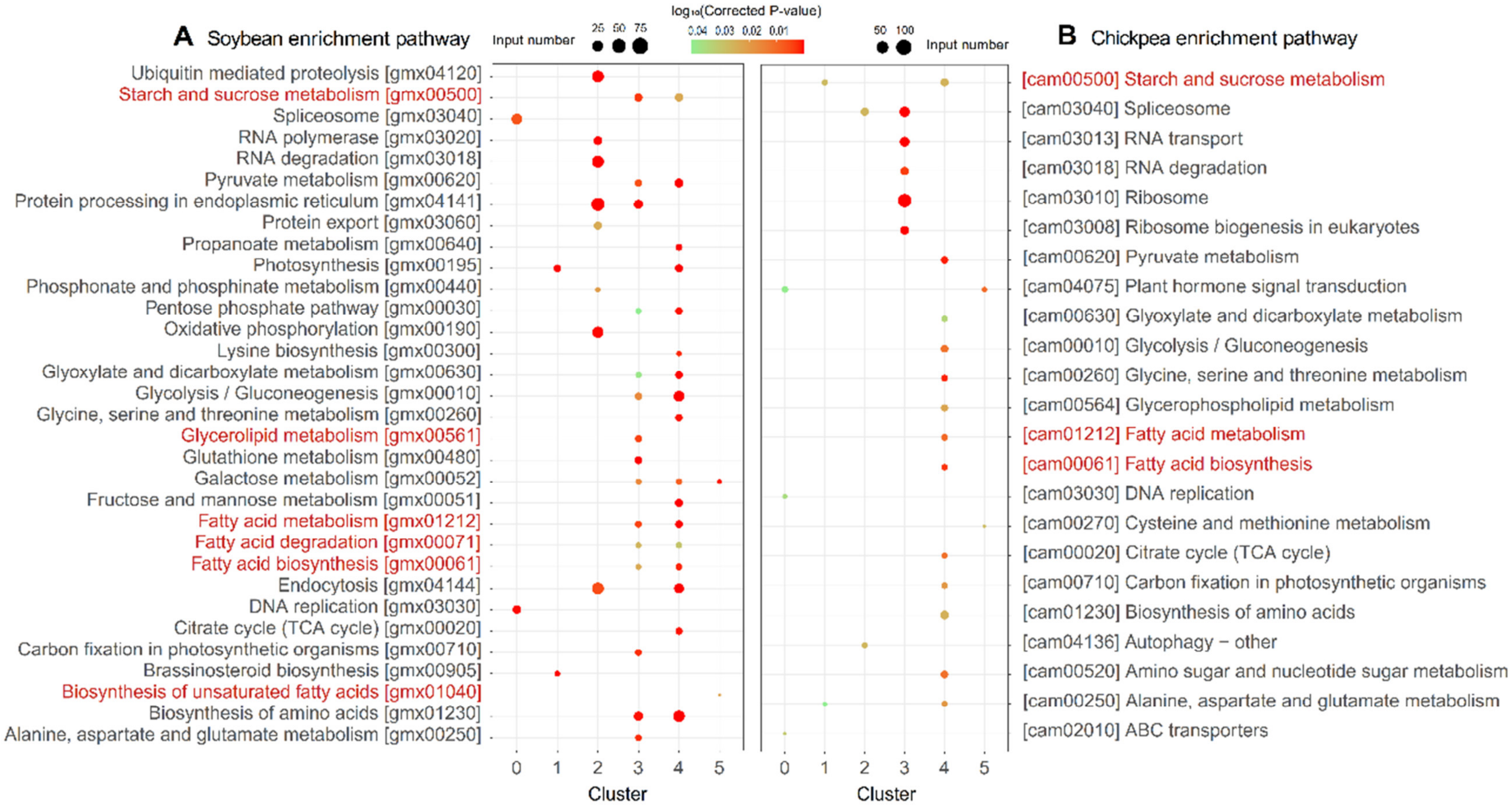

FIGURE S2 | The KEGG pathway enrichment analysis for all the common soybean (A) and chickpea (B) genes in each cluster. The pathways marked with red color were related to oil- and/or starch-metabolisms.

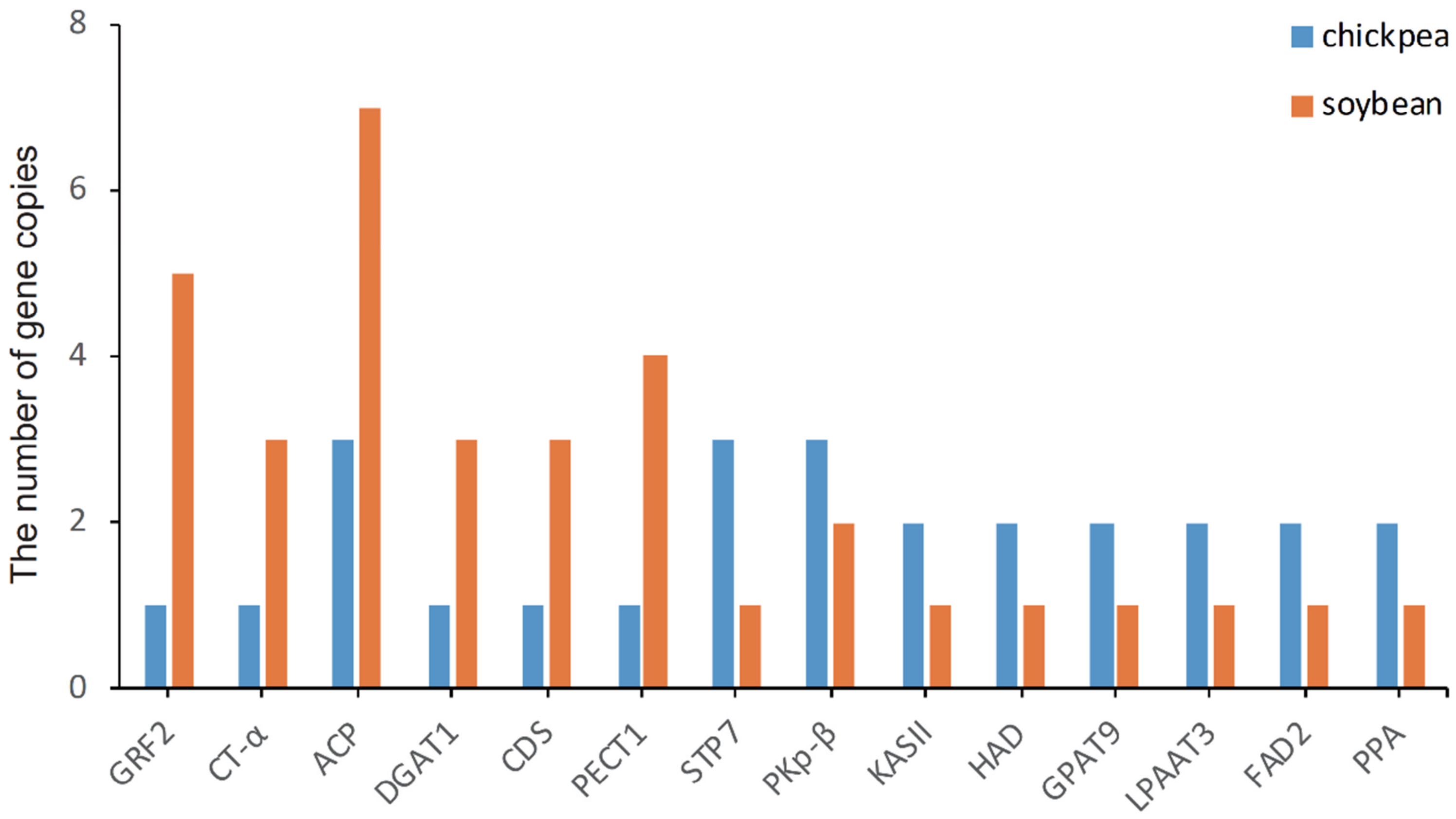

FIGURE S3 | The copy number variation analysis for carbon-, lipid-, and starch-metabolism-related genes between soybean and chickpea

FIGURE S4 | Relative expressional levels of common genes G6PDH (A), RBCS (B), GPAT (C), and LPCAT (D) in soybean and chickpea at five seed development stages

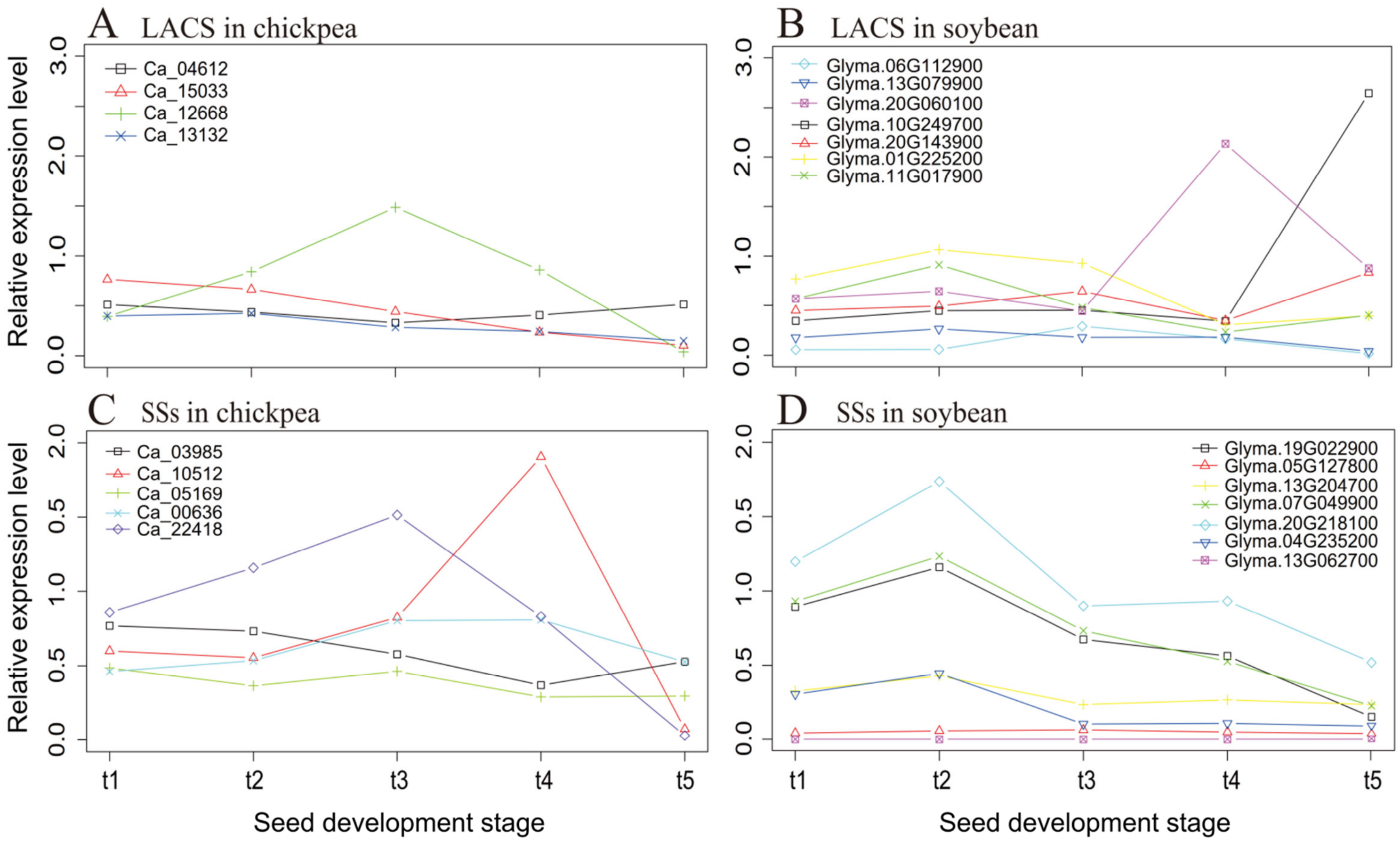

FIGURE S5 | Relative expressional levels of common genes LACS (A and B) and SSs (C and D) in soybean (B and D) and chickpea (A and C) at five seed development stages

Table S2 | Numbers of homologous blocks and collinear gene pairs within or between soybean and chickpea genomes

Homologous Blocks within and among genome	BL > 5	BL > 10	BL > 50	ACGP	LDB	LDB on chromosomes
Soybean	29101/472	26689/305	21772/152	61.65, 87.5, 143.24	1072	Chr03- Chr19
Chickpea	1214/45	830/21	380/5	26.98, 39.52, 76	123	Ca7- Ca8
Soybean vs chickpea	33027/596	29667/363	22826/151	55.41, 81.73, 151.17	675	Chr10- Ca4

BL: block_length; ACGP: average collinear gene pairs per block under various block length; LDB: the number of collinear gene pairs in the longest duplicated block

Table S3 | Comparison of common and specific protein-sequence-based genes in soybean and chickpea

	Common genes				Specific genes
	One soybean copy & multiple chickpea copies	One chickpea copy & multiple soybean copies	Multiple copies for the two species	One copy for the two species	
No. of gene families	335	8675	3261	2396	
No. of proteins	1546	30301	21007	4792	26667
No. of soybean proteins	335	21626	13030	2396	18657
Percentage of soybean proteins (%)	0.60	38.59	23.25	4.28	33.29
No. of chickpea proteins	1211	8675	7977	2396	8010
Percentage of chickpea proteins (%)	4.28	30.69	28.22	8.48	28.33

Table S4 | List of selected genes related to carbohydrate-, lipid-, and starch- metabolisms in *Arabidopsis thaliana*

AT locus ID	Subcellular localization	Gene annotation	Protein / gene abbreviation
		**Sucrose Metabolism**	
AT1G35580	Cytosol	Neutral Invertase	N-INV1
AT4G09510	Cytosol	Neutral Invertase	Nl-INV2
AT1G22650	Cytosol	putative N-invertase	putN-INV
AT1G72000	Cytosol	putative N-invertase	putN-INV
AT4G34860	Cytosol	putative N-invertase	putN-INV
			**N-INV**
0AT5G2251	Plastid	putative N-invertase	putN-INV
AT3G05820	Mit/Plastid	putative N-invertase	putN-INV
AT3G06500	Mit/Plastid	putative N-invertase	putN-INV
			**N-INV**
AT2G36190	Cell Wall	Cell Wall Invertase	CWINV4
AT3G13784	Cell Wall	Cell Wall Invertase	CWINV5
AT3G13790	Cell Wall	Cell Wall Invertase	CWINV1
AT3G52600	Cell Wall	Cell Wall Invertase	CWINV2
			**CW-INV**
AT1G12240	Vacuole	beta-fructosidase	BFRUCT4
AT1G62660	Vacuole	beta-fructosidase	BFRUCT3
			**Vacuolar-INV**
AT1G73370	Cytosol	Sucrose Synthase	SUS6
AT3G43190	Cytosol	Sucrose Synthase	SUS4
AT4G02280	Cytosol	Sucrose Synthase	SUS3
AT5G20830	Cytosol	Sucrose Synthase	SUS1
AT5G37180	Cytosol	Sucrose Synthase	SUS5
AT5G49190	Cytosol	Sucrose Synthase	SUS2
			**SuSy**
		**Plastid Transporters**	
AT1G09960	Vacuole	Sucrose-proton symporter	SUC4
AT1G22710	Plasma Membrane	Sucrose-proton symporter	SUC2
AT1G71880	Plasma Membrane	Sucrose-proton symporter	SUC1
AT2G02860	Plasma Membrane	Sucrose-proton symporter	SUC3
AT2G14670	Plasma Membrane	Sucrose-proton symporter	SUC8
AT5G43610	Plasma Membrane	Sucrose-proton symporter	SUC6
AT1G11260	Plasma Membrane	Sugar-transporter	STP1
AT1G77210	Plasma Membrane	Sugar-transporter	STP14
AT3G05960	Plasma Membrane	Sugar-transporter	STP6
AT3G19930	Plasma Membrane	Sugar-transporter	STP4
AT4G02050	Plasma Membrane	Sugar-transporter	STP7
AT4G21480	Plasma Membrane	Sugar-transporter	STP12
AT5G23270	Plasma Membrane	Sugar-transporter	STP11
AT5G26250	Plasma Membrane	Sugar-transporter	STP8
AT5G26340	Plasma Membrane	Sugar-transporter	STP13 MSS1
AT5G61520	Plasma Membrane	Sugar-transporter	STP3
			**STP**
		**Glycolysis**	

AT3G03250	Cytosol	UDP-glucose pyrophosphorylase	UGP1
AT5G17310	Cytosol	UDP-glucose pyrophosphorylase	UGP2
			**UGP**
AT3G56040	Plastid	UDP-glucose pyrophosphorylase	UGP3
AT1G23190	Cytosol	phosphoglucomutase	PGM
AT1G70730	Cytosol	phosphoglucomutase	PGM
			**PGM**
AT1G70820	Plastid	phosphoglucomutase	PGM2
AT5G51820	Plastid	phosphoglucomutase	PGM1
			**PGM**
AT2G19860	Cytosol	Hexokinase	HXK2
AT4G29130	Cytosol	Hexokinase	HXK1
			**HXK**
AT1G47840	Plastid	Hexokinase	HXK3
AT1G06020	Cytosol	Probable Fructokinase	put FK-3
AT2G31390	Cytosol	Probable Fructokinase	putFK-1
AT3G59480	Cytosol	Probable Fructokinase	putFK-4
AT4G10260	Cytosol	Probable Fructokinase	putFK-5
AT5G51830	Cytosol	protein pfkB-type carbohydrate kinase family	pfkB-type
			**FK**
AT1G66430	Plastid	protein pfkB-type carbohydrate kinase family	FK
AT5G42740	Cytosol	isomerase/glucose-6-phosphate phospho-glucose (Glc) isomerase	PGI
AT4G24620	Plastid	isomerase/glucose-6-phosphate phospho-glucose (Glc) isomerase	PGI
AT2G22480	Plastid	phosphofructokinase-6	PFK5
AT5G61580	Plastid	phosphofructokinase-6	PFK4
			**PFK**
AT4G26270	Cytosol	phosphofructokinase-6	PFK3
AT4G29220	Cytosol	phosphofructokinase-6	PFK1
AT4G32840	Cytosol	phosphofructokinase-6	PFK6
AT5G47810	Cytosol	phosphofructokinase-6	PFK2
AT5G56630	Cytosol	phosphofructokinase-6	PFK7
			**PFK**
AT1G12000	Cytosol	pyrophosphate--fructose-6-phosphate 1-phosphotransferase beta subunit, putative	PFP-β
AT4G04040	Cytosol	diphosphate-fructose-6-phosphate 1-phosphotransferase activity	PFP-β
AT1G20950	Cytosol	pyrophosphate--fructose-6-phosphate 1-phosphotransferase-related	PFP-α
AT1G76550	Cytosol	complex, pyrophosphate-dependent phosphofructokinase alpha-subunit complex	PFP-α
			**PFP**
AT1G18270	Cytosol	fructose-bisphosphate aldolase	FBA
AT2G36460	Cytosol	fructose-bisphosphate aldolase	FBA
AT3G52930	Cytosol	fructose-bisphosphate aldolase	FBA
AT4G26530	Cytosol	fructose-bisphosphate aldolase	FBA
			**FBA**
AT2G21330	Plastid	fructose-bisphosphate aldolase	FBA1
AT4G38970	Plastid	fructose-bisphosphate aldolase	FBA2
AT2G01140	Plastid/Mit	fructose-bisphosphate aldolase	FBA
			**FBA**

AT3G55440	Cytosol	triosephosphate isomerase	TPI
AT2G21170	Plastid	triosephosphate isomerase	TPI
AT2G24270	Cytosol	non-phosphorylating NADP-dependent glyceraldehyde-3-phosphate dehydrogenase	NADP GAPDH
AT1G12900	Plastid	Subunit 2 glyceraldehyde-3-phosphate dehydrogenase A	GapA-2
AT3G26650	Plastid	Subunit 1 glyceraldehyde-3-phosphate dehydrogenase A	GapA-1
			**GapA**
AT1G42970	Plastid	Subunit glyceraldehyde-3-phosphate dehydrogenase B	GapB
AT1G13440	Cytosol	C-2 glyceraldehyde-3-phosphate dehydrogenase	GapC2
AT3G04120	Cytosol	C-1 glyceraldehyde-3-phosphate dehydrogenase	GapC1
			**GapC**
AT1G16300	Plastid	C-2 Plastid glyceraldehyde-3-phosphate dehydrogenase isoform	GapCp-2
AT1G79530	Plastid	C-1 plastid glyceraldehyde-3-phosphate dehydrogenase isoform	GapCp-1
			**GapC**
AT1G79550	Cytosol	phosphoglycerate kinase	PGK
AT3G45090	Cytosol	phosphoglycerate kinase	PGK
AT5G60760	Cytosol	phosphoglycerate kinase	PGK
			**PGK**
AT1G56190	Plastid	phosphoglycerate kinase	PGK
AT3G12780	Plastid	phosphoglycerate kinase	PGK
AT5G61450	Plastid	phosphoglycerate kinase	PGK
			**PGK**
AT1G09780	Cytosol	phosphoglycerate bisphosphoglycerate-independent-2,3 mutase	i-PGM
AT3G08590	Cytosol	phosphoglycerate bisphosphoglycerate-independent-2,3 mutase	i-PGM
AT3G30841	Cytosol	phosphoglycerate bisphosphoglycerate-independent-2,3 mutase	i-PGM
AT4G09520	Cytosol	phosphoglycerate bisphosphoglycerate-independent-2,3 mutase	i-PGM
			**i-PGM**
AT1G22170	Plastid	phosphoglycerate/bisphosphoglycerate mutase	d-PGM
AT1G78050	Plastid	phosphoglycerate/bisphosphoglycerate mutase	d-PGM
			**d-PGM**
AT2G29560	Cytosol	phosphoenolpyruvate enolase	ENOC
AT2G36530	Cytosol/Plastid/Mit	phosphoenolpyruvate enolase	ENO2
			**ENO**
AT1G74030	Plastid	phosphoenolpyruvate enolase	ENO1
AT2G36580	Cytosol	pyruvate kinase	PK
AT3G04050	Cytosol	pyruvate kinase	PK
AT3G25960	Cytosol	pyruvate kinase	PK
AT3G52990	Cytosol	pyruvate kinase	PK
AT4G26390	Cytosol	pyruvate kinase	PK
AT5G08570	Cytosol	pyruvate kinase	PK
AT5G56350	Cytosol	pyruvate kinase	PK
AT5G63680	Cytosol	pyruvate kinase	PK
			**PK**
AT3G49160	Plastid	pyruvate kinase	PK
AT1G32440	Plastid	pyruvate kinase beta subunit	PKp3/PKp-β2

AT5G52920	Plastid	pyruvate kinase beta subunit	PKp2/PKp-β1
AT3G22960	Plastid	pyruvate kinase alpha subunit	PKp1/PKp-α
			**PK**
AT1G53310	Cytosol	phosphoenolpyruvate carboxylase	PEPC1
AT1G68750	Cytosol	phosphoenolpyruvate carboxylase	PEPC4
AT2G42600	Cytosol	phosphoenolpyruvate carboxylase	PEPC2
AT3G14940	Cytosol	phosphoenolpyruvate carboxylase	PEPC3
		**Plastid Transporters**	
AT5G46110	Plastid	triose Phosphate Translocator	TPT
AT3G01550	Plastid	phosphoenolpyruvate/phosphate translocator	PPT2
AT5G33320	Plastid	phosphoenolpyruvate/phosphate translocator	PPT1
AT1G61800	Plastid	glucose6-Phosphate/phosphate transporter 2	GPT2
AT5G54800	Plastid	glucose6-Phosphate/phosphate transporter 1	GPT1
AT5G17630	Plastid	xylulose-5-phosphate/phosphate translocator	XPT
AT5G16150	Plastid	glucose transporter	GLT1
AT1G15500	Plastid	nucleotide transporter 1	NTT2
AT1G80300	Plastid	nucleotide transporter 1	NTT1
AT5G12860	Plastid	dicarboxylate transporter	DiT1
AT5G64290	Plastid	dicarboxylate transporter	DiT2.1
AT5G64280	Plastid	dicarboxylate transporter	DiT2.2
		**Pentose Phosphate Pathway and Calvin Cycle**	
AT3G27300	Cytosol	glucose-6-phosphate dehydrogenase	G6PD5
AT5G40760	Cytosol	glucose-6-phosphate dehydrogenase	G6PD6
			**G6PDH**
AT1G09420	Plastid	glucose-6-phosphate dehydrogenase	G6PD4
AT1G24280	Plastid	glucose-6-phosphate dehydrogenase	G6PD3
AT5G13110	Plastid	glucose-6-phosphate dehydrogenase	G6PD2
AT5G35790	Plastid	glucose-6-phosphate dehydrogenase	G6PD1
			**G6PDH**
AT1G13700	Cytosol	phosphogluconolactonase-6	PGL1
AT5G24420	Cytosol	phosphogluconolactonase-6	PGL5
			**PGL**
AT5G24400	Plastid	phosphogluconolactonase-6	PGL3
AT3G02360	Peroxisome/cytosol?	phosphogluconate dehydrogenase-6	PGDH6
AT5G41670	Plastid/Mit	phosphogluconate dehydrogenase-6	PGDH6
AT1G64190	Plastid	phosphogluconate dehydrogenase-6	PGDH6
			**PGDH6**
AT1G63290	Cytosol	ribulose-phosphate 3-epimerase	RPE
AT3G01850	Cytosol	ribulose-phosphate 3-epimerase	RPE
			**RPE**
AT5G61410	Plastid	ribulose-phosphate 3-epimerase	RPE
AT1G71100	Cytosol	ribose-5-phosphate isomerase	RPI
AT2G01290	Cytosol	ribose-5-phosphate isomerase	RPI
			**RPI**
AT3G04790	Plastid	ribose-5-phosphate isomerase	RPI
AT5G44520	Plastid	ribose-5-phosphate isomerase	RPI
			**RPI**

AT2G45290	Plastid	transketolase	transketolase
AT3G60750	Plastid	transketolase	transketolase
			**TK**
AT1G12230	Plastid	transaldolase	transaldolase
AT5G13420	Plastid	transaldolase	transaldolase
			**TA**
AT1G67090	Plastid	RIBULOSE BISPHOSPHATE CARBOXYLASE SMALL CHAIN 1A	RBCS-1A
AT5G38410	Plastid	chain 3B ribulose bisphosphate carboxylase small	RBCS-3B
AT5G38420	Plastid	chain 2B ribulose bisphosphate carboxylase small	RBCS-2B
AT5G38430	Plastid	chain 1B ribulose bisphosphate carboxylase small	RBCS-1B
			**RBCS**
AT1G32060	Plastid	Phosphoribulokinase	PRK
		**Organic Acid and TCA**	
AT4G26970	mito/perox	aconitase B	
AT4G35830	mito/perox	aconitase B	
AT2G05710	mito/perox	aconitate hydratase	
AT3G58740	perox	citrate (SI)-synthase	CSY1
AT2G42790	perox	citrate (SI)-synthase	CSY3
AT2G44350	mitochondria	citrate (SI)-synthase	CSY4
AT3G60100	mitochondria	citrate (SI)-synthase	CSY5
AT5G50950	cytosol	fumarase	FUM2
AT2G47510	mitochondria	fumarate hydratase	FUM1
AT5G03290	mitochondria	NAD+-dependent isocitrate dehydrogenase	IDH-V
AT3G09810	mitochondria	NAD+-dependent isocitrate dehydrogenase catalytic subunit	IDH-VI
AT2G17130	mitochondria	NAD+-dependent isocitrate dehydrogenase regulatory subunit	IDH-II
AT4G35260	mitochondria	NAD+-dependent isocitrate dehydrogenase regulatory subunit	IDH-I
AT4G35650	mitochondria	NAD+-dependent isocitrate dehydrogenase regulatory subunit	IDH-III
AT1G54340	perox?	NADP+-dependent isocitrate dehydrogenase regulatory subunit	ICDH
AT1G65930	cytosol	NADP+-dependent isocitrate dehydrogenase regulatory subunit	CICDH
AT5G14590	chl/mito?	NADP+-dependent isocitrate dehydrogenase regulatory subunit	
AT1G24180	mitochondria	pyruvate dehydrogenase (acetyl-transferring)	
AT1G59900	mitochondria	pyruvate dehydrogenase (acetyl-transferring)	
AT5G50850	mitochondria	pyruvate dehydrogenase (acetyl-transferring)	
AT2G18450	mitochondria	succinate dehydrogenase (ubiquinone)	
AT3G27380	mitochondria	succinate dehydrogenase (ubiquinone)	
AT5G65165	mitochondria	succinate dehydrogenase (ubiquinone)	
AT1G08480	mitochondria	succinate:ubiquinone oxidoreductase	
AT1G47420	mitochondria	succinate:ubiquinone oxidoreductase	
AT2G46390	mitochondria	succinate:ubiquinone oxidoreductase	
AT2G46505	mitochondria	succinate:ubiquinone oxidoreductase	
AT3G47833	mitochondria	succinate:ubiquinone oxidoreductase	
AT5G40650	mitochondria	succinate:ubiquinone oxidoreductase	
AT5G62575	mitochondria	succinate:ubiquinone oxidoreductase	
AT5G66760	mitochondria	succinate:ubiquinone oxidoreductase	
AT5G08300	mitochondria	succinate-CoA ligase alpha subunit	

AT5G23250	mitochondria	succinate-CoA ligase alpha subunit	
AT2G20420	mitochondria	succinate-CoA ligase beta subunit	
AT1G04410	Cytosol	NAD/P-dependent malate dehydrogenase	MDH
AT5G43330	Cytosol	NAD/P-dependent malate dehydrogenase	MDH
AT5G56720	Cytosol	NAD/P-dependent malate dehydrogenase	MDH
AT3G47520	Plastid	NAD/P-dependent malate dehydrogenase	MDH
AT5G58330	Plastid	NAD/P-dependent malate dehydrogenase	MDH
AT1G53240	Mitochondria	NAD/P-dependent malate dehydrogenase	MDH
AT3G15020	Mitochondria	NAD/P-dependent malate dehydrogenase	MDH
AT2G22780	Peroxisome	peroxisomal NAD-malate dehydrogenase	PMDH1
AT5G09660	Peroxisome	peroxisomal NAD-malate dehydrogenase	PMDH2
AT2G19900	Cytosol	NADP-malic enzyme	NADP-ME1
AT5G11670	Cytosol	NADP-malic enzyme	NADP-ME2
AT5G25880	Cytosol	NADP-malic enzyme	NADP-ME3
AT1G79750	Plastid	NADP-malic enzyme	NADP-ME4
AT2G13560	Mitochondria	NAD-malic enzyme	NAD-ME1
AT4G00570	Mitochondria	NAD-malic enzyme	NAD-ME2
		**Other Carbohydrate Metabolism**	
AT3G46970	Plastid	phosphorylase	
AT5G04360	Plastid	pullulanase	
AT4G01900	Plastid	GLNB1 homolog; regulates ACCase activity	PII
AT4G15530	Plastid	Pyruvate orthophosphate dikinase	Plastid-PPDK
AT3G04530	Cytosol	Phosphoenolpyruvate carboxykinase	PEPCK3
AT5G65690	Cytosol	Phosphoenolpyruvate carboxykinase	PEPCK2
AT4G37870	Cytosol	Phosphoenolpyruvate carboxykinase	PEPCK1
		**Plastid Fatty Acid Synthesis from Pyruvate**	
AT1G01090	Plastid	E1-alpha component of Pyruvate Dehydrogenase Complex	PDH-E1α
AT1G30120	Plastid	E1-beta component of Pyruvate Dehydrogenase Complex	PDH-E1β
AT2G34590	Plastid	E1-beta component of Pyruvate Dehydrogenase Complex	PDH-E1β
AT1G34430	Plastid	Dihydrolipoamide Acetyltransferase, E2 component of Pyruvate Dehydrogenase Complex	LTA1
AT3G25860	Plastid	Dihydrolipoamide Acetyltransferase, E2 component of Pyruvate Dehydrogenase Complex	LTA2
AT3G16950	Plastid	Dihydrolipoamide Dehydrogenase, E3 component of Pyruvate Dehydrogenase Complex	LPD1
AT4G16155	Plastid	Dihydrolipoamide Dehydrogenase, E3 component of Pyruvate Dehydrogenase Complex	LPD2
			**PDHC**
AT2G38040	Plastid	Heteromeric Carboxyltransferase- alpha; Subunit of ACCase	CT-α
ATCG00500	Plastid	Heteromeric ACCase Carboxyltransferase- beta; Subunit of	CT-β
AT5G15530	Plastid	Heteromeric ACCase Biotin Carboxyl Carrier Protein of	BCCP2
AT5G16390	Plastid	Heteromeric ACCase Biotin Carboxyl Carrier Protein of	BCCP1
			**BCCP**
AT5G35360	Plastid	Biotin Carboxylase of Heteromeric ACCase	BC
			**ACCase**
AT2G30200	Plastid	Malonyl-CoA : ACP Malonyltransferase	MCMT
AT5G46290	Plastid	Ketoacyl-ACP Synthase I	KASI
AT1G74960	Plastid	Ketoacyl-ACP Synthase II	KASII (fab1)
AT1G62640	Plastid	Ketoacyl-ACP Synthase III	KASIII
AT1G24360	Plastid	Ketoacyl-ACP Reductase	KAR

AT1G62610	Plastid	Ketoacyl-ACP Reductase	KAR
AT3G46170	Plastid	Ketoacyl-ACP Reductase	KAR
AT3G55290	Plastid	Ketoacyl-ACP Reductase	KAR
			**KAR**
AT2G22230	Plastid	Hydroxyacyl-ACP Dehydratase	HAD
AT5G10160	Plastid	Hydroxyacyl-ACP Dehydratase	HAD
			**HAD**
AT2G05990	Plastid	Enoyl-ACP Reductase	ENR1 (MOD1)
AT1G54580	Plastid	Acyl Carrier Protein	ACP2
AT1G54630	Plastid	Acyl Carrier Protein	ACP3
AT3G05020	Plastid	Acyl Carrier Protein	ACP1
AT4G25050	Plastid	Acyl Carrier Protein	ACP4
AT5G27200	Plastid	Acyl Carrier Protein	ACP5
			**ACP**
AT1G43800	Plastid	Stearoyl-ACP Desaturase	DES6
AT2G43710	Plastid	Stearoyl-ACP Desaturase	FAB2
AT3G02610	Plastid	Stearoyl-ACP Desaturase	DES2
AT3G02620	Plastid	Stearoyl-ACP Desaturase	DES4
AT3G02630	Plastid	Stearoyl-ACP Desaturase	DES5
AT5G16230	Plastid	Stearoyl-ACP Desaturase	DES3
AT5G16240	Plastid	Stearoyl-ACP Desaturase	DES1
			**SAD**
AT3G25110	Plastid	Acyl-ACP Thioesterase A	FatA
AT4G13050	Plastid	Acyl-ACP Thioesterase A	FatA
			**FATA**
AT1G08510	Plastid	Acyl-ACP Thioesterase B	FATB
AT3G11470	Plastid	Holo-ACP Synthase	HACPS
AT4G14070	Plastid	Acyl-ACP Synthetase	AAE15
AT2G04350	Plastid	Long-Chain Acyl-CoA Synthetase	LACS8
AT1G77590	Plastid envelope	Long-Chain Acyl-CoA Synthetase	LACS9
AT1G36180	Plastid	Acetyl-CoA Carboxylase (Homomeric)	ACC2
		**Eukaryotic Glycerolipid/TAG Synthesis**	
AT2G41540	Cytosol	NAD-dependent Glycerol-3-Phosphate Dehydrogenase	GPDH
AT3G07690	Cytosol	NAD-dependent Glycerol-3-Phosphate Dehydrogenase	GPDH
			**GPDH**
AT5G60620	Endomembrane	Glycerol-3-Phosphate Acyltransferase (mammalian homologue)	GPAT9
AT3G57650	Endomembrane	1-Acylglycerol-3-Phosphate Acyltransferase	LPAAT2
AT1G51260		1-Acylglycerol-3-Phosphate Acyltransferase	LPAAT3
AT1G75020		1-Acylglycerol-3-Phosphate Acyltransferase	LPAAT4
AT3G18850		1-Acylglycerol-3-Phosphate Acyltransferase	LPAAT5
			**LPAAT**
AT3G09560	Endomembrane	Phosphatidate Phosphatase	PAH1
AT5G42870	Endomembrane	Phosphatidate Phosphatase	PAH2
AT4G22550		Phosphatidate Phosphatase	LPP-β
AT3G58490		Long Chain Base 1-Phosphate Phosphatase	LPP-δ
			**PP**
AT1G48300	Endomembrane	Acyl-CoA : Diacylglycerol Acyltransferase	DGAT3
AT2G19450	Endomembrane	Acyl-CoA : Diacylglycerol Acyltransferase	DGAT1

AT3G51520	Endomembrane	Acyl-CoA : Diacylglycerol Acyltransferase	DGAT2
AT5G55340		Wax Synthase -like	DAcT
			**DGAT**
AT5G13640	Endomembrane	Phospholipid : Diacylglycerol Acyltransferase	PDAT1
AT3G44830	Endomembrane	Phospholipid : Diacylglycerol Acyltransferase	PDAT-like/PDAT2
AT4G19860		Phospholipid : Acyl acceptor Acyltransferase	PDAT-related?
			**PDAT**
AT1G80950		1-acylglycerol-3-phosphoethanolamine Acyltransferase	LPEAT1
AT2G45670		1-acylglycerol-3-phosphoethanolamine Acyltransferase	LPEAT2
			**LPEAT**
AT1G12640	Endomembrane	1-acylglycerol-3-phosphocholine Acyltransferase	LPCAT
AT1G63050	Endomembrane	1-acylglycerol-3-phosphocholine Acyltransferase	LPCAT
			**LPCAT**
AT3G15820		Phosphatidylcholine:diacylglycerol cholinephosphotransferase	PDCT/ROD1
AT3G12120	Endomembrane	Oleate Desaturase	FAD2
AT2G29980	Endomembrane	Linoleate Desaturase	FAD3
			**FAD**
AT1G62430		CDP-DAG Synthase	CDS1
AT4G22340		CDP-DAG Synthase	CDP-DAGS
			**CDP-DAG (CDS)**
AT3G25585		Diacylglycerol Cholinephosphotransferase	DAG-CPT
AT1G13560		Diacylglycerol Cholinephosphotransferase	DAG-CPT
			**DAG-CPT**
AT1G71697		Choline Kinase	CK1
AT1G34100	Cytosol (predicted)	Choline Kinase	CK
AT1G74320	Cytosol (predicted)	Choline Kinase	CK
AT4G09760	Cytosol (predicted)	Choline Kinase	CK
			**CK**
AT2G32260	Plasma membrane? (proteomics)	Choline-Phosphate Cytidylyltransferase	CCT1
AT4G15130		Choline-Phosphate Cytidylyltransferase	CCT2
			**CCT**
AT5G53470	Plasma membrane	Acyl CoA Binding Protein	ACBP1
AT4G27780	Plasma membrane	Acyl CoA Binding Protein	ACBP2
AT4G24230	Secretory pathway	Acyl CoA Binding Protein	ACBP3
AT3G05420	Cytosol	Acyl CoA Binding Protein	ACBP4
AT5G27630		Acyl CoA Binding Protein	ACBP5
AT1G31812	Cytosol	Acyl CoA Binding Protein	ACBP6
			**ACBP**
AT4G23850		Long-Chain Acyl-CoA Synthetase	LACS4
AT4G11030		Long-Chain Acyl-CoA Synthetase	LACS5
			**LACS**
AT1G48600		Phosphoethanolamine N-Methyltransferase	CPUORF31
AT1G73600		Phosphoethanolamine N-Methyltransferase	PEAMT
AT3G18000		Phosphoethanolamine N-Methyltransferase	NMT1
			**PEAMT**
AT4G38570		Phosphatidylinositol Synthase	PIS2
AT1G68000		Phosphatidylinositol Synthase	PIS1
			**PIS**

AT4G16700		Phosphatidylserine Decarboxylase	PSD1
AT5G57190		Phosphatidylserine Decarboxylase	PSD2
AT4G25970		Phosphatidylserine Decarboxylase	PSD3
			**PSD**
AT3G55030		Phosphatidylglycerol-Phosphate Synthase	PGPS2
AT1G15110		Base-Exchange-type Phosphatidylserine Synthase	PSS
AT2G26830		Ethanolamine Kinase	EK
AT2G38670		CDP-Ethanoamine Synthase	PECT1
		**Oilbody Storage Proteins**	
AT4G25140	Oilbody	Oil-Body Oleosin	OBO
AT5G40420	Oilbody	Oil-Body Oleosin	OBO
AT3G27660	Oilbody	Oil-Body Oleosin	OBO
AT3G01570	Oilbody	Oil-Body Oleosin	OBO
AT5G51210	Oilbody	Oil-Body Oleosin	OBO
AT1G48990	Oilbody	Oil-Body Oleosin	OBO
AT2G25890	Oilbody	Oil-Body Oleosin	OBO
AT3G18570	Oilbody	Oil-Body Oleosin	OBO
AT5G07530	Oilbody	Pollen-surface Oleosin	PO
			**Oleosin**
AT1G23240	Oilbody	Caleosin	CALO
AT1G23250	Oilbody	Caleosin	CALO
AT1G70670	Oilbody	Caleosin	CALO
AT1G70680	Oilbody	Caleosin	CALO
AT2G33380	Oilbody	Caleosin	CALO
AT4G26740	Oilbody	Caleosin	CALO
AT5G55240	Oilbody	Caleosin	CALO
AT5G29560	Oilbody	Caleosin	CALO
			**Caleosin**
AT5G50590	Oilbody	Steroleosin	STERO
AT5G50600	Oilbody	Steroleosin	STERO
AT5G50700	Oilbody	Steroleosin	STERO
AT5G50770	Oilbody	Steroleosin	STERO
AT4G10020	Oilbody	Steroleosin	STERO
AT5G50690	Oilbody	Steroleosin	STERO
AT3G47350	Oilbody	Steroleosin	STERO
AT3G47360	Oilbody	Steroleosin	STERO
			**Steroleosin**
		**Starch metastarch metabolism**	
AT5G48300	Plastid	ADP-glucose pyrophosphorylase	ApS1
AT1G05610	Plastid	ADP-glucose pyrophosphorylase	ApS2
AT5G19220	Plastid	glucose-1-phosphate adenylyltransferase	ApL1
AT1G27680	Plastid	glucose-1-phosphate adenylyltransferase	ApL2
AT4G39210	Plastid	glucose-1-phosphate adenylyltransferase	ApL3
AT2G21590	Plastid	glucose-1-phosphate adenylyltransferase	ApL4
AT2G39930	Plastid	Isoamylase	ISA1
AT1G03310	Plastid	Isoamylase	ISA2
AT4G09020	Plastid	Isoamylase	ISA3
AT5G24300	Plastid	starch synthase	SSI
AT3G01180	Plastid	starch synthase	SSII

AT1G11720	Plastid	starch synthase	SSIII
AT4G18240	Plastid	starch synthase	SSIV
AT1G32900	Plastid	Granule-bound starch synthase	GBSS
AT5G03650	Plastid	1,4-alpha-glucan branching enzyme	BE1
AT3G20440	Plastid	1,4-alpha-glucan branching enzyme	BE2
AT2G36390	Plastid	1,4-alpha-glucan branching enzyme	BE3
AT5G09650	Plastid	pyrophosphorylase 6	PPA6
AT1G01050	Cytosol	pyrophosphorylase 1	PPA1
AT2G18230	Cytosol	pyrophosphorylase 2	PPA2
AT2G46860	Cytosol	pyrophosphorylase 3	PPA3
AT3G53620	Cytosol	pyrophosphorylase 4	PPA4
AT4G01480	Cytosol	pyrophosphorylase 5	PPA5
AT3G29320	Plastid	alpha-glucan phosphorylase 1	PHS1
AT3G46970	Cytosol	alpha-glucan phosphorylase 2	PHS2
AT4G25000	Plastid	alpha-amylase	AMY1
AT1G76130	Plastid	alpha-amylase	AMY2
AT1G69830	Plastid	alpha-amylase	AMY3
AT3G23920	Plastid	beta-amylase	BAM1
AT4G00490	Plastid	beta-amylase	BAM2
AT4G17090	Plastid	beta-amylase	BAM3
AT5G55700	Plastid	beta-amylase	BAM4
AT2G45880	Plastid	beta-amylase	BAM7
AT4G15210	Plastid	beta-amylase	BAM5
AT2G32290	Plastid	beta-amylase	BAM6
AT5G45300	Plastid	beta-amylase	BAM8
AT5G18760	Plastid	beta-amylase	BAM9
		**Transcription Factors Associated with Lipid Synthesis**	
AT3G54320	Nucleus	WRINKLED 1; DNA binding / transcription factor	WRI1
AT1G21970	Nucleus	LEAFY COTYLEDON 1; transcription factor	LEC1
AT1G28300	Nucleus	LEAFY COTYLEDON 2; transcription factor	LEC2
AT3G24650	Nucleus	ABA INSENSITIVE 3; DNA binding / transcription activator/ transcription factor	ABI3
AT3G26790	Nucleus	FUSCA 3; DNA binding / transcription factor	FUS3
AT4G32010	Nucleus	HIGH-LEVEL EXPRESSION OF SUGAR-INDUCIBLE GENE-LIKE 1; transcription factor	HSL1
AT2G30470	Nucleus	HIGH-LEVEL EXPRESSION OF SUGAR-INDUCIBLE GENE 2; transcription factor/ transcription repressor	HSL2
AT1G54060	Nuclelus	ASIL1/ 6B-INTERACTING PROTEIN 1-LIKE 1	ASIL1
AT2G25170	Nucleus	group Chromatin remodeling factor of the CHD3	PKL
AT1G16060	Nucleus	Mutant Char.; yeast one-hybrid assays	WRI3
AT1G79700	Nucleus	Mutant Char.; yeast one-hybrid assays	WRI4
		**Transcription Factors Associated with Starch Synthesis**	
AT2G40220	Nucleus	Mutant Char., Promoter binding assays	ABI4
AT1G13600	Nucleus	basic leucine-zipper 58	bZIP58
AT2G02070	Nucleus	indeterminate(ID)-domain 5	IDD5
AT5G06100	Nucleus	MYB DOMAIN PROTEIN 33	MYB33
AT3G11440	Nucleus	MYB DOMAIN PROTEIN 65	MYB65
AT4G28080	Nucleus	Tetratricopeptide repeat (TPR)-like superfamily protein	FLOURY ENDOSPERM2

Table S5 | 97 candidate orthologous groups (OG) related to seed oil and starch synthesis in *Arabidopsis thaliana*, *Glycine max*, and *Cicer arietinum*

Biological process	Predicted annotation	No. of chickpea genes	No. of soybean genes	Clusters	Gene								
	SUS	3	4	OG0001066	AT1G73370	AT5G37180	Ca_13260	Ca_14391	Ca_18036	Glyma.09G167000	Glyma.11G212700	Glyma.14G209900	Glyma.16G217200
	SUC4	1	2	OG0004829	AT1G09960	Ca_21673	Glyma.02G218600	Glyma.04G089200					
	SUC3	1	2	OG0009798	AT2G02860	Ca_19491	Glyma.08G298500	Glyma.18G123400	Psat4g175520				
Carbon fixation	STP7	3	1	OG0005928	AT4G02050	Ca_06757	Ca_06758	Ca_21548	Glyma.15G203300				
	RBCS	2	3	OG0000562	AT1G67090	AT5G38410	AT5G38420	AT5G38430	Ca_16978	Ca_16979	Glyma.13G046200	Glyma.19G046600	Glyma.19G046800
	GRF2	1	5	OG0003608	AT2G22840	AT4G37740	Ca_09216	Glyma.08G265700	Glyma.17G232600	Glyma.17G232700	Glyma.U028600	Glyma.U028700	
	PGM	1	1	OG0008183	AT1G23190	AT1G70730	Ca_10439	Glyma.08G044100					
	PGM1	1	1	OG0012014	AT5G51820	Ca_04520	Glyma.10G241000						
	HXK3	1	2	OG0008081	AT1G47840	Ca_23009	Glyma.14G218800	Glyma.17G257800					
	FK	1	1	OG0003818	AT1G66430	Ca_14365	Glyma.02G245800						
	PGI	1	2	OG0012229	AT5G42740	Ca_10559	Glyma.04G032600	Glyma.19G017200					
	PGI	1	2	OG0009984	AT4G24620	Ca_14997	Glyma.02G212600	Glyma.06G094300					
	PFK5	1	1	OG0007181	AT2G22480	Ca_18116	Glyma.08G280700						
	PFP-β	1	2	OG0003654	AT1G12000	AT4G04040	AT4G08876	Ca_03397	Glyma.07G263800	Glyma.09G007900			
Glycolysis	PFP-α	1	1	OG0006352	AT1G20950	AT1G76550	Ca_14808	Glyma.07G160500					
	FBA	3	5	OG0004631	AT2G21330	AT4G38970	Ca_05550	Ca_15425	Ca_15426	Glyma.11G111100	Glyma.11G111400	Glyma.12G037400	
	GapA	1	2	OG0008125	AT1G12900	Ca_08273	Glyma.16G044900	Glyma.19G106800					
	GapCp	1	2	OG0006079	AT1G16300	AT1G79530	Ca_22679	Glyma.03G092700	Glyma.16G081400				
	PGK	2	3	OG0001511	AT1G56190	AT1G79550	AT3G12780	Ca_22672	Ca_22673	Glyma.08G165400	Glyma.08G165500	Glyma.15G262100	
	PK	3	3	OG0001199	AT2G36580	AT3G52990	Ca_00753	Ca_08402	Ca_20149	Glyma.10G065000	Glyma.13G149800	Glyma.20G189300	
	PKp-β	3	2	OG0004232	AT5G52920	Ca_02280	Ca_04408	Ca_21051	Glyma.01G200200	Glyma.20G164500			
	PKp-α	1	1	OG0008337	AT3G22960	Ca_12150	Glyma.20G211200						

	PEPC4	2	2	OG0003841	AT1G68750	Ca_08446	Ca_16015	Glyma.02G130700	Glyma.10G205500				
	TPT	1	2	OG0008908	AT5G46110	Ca_06344	Glyma.07G201300	Glyma.13G175100					
	PPT2	1	1	OG0013970	AT3G01550	Ca_12580	Glyma.19G005900						
Plastid transporters	PPT1	1	2	OG0014581	AT5G33320	Ca_07481	Glyma.04G184300	Glyma.06G181600					
	GPT	2	4	OG0000938	AT1G61800	AT5G54800	Ca_03358	Ca_24458	Glyma.08G312400	Glyma.13G206700	Glyma.15G105900	Glyma.18G102700	
	G6PDH	1	1	OG0003207	AT3G27300	AT5G40760	Ca_19403	Glyma.19G082300					
Pentose phosphate pathway	G6PDH	2	3	OG0001886	AT1G24280	AT5G13110	AT5G35790	Ca_10123	Ca_18820	Glyma.02G096800	Glyma.07G013800	Glyma.18G284600	
	RPI	1	1	OG0008635	AT5G44520	Ca_08055	Glyma.15G136200						
	MDH	2	1	OG0003043	AT1G04410	AT5G43330	AT5G56720	Ca_12129	Ca_23162	Glyma.02G005500			
Organic acid and TCA	NAD-ME1	1	1	OG0013016	AT2G13560	Ca_11805	Glyma.03G102900						
	NAD-ME2	1	1	OG0004782	AT4G00570	Ca_06238	Glyma.03G014600						
	PDH-E1α	2	1	OG0003029	AT1G01090	Ca_01355	Ca_22416	Glyma.03G261000	Glyma.07G049600	Glyma.16G018300	Glyma.19G260000		
	PDH-E1β	1	1	OG0005785	AT1G30120	AT2G34590	Ca_05041	Glyma.08G096300					
	CT-α	1	3	OG0007457	AT2G38040	Ca_26217	Glyma.18G195700	Glyma.18G195900	Glyma.18G196000				
	CT-β	1	1	OG0000639	ATCG00500	Ca_23819	Glyma.15G248500						
	BCCP2	2	3	OG0002603	AT5G15530	Ca_10464	Ca_21112	Glyma.13G057400	Glyma.18G265300	Glyma.19G028800			
	BCCP1	1	2	OG0011955	AT5G16390	Ca_06111	Glyma.09G248900	Glyma.18G243500					
	BC	1	2	OG0008875	AT5G35360	Ca_05874	Glyma.05G221100	Glyma.08G027600					
Plastid fatty acid synthesis from pyruvate	MCMT	1	1	OG0009728	AT2G30200	Ca_08991	Glyma.18G057700						
	KASI	1	1	OG0004177	AT5G46290	Ca_05157	Glyma.08G084300						
	KASII	2	1	OG0003934	AT1G74960	Ca_03125	Ca_19284	Glyma.17G047000					
	KASIII	1	2	OG0010869	AT1G62640	Ca_02927	Glyma.09G277400	Glyma.18G211400					
	KAR	1	2	OG0003815	AT1G24360	Ca_11322	Glyma.11G248000	Glyma.18G009200					
	KAR	1	2	OG0000723	AT1G62610	AT1G63380	AT2G17845	AT3G46170	AT3G55290	AT3G55310	Ca_26369	Glyma.02G157200	Glyma.02G159200
	HAD	2	1	OG0003571	AT2G22230	AT5G10160	Ca_00124	Ca_05238	Glyma.15G052500				
	ENR1	2	2	OG0003588	AT2G05990	Ca_11085	Ca_18278	Glyma.08G345900	Glyma.11G101400				

	ACP	3	7	OG0000735	AT1G54580	AT1G54630	Ca_05685	Ca_00539	Ca_06817	Glyma.03G242600	Glyma.19G240100		
							Glyma.05G201300	Glyma.08G008800	Glyma.10G158500	Glyma.13G214600	Glyma.15G098500	Glyma.19G240100	
	DES6	1	1	OG0004928	AT1G43800	Ca_13096	Glyma.14G121400						
	SAD	1	1	OG0015772	AT2G43710	Ca_06396	Glyma.02G138100						
	FATA	1	2	OG0007775	AT3G25110	AT4G13050	Ca_22654	Glyma.08G349200	Glyma.18G167300				
	FATB	2	2	OG0004877	AT1G08510	Ca_06618	Ca_24405	Glyma.05G012300	Glyma.17G120400				
	LACS	2	3	OG0003998	AT1G77590	Ca_12668	Ca_13132	Glyma.06G112900	Glyma.13G079900	Glyma.20G060100			
	ACC2	1	1	OG0006255	AT1G36160	AT1G36180	Ca_16623	Glyma.04G104900					
	GPAT9	2	1	OG0004235	AT5G60620	Ca_17353	Ca_25130	Glyma.09G119200					
	LPAAT3	2	1	OG0008181	AT1G51260	Ca_02646	Ca_05840	Glyma.15G034100					
	PAP	2	3	OG0002509	AT3G09560	Ca_07652	Ca_11988	Glyma.10G046400	Glyma.13G134500	Glyma.19G175600			
	DGAT3	1	2	OG0003135	AT1G48300	Ca_03084	Glyma.13G118300	Glyma.17G041600					
	DGAT1	1	3	OG0003549	AT2G19450	Ca_03178	Glyma.09G065300	Glyma.13G106100	Glyma.17G053300				
	DGAT2	1	1	OG0005170	AT3G51520	Ca_10695	Glyma.01G156000						
	PDAT1	2	3	OG0006779	AT5G13640	Ca_03160	Ca_10036	Glyma.07G036400	Glyma.13G108100	Glyma.16G005800			
	PDAT2	1	1	OG0016093	AT3G44830	Ca_24053	Glyma.12G084000						
	PDAT	1	2	OG0003711	AT4G19860	Ca_06531	Glyma.07G221800	Glyma.20G020800					
TAG synthesis	LPEAT1	1	2	OG0010314	AT1G80950	Ca_00797	Glyma.04G033600	Glyma.06G033800					
	LPEAT2	1	2	OG0007381	AT2G45670	Ca_16422	Glyma.03G019200	Glyma.07G080800					
	LPCAT	1	1	OG0010641	AT1G12640	AT1G63050	Ca_08736	Glyma.17G131500					
	FAD2	2	1	OG0008365	AT3G12120	Ca_07310	Ca_14188	Glyma.03G144500					
	FAD3	2	3	OG0001956	AT3G11170	AT5G05580	Ca_09040	Ca_22205	Glyma.01G120400	Glyma.03G056700	Glyma.18G202600		
	CDS	1	3	OG0010083	AT1G62430	AT4G22340	Ca_00689	Glyma.20G207000	Glyma.02G295000	Glyma.14G018600			
	DAG-CPT	2	2	OG0003870	AT1G13560	AT3G25585	Ca_07133	Ca_14706	Glyma.02G128300	Glyma.12G081900			
	CK	3	3	OG0000668	AT1G71697	AT1G74320	AT4G09760	Ca_04356	Ca_20197	Ca_20392	Glyma.02G078500	Glyma.10G221200	Glyma.20G170300
	LACS	1	2	OG0002940	AT1G64400	AT4G11030	AT4G23850	Ca_15033	Glyma.01G225200	Glyma.11G017900			

	PEAMT	1	1	OG0001888	AT1G48600	AT1G73600	AT3G18000	Ca_10343	Glyma.05G246500					
	PECT1	1	4	OG0009740	AT2G38670	Ca_05880	Glyma.05G221700	Glyma.08G028200	Glyma.09G270100	Glyma.18G220000				
	OBO	1	2	OG0001024	AT2G25890	AT4G25140	AT5G07510	Ca_01856	Glyma.04G077600	Glyma.06G078700				
Oilbody storage proteins	CALO	1	2	OG0008813	AT5G29560	Ca_01255	Glyma.03G249900	Glyma.19G247500						
	STERO	1	2	OG0017523	AT4G10020	Ca_15052	Glyma.01G227900	Glyma.11G015100						
	ApS1	2	1	OG0008665	AT5G48300	Ca_07632	Ca_09767	Glyma.02G304500						
	ApL1	1	1	OG0016301	AT5G19220	Ca_03357	Glyma.07G258500							
	APL	3	4	OG0000724	AT1G27680	AT2G21590	AT4G39210	Ca_04774	Ca_04935	Ca_15382	Glyma.04G011900	Glyma.04G030300	Glyma.06G011700	Glyma.12G042400
	SSI	1	1	OG0012100	AT5G24300	Ca_03985	Glyma.04G235200							
	SSII	1	2	OG0014024	AT3G01180	Ca_10512	Glyma.13G062700	Glyma.19G022900						
	SSIV	1	1	OG0007633	AT4G18240	Ca_05169	Glyma.05G127800							
	SSIII	1	1	OG0006324	AT1G11720	Ca_00636	Glyma.13G204700							
	GBSS	1	2	OG0003139	AT1G32900	Ca_22418	Glyma.07G049900	Glyma.20G218100						
	ISA1	1	1	OG0013057	AT2G39930	Ca_05882	Glyma.08G028400							
Starch metabolism	ISA2	1	2	OG0015999	AT1G03310	Ca_07360	Glyma.03G151200	Glyma.19G153700						
	ISA3	0	1	OG0013267	AT4G09020	Glyma.04G098900	Glyma.06G100600							
	BE	1	1	OG0004502	AT2G36390	AT5G03650	Ca_00773	Glyma.03G192300						
	BE2	1	1	OG0016156	AT3G20440	Ca_20526	Glyma.18G092600							
	PPA	2	1	OG0001001	AT1G01050	AT2G46860	AT3G53620	AT4G01480	Ca_01366	Ca_20986	Glyma.07G048300			
	PHS1	2	2	OG0004146	AT3G29320	Ca_06577	Ca_10467	Glyma.13G057800	Glyma.20G026700					
	PHS2	1	1	OG0006511	AT3G46970	Ca_15595	Glyma.08G334000							
	AMY2	1	1	OG0013846	AT1G76130	Ca_20771	Glyma.14G222600							
	BAM5	1	2	OG0010296	AT4G15210	Ca_22584	Glyma.06G301500	Glyma.12G102900						
	BAM9	1	2	OG0008375	AT3G06330	AT5G18760	Ca_00546	Glyma.13G213600	Glyma.15G099100					

Table S6 | Relative expression levels of candidate genes for the differences of seed oil and starch contents in soybean and chickpea

Protein / gene Abbreviation	Gene	Relative expression levels of genes					Gene	Relative expression levels of genes				
		t1	t2	t3	t4	t5		t1	t2	t3	t4	t5
SUS	*Glyma.09G167000*	0.0219	0.0430	0.0108	0.0051	0.8029	*Ca_13260*	0.0459	0.0337	0.0213	0.0094	0.0065
	Glyma.11G212700	0.0017	0.0008	0.0033	0.0003	2.2917	*Ca_14391*	0.0491	0.0505	0.0360	0.0068	0.0009
	Glyma.14G209900	0.0203	0.0187	0.0229	0.0146	0.0018	*Ca_18036*	0.1802	0.2395	0.1987	0.1393	0.1133
	Glyma.16G217200	0.0188	0.0335	0.0099	0.0053	0.0052						
SUC	*Glyma.04G089200*	0.1010	0.1528	0.0817	0.1457	1.0652	*Ca_21673*	0.2979	0.3211	0.2914	0.2079	4.3535
	Glyma.02G218600	0.2128	0.2546	0.1995	0.8783	1.1980						
	Glyma.18G123400	0.2554	0.3529	0.2529	0.2614	0.0160	*Ca_19491*	0.0000	0.0000	0.0000	0.0000	0.0000
	Glyma.08G298500	0.2391	0.3488	0.2142	0.2797	0.1322						
STP7	*Glyma.15G203300*	0.6924	0.6171	0.8836	0.1746	0.0153	*Ca_06757*	0.1653	0.1656	0.1893	0.1647	0.0106
							Ca_06758	0.0611	0.0438	0.0479	0.0765	0.3399
							Ca_21548	0.3439	0.2247	0.2345	0.1861	0.2151
RBCS	*Glyma.13G046200*	8.9011	7.1007	16.6861	3.3250	0.2639	*Ca_16978*	5.9809	9.5814	13.2279	14.3706	0.0167
	Glyma.19G046600	12.5728	10.4134	23.2627	4.5871	0.8511	*Ca_16979*	0.0000	0.0000	0.0000	0.0000	0.0000
	Glyma.19G046800	15.8413	12.9619	28.4463	5.8925	0.8204						
GRF2	*Glyma.08G265700*	0.0000	0.0000	0.0000	0.0000	0.0000	*Ca_09216*	0.1691	0.1345	0.2031	0.5230	0.8941
	Glyma.17G232600	0.2969	0.7036	0.3964	0.7203	0.7392						
	Glyma.17G232700	0.9163	1.7946	1.1798	1.6812	1.1926						
PGM	*Glyma.05G237000*	1.0878	0.6808	1.9139	1.3334	0.0173	*Ca_10439*	2.3212	2.4680	1.6517	1.5426	0.6001
	Glyma.10G241000	0.1435	0.1919	0.1808	0.9707	0.1184	*Ca_04520*	0.0000	0.0000	0.0000	0.0000	0.0000
FK	*Glyma.02G245800*	0.2577	0.2723	0.2471	0.2310	0.1166	*Ca_14365*	1.1412	0.9024	0.7914	1.2854	0.2117

PGI	*Glyma.19G017200*	0.0000	0.0000	0.0000	0.0000	0.0000	*Ca_10559*	0.5033	0.8067	0.6796	0.6799	0.1359
	Glyma.04G032600	0.3464	0.3388	0.2308	0.7328	0.1193	*Ca_14997*	0.9430	1.3599	1.5494	1.1805	0.2459
	Glyma.06G094300	1.0842	1.0658	0.8217	0.3751	0.0394						
	Glyma.02G212600	0.5516	0.4953	0.4550	0.2235	0.1863						
PFK5	*Glyma.08G280700*	0.1890	0.2064	0.1651	0.1965	0.0263	*Ca_18116*	0.0151	0.0123	0.0116	0.0173	0.0053
PFP-β	*Glyma.07G263800*	1.6477	2.4781	1.4944	1.8204	0.4596	*Ca_03397*	1.2980	1.8367	2.3656	4.7861	2.9207
	Glyma.09G007900	0.8704	0.6965	1.4100	0.5407	0.0341						
PFP-α	*Glyma.07G160500*	1.5840	1.3622	2.7269	1.0604	0.0344	*Ca_14808*	2.0598	2.4855	1.3214	1.2284	0.1038
FBA	*Glyma.04G008300*	0.1524	0.0617	0.2438	0.4701	0.0126	*Ca_05550*	2.3642	3.0528	2.3746	3.1728	6.1013
	Glyma.11G111100	1.6646	1.3115	2.7679	6.9607	0.1175	*Ca_15425*	5.5562	8.2901	12.6101	8.8130	0.1698
	Glyma.11G111400	1.6124	1.2829	2.7450	6.6349	0.1116	*Ca_15426*	0.0000	0.0000	0.0000	0.0000	0.0000
	Glyma.12G037400	1.4510	1.0831	2.8311	9.0918	0.1477						
	Glyma.20G122500	1.7917	1.6380	2.4543	1.3487	0.5533						
GapA	*Glyma.16G044900*	1.5724	1.4281	2.6516	1.4676	0.0224	*Ca_08273*	4.8930	4.4875	3.4395	2.2729	0.0045
	Glyma.19G106800	1.5638	1.4624	2.5395	1.4222	0.1323						
GapCp	*Glyma.03G092700*	1.6365	1.3259	1.5273	0.5043	0.3634	*Ca_22679*	2.4506	2.6163	1.9798	2.2113	1.5512
	Glyma.16G081400	1.3186	1.0821	1.0863	0.4486	0.2232						
PGK	*Glyma.08G165400*	9.9929	6.4238	9.7521	5.3771	2.3025	*Ca_22672*	3.9355	8.0576	11.1440	7.2144	2.5137
	Glyma.08G165500	3.1216	4.5167	4.0990	8.4657	5.0898	*Ca_22673*	7.6079	7.5900	7.2994	5.0303	4.5452
	Glyma.15G262100	9.4088	5.8584	10.2388	5.5605	3.0193						
PK	*Glyma.10G065000*	1.1021	1.0946	1.4807	0.9660	0.3481	*Ca_00753*	0.7977	0.8338	0.7236	1.1187	0.8589
	Glyma.13G149800	1.1936	1.1763	1.5927	0.9471	0.3469	*Ca_08402*	1.3992	1.3525	1.2457	1.2236	0.2461
	Glyma.20G189300	0.1752	0.1927	0.1397	0.8921	0.0267	*Ca_20149*	1.4452	1.3999	1.1329	1.1598	1.9861
PKp-β	*Glyma.01G200200*	0.0451	0.0528	0.0314	0.0087	0.0021	*Ca_02280*	0.2519	0.3397	0.3705	0.9276	0.7554
	Glyma.20G164500	0.5780	0.4749	0.9529	0.3762	0.6394	*Ca_04408*	0.3121	0.3924	0.3763	0.4299	0.3023

							Ca_21051	1.6333	2.1354	1.6940	2.2285	0.0116
PKp-α	*Glyma.20G211200*	1.1949	1.3689	1.5888	0.9844	0.9895	*Ca_12150*	0.3234	0.3912	0.5096	0.8175	1.3342
PEPC4	*Glyma.02G130700*	0.0000	0.0000	0.0000	0.0000	0.0000	*Ca_08446*	2.6844	2.9024	4.0038	2.0877	0.0471
	Glyma.10G205500	0.3119	0.2897	0.4271	0.1787	0.4990	*Ca_16015*	0.0351	0.0732	0.0916	0.0336	0.0417
TPT	*Glyma.13G175100*	4.9951	5.5400	5.4900	1.1317	0.7694	*Ca_06344*	5.5910	5.1230	3.4049	1.7715	0.7928
	Glyma.07G201300	4.3032	4.6826	4.3164	1.3125	1.4973						
PPT2	*Glyma.19G005900*	0.2097	0.2745	0.1240	0.0169	0.0007	*Ca_12580*	0.0110	0.0099	0.0077	0.0034	0.0015
PPT1	*Glyma.06G181600*	1.6244	1.2450	2.6927	0.5713	0.0551	*Ca_07481*	0.7889	1.2033	0.9345	0.3707	0.0475
	Glyma.04G184300	1.5162	1.0686	2.5419	0.5789	0.0456						
GPT	*Glyma.08G312400*	1.7413	1.6130	1.7851	2.9500	0.1919	*Ca_03358*	0.0732	0.3070	1.4380	7.9972	0.0385
	Glyma.13G206700	0.0811	0.0499	0.0893	2.2218	0.3656	*Ca_24458*	2.0302	2.0519	2.4747	2.9129	5.9825
	Glyma.15G105900	0.0739	0.0541	0.0505	3.6352	0.0256						
	Glyma.18G102700	1.6388	1.5148	1.7801	2.9712	0.3037						
G6PDH	*Glyma.19G082300*	0.9656	1.1760	1.6627	0.9154	0.4362	*Ca_19403*	0.2115	0.3289	0.2073	0.2234	0.0673
	Glyma.02G096800	0.4970	0.9021	0.2613	0.2973	0.5874	*Ca_10123*	0.0000	0.0000	0.0000	0.0000	0.0000
	Glyma.07G013800	0.0548	0.1010	0.0589	0.1197	0.0435	*Ca_18820*	0.2085	0.2246	0.2491	0.3265	0.8664
	Glyma.18G284600	1.0670	1.8802	0.7993	0.4529	0.1775						
RPI	*Glyma.15G136200*	0.1485	0.2142	0.1640	0.1446	0.3845	*Ca_08055*	0.7072	0.6737	0.8187	0.3395	0.0424
NAD-ME1	*Glyma.03G102900*	0.2976	0.2041	0.3263	0.2414	0.0285	*Ca_11805*	0.4702	0.3638	0.2394	0.1969	0.1549
NAD-ME2	*Glyma.03G014600*	0.6375	0.6748	0.4907	0.5225	0.4586	*Ca_06238*	0.7841	1.3593	1.2987	0.2781	0.0147
PDH-E1α	*Glyma.03G261000*	0.4522	0.4580	0.5260	0.2422	0.0593	*Ca_01355*	0.9348	1.2418	1.0422	1.6783	2.2462
	Glyma.07G049600	0.9957	0.6806	1.7489	1.1959	0.4752	*Ca_22416*	1.5840	1.7111	1.0626	1.4823	0.8744
	Glyma.16G018300	0.7290	0.5178	1.7155	1.0293	2.8274						
	Glyma.19G260000	0.3757	0.3991	0.4388	0.2318	0.0895						
PDH-E1β	*Glyma.08G096300*	1.1257	0.9016	1.5170	0.5178	0.0757	*Ca_05041*	1.7089	1.8822	1.3860	1.3472	0.4711

CT-α	*Glyma.18G195900*	0.4312	0.6939	0.5631	0.6715	0.0868	*Ca_26217*	0.5806	0.8755	0.4548	0.3210	0.1735
	Glyma.18G195700	0.4508	0.7367	0.7708	1.1923	0.1374						
	Glyma.18G196000	0.0000	0.0000	0.0000	0.0000	0.0000						
CT-β	*Glyma.15G248500*	0.0000	0.0000	0.0000	0.0000	0.0000	*Ca_23819*	0.0023	0.0184	0.0014	0.0085	0.0373
BCCP2	*Glyma.13G057400*	0.1645	0.1231	0.7006	1.6232	0.0899	*Ca_10464*	0.2060	0.2865	0.3890	0.6503	0.0597
	Glyma.18G265300	0.6476	0.8792	1.3569	0.6338	0.0015	*Ca_21112*	0.2593	0.3284	0.1577	0.1598	0.0853
	Glyma.19G028800	0.3572	0.2630	0.8366	3.2502	0.3505						
BCCP1	*Glyma.18G243500*	0.0000	0.0000	0.0000	0.0000	0.0000	*Ca_06111*	0.9711	1.2008	1.0531	1.1008	1.3194
	Glyma.09G248900	1.1282	1.3037	1.1308	0.5377	0.2454						
BC	*Glyma.08G027600*	0.9441	0.7052	1.5100	0.8819	0.4317	*Ca_05874*	0.8502	1.0165	0.9117	1.0624	0.4802
	Glyma.05G221100	0.7964	0.5861	1.3356	0.8765	0.6362						
MCMT	*Glyma.18G057700*	1.4413	1.4018	2.6476	0.9299	0.1322	*Ca_08991*	0.0000	0.0000	0.0000	0.0000	0.0000
KASI	*Glyma.08G084300*	1.6263	1.3543	1.5494	0.5608	0.1397	*Ca_05157*	0.8852	1.2109	1.0369	0.8324	0.0917
KASII	*Glyma.17G047000*	0.9523	1.2098	0.9737	1.0508	0.7556	*Ca_03125*	0.8485	0.6145	0.5389	0.7232	4.5369
							Ca_19284	0.4164	0.4943	0.6953	0.6411	0.1880
KASIII	*Glyma.18G211400*	0.3635	0.3452	0.8528	0.3886	0.3705	*Ca_02927*	0.5100	0.6535	0.4220	0.2442	0.1182
	Glyma.09G277400	0.3368	0.3443	0.8406	0.3938	0.0582						
KAR	*Glyma.18G009200*	1.3059	0.9626	2.7135	0.8902	0.0088	*Ca_11322*	1.0996	1.4189	1.3472	1.2106	0.0971
	Glyma.11G248000	1.2757	0.9407	2.5221	0.7451	0.0061	*Ca_26369*	0.0849	0.1074	0.1335	0.3474	0.0484
KAR	*Glyma.02G157200*	0.2459	0.1661	0.3238	0.0909	0.0447						
	Glyma.02G159200	0.5663	0.4637	0.4757	0.0102	0.0037						
HAD	*Glyma.15G052500*	1.2335	0.5816	5.0131	0.2107	0.2721	*Ca_00124*	2.2418	2.9544	2.8315	1.6802	0.0573
							Ca_05238	1.1391	1.5272	1.3684	1.5375	0.4798
ENR1	*Glyma.08G345900*	0.3549	0.2477	1.1139	0.1570	0.0000	*Ca_11085*	1.4882	1.8182	1.7578	1.8067	0.0590
	Glyma.11G101400	1.7087	1.7103	3.5303	0.9074	0.0244	*Ca_18278*	0.8255	1.3061	1.0826	0.9696	0.0177

ACP	*Glyma.03G242600*	3.7146	4.5175	4.0092	3.2684	1.2575	*Ca_00539*	9.5125	14.2400	13.1216	25.5637	4.3539
	Glyma.05G201300	0.0217	0.0076	0.0343	0.0000	0.0221	*Ca_05685*	0.0000	0.0000	0.0000	0.0000	0.0000
	Glyma.08G008800	0.0354	0.0139	0.0525	0.0000	0.0905	*Ca_06817*	3.6562	6.8038	10.3551	8.7763	0.2790
	Glyma.10G158500	0.9324	1.1881	1.0612	0.9823	2.7742						
	Glyma.13G214600	7.6537	5.5943	16.6226	3.9801	0.5452						
	Glyma.15G098500	1.7595	1.3297	4.5180	1.0896	0.1453						
	Glyma.19G240100	6.6008	7.7357	7.4470	4.9869	1.5003						
DES6	*Glyma.14G121400*	0.6287	0.3350	0.8869	23.9197	0.4028	*Ca_13096*	2.2675	1.8699	2.8094	22.6444	0.1377
SAD	*Glyma.02G138100*	2.0560	2.4916	2.2641	8.0133	1.4360	*Ca_06396*	1.4377	2.2089	3.3962	6.3656	1.0912
FATA	*Glyma.08G349200*	0.4441	0.5729	0.4329	0.7082	0.2487	*Ca_22654*	0.4261	0.4494	0.4497	0.9850	0.1877
	Glyma.18G167300	0.7492	0.9702	0.9649	0.9584	0.2977						
FATB	*Glyma.05G012300*	1.5854	1.3288	1.5537	0.8228	7.9795	*Ca_06618*	2.0939	1.7109	0.8270	0.7181	0.8625
	Glyma.17G120400	1.5719	1.2807	1.5525	0.6755	0.5022	*Ca_24405*	0.5376	0.5787	0.7152	0.8021	0.1037
LACS	*Glyma.06G112900*	0.0550	0.0577	0.2899	0.1648	0.0160	*Ca_12668*	0.3917	0.8404	1.4868	0.8585	0.0385
	Glyma.13G079900	0.1761	0.2635	0.1791	0.1810	0.0405	*Ca_13132*	0.3968	0.4231	0.2843	0.2417	0.1495
	Glyma.20G060100	0.5687	0.6439	0.4435	2.1339	0.8757						
ACC2	*Glyma.04G104900*	0.3239	0.4787	0.5048	0.5090	0.0418	*Ca_16623*	0.3778	0.5741	0.3082	0.1171	0.6084
GPAT9	*Glyma.09G119200*	0.2394	0.3843	0.2227	0.2617	2.9557	*Ca_17353*	0.3601	0.3357	0.3558	0.7381	1.4095
							Ca_25130	0.1874	0.2791	0.3340	0.7698	0.2706
LPAAT3	*Glyma.15G034100*	0.0000	0.0000	0.0000	0.0000	0.0000	*Ca_02646*	0.1034	0.1495	0.1676	0.1167	0.0451
							Ca_05840	0.0330	0.0398	0.0365	0.0195	0.0070
PAP	*Glyma.10G046400*	0.1413	0.2104	0.1231	0.0929	0.2322	*Ca_07652*	1.0298	0.6905	0.8635	0.5319	1.3799
	Glyma.13G134500	0.1413	0.2007	0.1268	0.1166	0.9216	*Ca_11988*	0.1338	0.1094	0.0743	0.0751	0.0100
	Glyma.19G175600	0.0763	0.1055	0.0632	0.0339	0.3840						
DGAT3	*Glyma.17G041600*	1.5832	2.2165	0.9404	0.4225	3.0190	*Ca_03084*	3.7003	2.0056	1.9996	1.6643	5.7903

	Glyma.13G118300	0.5522	0.6385	0.3911	0.5141	2.4953						
DGAT1	*Glyma.13G106100*	0.2327	0.3876	0.1876	1.6635	0.2521	*Ca_03178*	0.0514	0.0393	0.0301	0.3102	0.5518
	Glyma.09G065300	0.0141	0.0216	0.0299	0.3608	0.0340						
	Glyma.17G053300	0.2358	0.3970	0.2002	1.2840	0.2283						
DGAT2	*Glyma.01G156000*	0.0000	0.0000	0.0000	0.0000	0.0000	*Ca_10695*	0.1799	0.2525	0.2626	0.2184	1.0104
PDAT1	*Glyma.07G036400*	0.4316	0.4349	0.2979	0.0047	0.1901	*Ca_03160*	0.0032	0.0272	0.0899	0.3001	0.0557
	Glyma.13G108100	0.0821	0.1059	0.0992	0.5267	4.4401	*Ca_10036*	0.2743	0.2225	0.1505	0.1239	0.1335
	Glyma.16G005800	0.1837	0.1716	0.0737	0.0021	2.1167						
PDAT2	*Glyma.12G084000*	0.0000	0.0000	0.0000	0.0000	0.0000	*Ca_24053*	0.2653	0.2569	0.3568	1.9371	0.1902
PDAT	*Glyma.20G020800*	0.9770	1.2083	0.7361	0.4249	0.5548	*Ca_06531*	1.4260	0.8917	1.0388	1.3374	7.4790
	Glyma.07G221800	1.2567	1.6444	0.9324	0.6298	1.0280	*Ca_00797*	0.7855	0.7715	0.5906	0.7947	1.3681
LPEAT1	*Glyma.06G033800*	0.9159	1.0879	0.8535	0.5161	0.7765	*Ca_16422*	0.2775	0.2679	0.1626	0.1181	0.1187
	Glyma.04G033600	1.0618	1.1746	0.8493	0.6764	0.4311						
LPEAT2	*Glyma.07G080800*	0.2782	0.4657	0.2674	0.4556	0.1437						
	Glyma.03G019200	0.0000	0.0000	0.0000	0.0000	0.0000						
LPCAT	*Glyma.17G131500*	0.7024	0.4558	1.4700	0.7864	0.2851	*Ca_08736*	0.0000	0.0000	0.0000	0.0000	0.0000
FAD2	*Glyma.03G144500*	9.2093	12.9385	7.5429	8.0123	3.3231	*Ca_07310*	15.5102	20.4724	26.6087	34.1468	27.9751
							Ca_14188	0.0806	0.0823	0.0426	0.0053	0.0007
FAD3	*Glyma.01G120400*	0.6401	0.6309	0.4115	0.0812	0.0087	*Ca_09040*	0.4595	0.2603	0.0782	0.2329	0.5085
	Glyma.03G056700	0.6012	0.5637	0.5616	0.1214	0.0172	*Ca_22205*	0.3099	0.2868	0.1538	0.0579	0.4410
	Glyma.18G062000	1.1388	0.6830	1.2734	0.3222	0.3125						
CDS	*Glyma.02G295000*	0.0000	0.0000	0.0000	0.0000	0.0000	*Ca_00689*	0.6147	0.3822	0.3003	0.3989	0.9447
	Glyma.14G018600	0.0052	0.0034	0.0114	0.0004	0.0019						
	Glyma.20G207000	0.7254	0.8228	0.6457	0.5177	0.1590						
DAG-CPT	*Glyma.02G128300*	0.0000	0.0000	0.0000	0.0000	0.0000	*Ca_07133*	0.0000	0.0000	0.0000	0.0000	0.0000

	Glyma.12G081900	1.4990	1.2029	1.5394	0.6508	0.4591	*Ca_14706*	0.0000	0.0000	0.0000	0.0000	0.0000
CK	*Glyma.02G078500*	0.2567	0.4156	0.2227	0.6780	0.3046	*Ca_04356*	0.3786	0.0683	0.3028	0.1603	0.7304
	Glyma.10G221200	0.0465	0.0622	0.0203	0.0733	0.0210	*Ca_20197*	0.0000	0.0000	0.0007	0.0000	0.0000
	Glyma.20G170300	0.0530	0.0907	0.0296	0.1160	0.0213	*Ca_20392*	0.0470	0.0527	0.0461	0.0162	0.1527
LACS	*Glyma.01G225200*	0.7688	1.0638	0.9274	0.3056	0.3948	*Ca_15033*	0.7645	0.6645	0.4410	0.2358	0.1038
	Glyma.11G017900	0.5680	0.9111	0.4789	0.2320	0.4007						
PEAMT	*Glyma.05G246500*	0.6390	0.7031	1.0351	0.2170	2.4426	*Ca_10343*	0.4472	1.2755	1.6972	0.4970	0.0043
PECT1	*Glyma.09G270100*	0.4229	0.4882	0.4476	0.5701	0.4586	*Ca_05880*	1.7437	1.7565	2.0629	0.9163	0.2626
	Glyma.05G221700	2.2582	4.0382	1.9760	1.9427	0.7390						
	Glyma.08G028200	2.0523	3.7902	1.6435	2.4246	1.1323						
	Glyma.18G220000	0.5441	0.5874	0.7020	0.7216	0.9688						
OBO	*Glyma.04G077600*	0.2856	0.0637	1.7708	325.4599	5.9468	*Ca_01856*	0.0004	0.0001	0.0032	0.0130	0.0041
	Glyma.06G078700	0.4180	0.1623	3.1951	411.1953	13.1678						
CALO	*Glyma.03G249900*	0.1598	0.2370	0.2166	0.2780	1.0624	*Ca_01255*	0.0011	0.0038	0.0038	0.0023	0.0006
	Glyma.19G247500	0.3051	0.5104	0.3253	0.8357	3.0853						
STERO	*Glyma.01G227900*	0.1810	0.1483	0.4278	26.9918	14.2931	*Ca_15052*	0.0000	0.0011	0.0523	15.6958	3.2740
	Glyma.11G015100	0.1029	0.0797	0.2164	24.0729	8.6950						
ApS1	*Glyma.02G304500*	0.7943	0.6643	0.9684	1.4491	0.4700	*Ca_07632*	4.2221	3.9503	4.4914	8.6294	0.9478
							Ca_09767	0.8434	0.8204	0.5980	0.4411	0.0014
ApL1	*Glyma.07G258500*	0.0033	0.0013	0.0012	0.0008	0.0024	*Ca_03357*	0.0454	0.0146	0.0112	0.0156	0.0000
APL	*Glyma.04G011900*	1.3233	1.8855	0.4909	1.2501	0.5894	*Ca_04774*	3.1492	5.0492	4.2904	14.1265	1.3275
	Glyma.04G030300	1.1640	1.2914	1.9027	0.5227	0.8182	*Ca_04935*	0.2155	0.2029	0.2857	0.4237	0.0340
	Glyma.06G011700	1.5778	2.2861	0.4829	1.8699	0.3327	*Ca_15382*	0.0879	0.3661	0.3759	0.0268	0.3857
	Glyma.12G042400	0.1773	0.2801	0.1545	0.1280	0.0475						
SSI	*Glyma.04G235200*	0.2995	0.4405	0.0990	0.1037	0.0854	*Ca_03985*	0.7678	0.7318	0.5778	0.3684	0.5280

SSII	*Glyma.13G062700*	0.0000	0.0000	0.0000	0.0000	0.0000	*Ca_10512*	0.5993	0.5539	0.8240	1.9058	0.0732
	Glyma.19G022900	0.8880	1.1549	0.6683	1.5578	0.1465						
SSIV	*Glyma.05G127800*	0.0383	0.0532	0.0601	0.0452	0.0349	*Ca_05169*	0.4850	0.3648	0.4630	0.2897	0.2959
SSIII	*Glyma.13G204700*	0.3210	0.4231	0.2301	0.2613	0.2296	*Ca_00636*	0.4617	0.5346	0.8028	0.8077	0.5264
GBSS	*Glyma.20G218100*	1.1927	1.7313	0.8934	0.9263	0.5160	*Ca_22418*	0.8582	1.1591	1.5141	0.8305	0.0292
	Glyma.07G049900	0.9244	1.2289	0.7262	0.5220	0.2215						
ISA1	*Glyma.08G028400*	0.6280	0.7030	0.6663	0.7008	0.2810	*Ca_05882*	0.3355	0.6471	2.2219	3.1679	0.1945
ISA2	*Glyma.19G153700*	0.2105	0.2781	0.1904	0.1672	0.0083	*Ca_07360*	0.0967	0.1193	0.1529	0.4605	0.0915
	Glyma.03G151200	0.0000	0.0000	0.0000	0.0000	0.0000						
ISA3	*Glyma.06G100600*	0.6033	0.9306	0.6669	0.5907	0.0652						
BE	*Glyma.03G192300*	0.6180	0.6524	0.7546	2.6738	1.1229	*Ca_00773*	1.1457	2.3790	4.3196	5.0830	0.3194
BE2	*Glyma.18G092600*	0.0000	0.0000	0.0000	0.0000	0.0000	*Ca_20526*	0.1319	0.1358	0.0864	0.0616	0.1656
PPA	*Glyma.07G048300*	0.2094	0.1197	0.2415	0.0168	0.0013	*Ca_01366*	0.0086	0.0102	0.0128	0.0019	0.0027
							Ca_20986	0.0053	0.0020	0.0031	0.0075	0.0044
PHS1	*Glyma.13G057800*	1.0160	1.6268	0.6672	3.6200	0.2751	*Ca_06577*	0.6714	0.6255	0.4286	0.4343	0.8242
	Glyma.20G026700	0.0042	0.0061	0.0014	0.0016	0.0652	*Ca_10467*	0.9081	1.1175	1.0071	7.6317	6.2665
PHS2	*Glyma.08G334000*	0.2605	0.4130	0.2336	2.2685	1.0853	*Ca_15595*	2.1642	2.5323	2.3979	3.1610	0.4964
AMY2	*Glyma.14G222600*	0.0298	0.0481	0.0716	0.0900	0.4675	*Ca_20771*	0.2864	0.3604	0.3403	1.2452	0.2830
BAM5	*Glyma.12G102900*	22.2742	18.4414	15.3355	0.5454	0.1834	*Ca_22584*	1.6704	4.8432	3.2701	1.8537	0.0199
	Glyma.06G301500	37.5771	28.1695	31.5602	0.7142	0.5064						
BAM9	*Glyma.13G213600*	0.9622	1.1233	0.7599	0.3957	0.1230	*Ca_00546*	0.2348	0.4770	0.7640	1.0633	0.2005
	Glyma.15G099100	1.0248	1.1425	0.8982	0.6659	0.3828						

Note: The stages t1, t2, t3, t4 and t5 were defined as R3, R4, R5, R7 and R8 in soybean, and S2, S3, S4, S5 and S7 in chickpea, respectively.

Table S7 | Comparison of copy numbers and relative expression levels of starch synthesis genes in soybean and chickpea

Protein		Gene	Copy number of genes			Relative expression levels in soybean			Relative expression levels in Chickpea			Difference
			Soybean	Chickpea	All	Average	Stdev	CV	Average	Stdev	CV	
AGPL	APL2	Os01g44220, Os05g50380	4	2	6	0.83	1.02	1.22	2.57	3.76	1.47	Large
	APL1	Os03g52460	1	1	2	0.00	0.00	1.49	0.02	0.02	0.99	Small
	APL4	Os07g13980	2	1	3	0.02	0.06	2.72				Small
SS	GBSS	Os06g04200, Os07g22930	4	2	6	1.86	1.56	0.84	11.42	27.64	2.42	Large
	SSI	Os06g06560	3	1	4	0.16	0.11	0.65	0.63	0.18	0.29	Small
	SSII	Os02g51070, Os10g30156	2	1	3	0.90	0.43	0.48	0.70	0.62	0.88	Small
	SSIII	Os02g32660	1	1	2	1.14	0.78	0.68	2.20	1.85	0.84	Large
	SSIV	Os01g52250, Os05g45720	2	1	3	0.04	0.01	0.36	0.35	0.09	0.27	Small
SBE	SBEI	Os06g51084	1	1	2	0.35	0.25	0.72	1.38	0.72	0.52	Large
	SBEIII	Os06g26234	1	1	2				0.12	0.04	0.29	Small
ISA	ISAI	Os08g40930	1	1	2	0.57	0.16	0.29	1.02	1.19	1.17	Small
	ISAII	Os05g32710	2	1	3	0.18	0.08	0.47	0.16	0.14	0.86	Small
	ISAIII	Os09g29404	2	0	2	0.63	0.28	0.44				Small
AGPL	APS1	AT5G48300	1	1	2	0.95	0.48	0.50	2.23	2.47	1.11	Large
	APL1	AT5G19220	1	1	2	0.00	0.00	1.28	0.02	0.02	0.99	Small
	APL	AT1G27680	4	4	8	1.05	0.96	0.91	1.78	3.24	1.82	Small
SS	SSI	AT5G24300	1	1	2	0.19	0.14	0.72	0.63	0.18	0.29	Small
	SSII	AT3G01180	2	2	4	0.45	0.55	1.23	0.70	0.62	0.88	Small
	SSIV	AT4G18240	1	1	2	0.04	0.01	0.22	0.35	0.09	0.27	Small
	SSIII	AT1G11720	1	1	2	0.48	0.34	0.70	0.56	0.18	0.33	Small
	GBSS	AT1G32900	2	2	4	1.71	1.42	0.83	0.85	0.62	0.74	Large
ISA	ISAI	AT2G39930	1	1	2	0.57	0.16	0.29	1.02	1.19	1.17	Small
	ISAII	AT1G03310	2	2	4	0.09	0.11	0.19	0.16	0.14	0.86	Small
	ISAIII	AT4G09020	1	1	2	0.63	0.28	0.44				Small
BE	BE3	AT2G36390	1	1	2	1.14	0.78	0.68	2.20	1.85	0.84	Large
	BE2	AT3G20440	1	1	2	0.58			0.12	0.04	0.29	Small
		Sum (average)	45	33	78	0.58	0.42	0.76	1.36	2.04	0.85	

Stdev: Standard deviation; CV: coefficient of variance. AT2G36390 (BE3) in *Arabidopsis thaliana* is homologous to Os02g32660 (SSIII) in rice.

Table S8 | Some miRNAs of regulating candidate genes of lipid and starch metabolisms in soybean and chickpea

miRNA_Acc	Target_Acc	Expectation	miRNA_start	miRNA_end	Target_start	Target_end	miRNA_aligned_fragment	Target_aligned_fragment	Inhibition
Cat-miR156b-3p	Ca_24053	3	1	20	1756	1775	CUCUCUAGACUUCUGUCAUC	GAAGGGAGAGGUCUAGAGAG	Cleavage
Cat-miR157a-3p	Ca_24053	3	1	21	1755	1775	CUCUCUAGACUUCUGUCAUCU	UGAAGGGAGAGGUCUAGAGAG	Cleavage
Cat-miR171h.	Ca_06618	3	1	21	451	471	UUGAGCCGCACCAAUAUCACU	UAUGAAAUUGGUGCGGAUCGA	Cleavage
Cat-miR5565g-3p	Ca_06618	3	1	20	943	962	ACAUGUGGAUUGAUAUUAAU	CUUGAUGUCAAUCAGCAUGU	Cleavage
gma-miR10199	Glyma.16G217200	2.5	1	21	1567	1587	AGCAAUGUUGAGCUUGGGCCU	GAUCCAAAGUUCAACAUUGCU	Cleavage
gma-miR10199	Glyma.09G167000	2.5	1	21	1567	1587	AGCAAUGUUGAGCUUGGGCCU	GAUCCAAAGUUCAACAUUGCU	Cleavage
gma-miR2111a	Glyma.13G204700	2.5	1	21	907	927	GUCCUUGGGAUGCAGAUUACG	AAUAAGUUGCAUCUCAAGGGC	Cleavage
gma-miR2111d	Glyma.13G204700	2.5	1	21	907	927	GUCCUUGGGAUGCAGAUUACG	AAUAAGUUGCAUCUCAAGGGC	Cleavage
gma-miR5784	Glyma.02G304500	2.5	1	21	337	357	AAUUAGCUAAUGGUUAGCUAA	GGAGCUAACUAUAGGCUAAUU	Cleavage
gma-miR10413a	Glyma.20G018000	3	1	22	60	81	UUUUGGUAGAGAACGAAACCCU	AAAGUUUCCUUCUUUACCAGGG	Cleavage
gma-miR10413b	Glyma.20G018000	3	1	22	60	81	UUUUGGUAGAGAACGAAACCCU	AAAGUUUCCUUCUUUACCAGGG	Cleavage
gma-miR4347	Glyma.02G304500	3	1	24	913	936	AAGCUUCUUACGGAUCAAGUUGAU	UUAGACCUGCUCCGUGAGAAGUUU	Cleavage
Cat-miR159b-3p.1	Ca_25013	0	1	21	956	976	AUUGGAGUGAAGGGAGCUCCA	UGGAGCUCCCUUCACUCCAAU	Cleavage
Cat-miR159b-3p.1	Ca_21739	0	1	21	929	949	AUUGGAGUGAAGGGAGCUCCA	UGGAGCUCCCUUCACUCCAAU	Cleavage
Cat-miR159b	Ca_25013	1.5	1	20	957	976	AUUGGAUUGAAGGGAGCUCC	GGAGCUCCCUUCACUCCAAU	Cleavage
Cat-miR159b	Ca_21739	1.5	1	20	930	949	AUUGGAUUGAAGGGAGCUCC	GGAGCUCCCUUCACUCCAAU	Cleavage
Cat-miR159c.1	Ca_25013	1.5	1	20	956	975	UUGGAUUGAAGGGAGCUCCC	UGGAGCUCCCUUCACUCCAA	Cleavage
Cat-miR159c.1	Ca_21739	1.5	1	20	929	948	UUGGAUUGAAGGGAGCUCCC	UGGAGCUCCCUUCACUCCAA	Cleavage
Cat-miR159d.2	Ca_25013	1.5	1	20	957	976	AUUGGAUUGAAGGGAGCUCC	GGAGCUCCCUUCACUCCAAU	Cleavage
Cat-miR159d.2	Ca_21739	1.5	1	20	930	949	AUUGGAUUGAAGGGAGCUCC	GGAGCUCCCUUCACUCCAAU	Cleavage
Cat-miR159a.2	Ca_25013	2	1	21	956	976	GUUGGAUUGAAGGGAGCUCUC	UGGAGCUCCCUUCACUCCAAU	Cleavage

Cat-miR159a.2	Ca_21739	2	1	21	929	949	GUUGGAUUGAAGGGAGCUCUC	UGGAGCUCCCUUCACUCCAAU	Cleavage
Cat-miR319a-3p	Ca_25013	1.5	1	21	955	975	UUGGACUGAAGGGAGCUCCCU	UUGGAGCUCCCUUCACUCCAA	Cleavage
Cat-miR319a-3p	Ca_21739	1.5	1	21	928	948	UUGGACUGAAGGGAGCUCCCU	CUGGAGCUCCCUUCACUCCAA	Cleavage
Cat-miR319c.2	Ca_25013	1.5	1	20	956	975	UUGGACUGAAGGGAGCUCCC	UGGAGCUCCCUUCACUCCAA	Cleavage
Cat-miR319c.2	Ca_21739	1.5	1	20	929	948	UUGGACUGAAGGGAGCUCCC	UGGAGCUCCCUUCACUCCAA	Cleavage
Cat-miR319g.2	Ca_25013	1.5	1	20	956	975	UUGGACUGAAGGGAGCUCCC	UGGAGCUCCCUUCACUCCAA	Cleavage
Cat-miR319g.2	Ca_21739	1.5	1	20	929	948	UUGGACUGAAGGGAGCUCCC	UGGAGCUCCCUUCACUCCAA	Cleavage
Cat-miR319	Ca_25013	2.5	1	21	956	976	UUUGGACUGAAGGGAGCUCCC	UGGAGCUCCCUUCACUCCAAU	Cleavage
Cat-miR319	Ca_21739	2.5	1	21	929	949	UUUGGACUGAAGGGAGCUCCC	UGGAGCUCCCUUCACUCCAAU	Cleavage
Cat-miR319b	Ca_25013	2.5	1	21	955	975	UUGGACUGAAGGGUGCUCCCU	UUGGAGCUCCCUUCACUCCAA	Cleavage
Cat-miR319b	Ca_21739	2.5	1	21	928	948	UUGGACUGAAGGGUGCUCCCU	CUGGAGCUCCCUUCACUCCAA	Cleavage
Cat-miR319a-3p	Ca_10228	3	1	21	425	445	UUGGACUGAAGGGAGCUCCCU	CGGGAGCUUCUGUUAGUCCAA	Translation
Cat-miR319a.2	Ca_10228	3	1	20	426	445	UUGGACUGAAGGGAGCUCCC	GGGAGCUUCUGUUAGUCCAA	Translation
Cat-miR319c.2	Ca_10228	3	1	20	426	445	UUGGACUGAAGGGAGCUCCC	GGGAGCUUCUGUUAGUCCAA	Translation
Cat-miR319g.2	Ca_10228	3	1	20	426	445	UUGGACUGAAGGGAGCUCCC	GGGAGCUUCUGUUAGUCCAA	Translation
gma-miR159b-3p	Glyma.20G047600	1	1	21	953	973	AUUGGAGUGAAGGGAGCUCCA	UGGAGCUCCCUUCACUCCAAU	Cleavage
gma-miR159c	Glyma.20G047600	1	1	21	953	973	AUUGGAGUGAAGGGAGCUCCG	UGGAGCUCCCUUCACUCCAAU	Cleavage
gma-miR159f-3p	Glyma.20G047600	1	1	21	953	973	AUUGGAGUGAAGGGAGCUCCA	UGGAGCUCCCUUCACUCCAAU	Cleavage
gma-miR159b-3p	Glyma.04G125700	1	1	21	932	952	AUUGGAGUGAAGGGAGCUCCA	CAGAGCUCCCUUCACUCCAAA	Cleavage
gma-miR159c	Glyma.04G125700	1	1	21	932	952	AUUGGAGUGAAGGGAGCUCCG	CAGAGCUCCCUUCACUCCAAA	Cleavage
gma-miR159f-3p	Glyma.04G125700	1	1	21	932	952	AUUGGAGUGAAGGGAGCUCCA	CAGAGCUCCCUUCACUCCAAA	Cleavage
gma-miR159a-3p	Glyma.04G125700	1.5	1	21	932	952	UUUGGAUUGAAGGGAGCUCUA	CAGAGCUCCCUUCACUCCAAA	Cleavage
gma-miR159e-3p	Glyma.04G125700	1.5	1	21	932	952	UUUGGAUUGAAGGGAGCUCUA	CAGAGCUCCCUUCACUCCAAA	Cleavage
gma-miR319a	Glyma.20G047600	1.5	1	20	953	972	UUGGACUGAAGGGAGCUCCC	UGGAGCUCCCUUCACUCCAA	Cleavage

gma-miR319b	Glyma.20G047600	1.5	1	20	953	972	UUGGACUGAAGGGAGCUCCC	UGGAGCUCCCUUCACUCCAA	Cleavage
gma-miR319c	Glyma.20G047600	1.5	1	20	953	972	UUGGACUGAAGGGAGCUCCU	UGGAGCUCCCUUCACUCCAA	Cleavage
gma-miR319e	Glyma.20G047600	1.5	1	20	953	972	UUGGACUGAAGGGAGCUCCC	UGGAGCUCCCUUCACUCCAA	Cleavage
gma-miR319g	Glyma.20G047600	1.5	1	22	951	972	UUGGACUGAAGGGAGCUCCUUC	GUUGGAGCUCCCUUCACUCCAA	Cleavage
gma-miR319a	Glyma.04G125700	2.5	1	20	932	951	UUGGACUGAAGGGAGCUCCC	CAGAGCUCCCUUCACUCCAA	Cleavage
gma-miR319b	Glyma.04G125700	2.5	1	20	932	951	UUGGACUGAAGGGAGCUCCC	CAGAGCUCCCUUCACUCCAA	Cleavage
gma-miR319c	Glyma.04G125700	2.5	1	20	932	951	UUGGACUGAAGGGAGCUCCU	CAGAGCUCCCUUCACUCCAA	Cleavage
gma-miR319e	Glyma.04G125700	2.5	1	20	932	951	UUGGACUGAAGGGAGCUCCC	CAGAGCUCCCUUCACUCCAA	Cleavage
gma-miR319g	Glyma.04G125700	2.5	1	22	930	951	UUGGACUGAAGGGAGCUCCUUC	GUCAGAGCUCCCUUCACUCCAA	Cleavage
gma-miR319h	Glyma.04G125700	2.5	1	21	931	951	UUGGACUGAAGGGAGCUCCCU	UCAGAGCUCCCUUCACUCCAA	Cleavage
gma-miR319j	Glyma.04G125700	2.5	1	21	931	951	UUGGACUGAAGGGAGCUCCCU	UCAGAGCUCCCUUCACUCCAA	Cleavage
gma-miR319a	Glyma.19G021900	3	1	20	978	997	UUGGACUGAAGGGAGCUCCC	UGGAUUUCCCUUCAGUUUGA	Cleavage
gma-miR319b	Glyma.19G021900	3	1	20	978	997	UUGGACUGAAGGGAGCUCCC	UGGAUUUCCCUUCAGUUUGA	Cleavage
gma-miR319c	Glyma.19G021900	3	1	20	978	997	UUGGACUGAAGGGAGCUCCU	UGGAUUUCCCUUCAGUUUGA	Cleavage
gma-miR319e	Glyma.19G021900	3	1	20	978	997	UUGGACUGAAGGGAGCUCCC	UGGAUUUCCCUUCAGUUUGA	Cleavage
gma-miR319g	Glyma.19G021900	3	1	22	976	997	UUGGACUGAAGGGAGCUCCUUC	ACUGGAUUUCCCUUCAGUUUGA	Cleavage
gma-miR319h	Glyma.19G021900	3	1	21	977	997	UUGGACUGAAGGGAGCUCCCU	CUGGAUUUCCCUUCAGUUUGA	Cleavage
gma-miR319j	Glyma.19G021900	3	1	21	977	997	UUGGACUGAAGGGAGCUCCCU	CUGGAUUUCCCUUCAGUUUGA	Cleavage
gma-miR319k	Glyma.19G021900	3	1	21	977	997	UUGGACUGAAGGGAGCUCCCU	CUGGAUUUCCCUUCAGUUUGA	Cleavage
gma-miR319l	Glyma.19G021900	3	1	22	976	997	UUGGACUGAAGGGAGCUCCUUC	ACUGGAUUUCCCUUCAGUUUGA	Cleavage
gma-miR319m	Glyma.19G021900	3	1	21	977	997	UUGGACUGAAGGGAGCUCCCU	CUGGAUUUCCCUUCAGUUUGA	Cleavage
Cat-miR166k	Ca_07056	2	1	20	289	308	UCGGACCAGGCUUCAUUCAU	GUGGAUGAAGUUUGGUCUGA	Cleavage
Cat-miR166e.2	Ca_07056	2.5	1	21	289	309	UUCGGACCAGGCUUCAUUCCC	GUGGAUGAAGUUUGGUCUGAG	Cleavage
Cat-miR166f	Ca_07056	2.5	1	21	290	310	UUUCGGACCAGGCUUCAUUCC	UGGAUGAAGUUUGGUCUGAGA	Cleavage

Cat-miR166u	Ca_07056	2.5	1	20	291	310	UUUCGGACCAGGCUUCAUUU	GGAUGAAGUUUGGUCUGAGA	Cleavage
Cat-miR166b.2	Ca_07056	3	1	22	287	308	UCGGACCAGGCUUCAUUCCCUC	CUGUGGAUGAAGUUUGGUCUGA	Cleavage
Cat-miR166d.2	Ca_07056	3	1	21	288	308	UCGGACCAGGCUUCAUUCCAU	UGUGGAUGAAGUUUGGUCUGA	Cleavage
Cat-miR166j-3p	Ca_07056	3	1	21	288	308	UCGGACCAGGCUUCAUUCCCG	UGUGGAUGAAGUUUGGUCUGA	Cleavage
Cat-miR166m.2	Ca_07056	3	1	20	288	307	CGGACCAGGCUUCUUUCACC	UGUGGAUGAAGUUUGGUCUG	Cleavage
Cat-miR1507a	Ca_10228	2.5	1	22	292	313	UUUCAUUCCAUACAUCGUCUAA	GUGGAUGAUGUAUGGAGAGAAA	Cleavage
Cat-miR1507b	Ca_10228	2.5	1	21	293	313	UUUCAUUCCAUACAUCGUCUA	UGGAUGAUGUAUGGAGAGAAA	Cleavage
Cat-miR166b.2	Ca_06348	3	1	22	211	232	UCGGACCAGGCUUCAUUCCCUC	GCUGGAGUGAAGUUUGAUCCGA	Cleavage
Cat-miR166d.2	Ca_06348	3	1	21	212	232	UCGGACCAGGCUUCAUUCCAU	CUGGAGUGAAGUUUGAUCCGA	Cleavage
Cat-miR166e.2	Ca_06348	3	1	21	213	233	UUCGGACCAGGCUUCAUUCCC	UGGAGUGAAGUUUGAUCCGAA	Cleavage
Cat-miR166j-3p	Ca_06348	3	1	21	212	232	UCGGACCAGGCUUCAUUCCCG	CUGGAGUGAAGUUUGAUCCGA	Cleavage
Cat-miR164a-5p	Ca_13537	1	1	21	602	622	UGGAGAAGCAGGGCACGUGCA	AGCAAGUGCCCUGCUUCUCCA	Cleavage
Cat-miR164a.1	Ca_13537	1	1	21	602	622	UGGAGAAGCAGGGCACUUUUU	AGCAAGUGCCCUGCUUCUCCA	Cleavage
Cat-miR164a.2	Ca_13537	1	1	21	602	622	UGGAGAAGCAGGGCACGUGAA	AGCAAGUGCCCUGCUUCUCCA	Cleavage
Cat-miR164c-5p	Ca_13537	1	1	21	602	622	UGGAGAAGCAGGGCACGUGCG	AGCAAGUGCCCUGCUUCUCCA	Cleavage
Cat-miR164c.2	Ca_13537	1	1	21	602	622	UGGAGAAGCAGGGCACGUGCU	AGCAAGUGCCCUGCUUCUCCA	Cleavage
Cat-miR164d	Ca_13537	1	1	21	602	622	UGGAGAAGCAGGGCACAUGCU	AGCAAGUGCCCUGCUUCUCCA	Cleavage
Cat-miR164h-5p	Ca_13537	1	1	21	602	622	UGGAGAAGCAGGGCACGUGUA	AGCAAGUGCCCUGCUUCUCCA	Cleavage
Cat-miR164	Ca_13537	1.5	1	21	602	622	UGGAGAAGCAGGGUACGUGCA	AGCAAGUGCCCUGCUUCUCCA	Cleavage
Cat-miR164b	Ca_13537	2	1	20	603	622	UGGAGAAGCAGGGCACGUUC	GCAAGUGCCCUGCUUCUCCA	Cleavage
Cat-miR164e	Ca_13537	2	1	21	602	622	UGGAGAAGCAGGGCACGUUAC	AGCAAGUGCCCUGCUUCUCCA	Cleavage
Cat-miR164c.1	Ca_13537	2.5	1	21	602	622	UGGAGAAGCAGGACACGUGCA	AGCAAGUGCCCUGCUUCUCCA	Cleavage
Cat-miR164f	Ca_13537	2.5	1	21	602	622	UGGAGAAGAAGGGCACGUGCA	AGCAAGUGCCCUGCUUCUCCA	Cleavage
Cat-miR167h-5p	Ca_19433	2.5	1	21	458	478	UGAAGCUGCCAACAUGAUCUG	CGGAACAAGUUGGUAGCUUCA	Cleavage

Cat-miR167-5p	Ca_19433	3	1	21	458	478	UGAAGCUGCCAGCAUGAUCUU	CGGAACAAGUUGGUAGCUUCA	Cleavage
Cat-miR167a	Ca_19433	3	1	22	457	478	UGAAGCUGCCAGCAUGAUCUUA	CCGGAACAAGUUGGUAGCUUCA	Cleavage
Cat-miR167a-5p	Ca_19433	3	1	21	458	478	UGAAGCUGCCAGCAUGAUCUA	CGGAACAAGUUGGUAGCUUCA	Cleavage
Cat-miR167c.1	Ca_19433	3	1	22	457	478	UGAAGCUGCCAGCAUGAUCUUA	CCGGAACAAGUUGGUAGCUUCA	Cleavage
Cat-miR167c.2	Ca_19433	3	1	21	458	478	UGAAGCUGCCAGCAUGAUCUC	CGGAACAAGUUGGUAGCUUCA	Cleavage
Cat-miR167d-5p	Ca_19433	3	1	22	457	478	UGAAGCUGCCAGCAUGAUCUGG	CCGGAACAAGUUGGUAGCUUCA	Cleavage
Cat-miR167d.1	Ca_19433	3	1	21	458	478	UGAAGCUGCCAGCAUGAUCUG	CGGAACAAGUUGGUAGCUUCA	Cleavage
Cat-miR167d.2	Ca_19433	3	1	20	459	478	UGAAGCUGCCAGCAUGAUCU	GGAACAAGUUGGUAGCUUCA	Cleavage
gma-miR482a-3p	Glyma.07G213100	1.5	1	24	143	166	UCUUCCCAAUUCCGCCCAUUCCUA	GCACAAUGGGUGGGAUUGGGAAGG	Cleavage
gma-miR482c-3p	Glyma.07G213100	2	1	21	144	164	UUCCCAAUUCCGCCCAUUCCU	CACAAUGGGUGGGAUUGGGAA	Cleavage
gma-miR482a-3p	Glyma.02G131700	1.5	1	24	149	172	UCUUCCCAAUUCCGCCCAUUCCUA	GCACCAUGGGUGGGAUUGGGAAGG	Cleavage
gma-miR482c-3p	Glyma.02G131700	3	1	21	150	170	UUCCCAAUUCCGCCCAUUCCU	CACCAUGGGUGGGAUUGGGAA	Cleavage
gma-miR164a	Glyma.08G173400	1	1	21	602	622	UGGAGAAGCAGGGCACGUGCA	AGCAAGUGCCCUGCUUCUCCA	Cleavage
gma-miR164b	Glyma.08G173400	1	1	20	603	622	UGGAGAAGCAGGGCACGUGC	GCAAGUGCCCUGCUUCUCCA	Cleavage
gma-miR164c	Glyma.08G173400	1	1	20	603	622	UGGAGAAGCAGGGCACGUGC	GCAAGUGCCCUGCUUCUCCA	Cleavage
gma-miR164d	Glyma.08G173400	1	1	20	603	622	UGGAGAAGCAGGGCACGUGC	GCAAGUGCCCUGCUUCUCCA	Cleavage
gma-miR164e	Glyma.08G173400	1	1	21	602	622	UGGAGAAGCAGGGCACGUGCA	AGCAAGUGCCCUGCUUCUCCA	Cleavage
gma-miR164f	Glyma.08G173400	1	1	21	602	622	UGGAGAAGCAGGGCACGUGCA	AGCAAGUGCCCUGCUUCUCCA	Cleavage
gma-miR164g	Glyma.08G173400	1	1	21	602	622	UGGAGAAGCAGGGCACGUGCA	AGCAAGUGCCCUGCUUCUCCA	Cleavage
gma-miR164h	Glyma.08G173400	1	1	21	602	622	UGGAGAAGCAGGGCACGUGCA	AGCAAGUGCCCUGCUUCUCCA	Cleavage
gma-miR164i	Glyma.08G173400	1	1	21	602	622	UGGAGAAGCAGGGCACGUGCA	AGCAAGUGCCCUGCUUCUCCA	Cleavage
gma-miR164j	Glyma.08G173400	1	1	21	602	622	UGGAGAAGCAGGGCACGUGCA	AGCAAGUGCCCUGCUUCUCCA	Cleavage
gma-miR164k	Glyma.08G173400	1	1	21	602	622	UGGAGAAGCAGGGCACGUGCA	AGCAAGUGCCCUGCUUCUCCA	Cleavage
gma-miR164a	Glyma.15G254000	1	1	21	602	622	UGGAGAAGCAGGGCACGUGCA	AGCAAGUGCCCUGCUUCUCCA	Cleavage

gma-miR164b	Glyma.15G254000	1	1	20	603	622	UGGAGAAGCAGGGCACGUGC	GCAAGUGCCCUGCUUCUCCA	Cleavage
gma-miR164c	Glyma.15G254000	1	1	20	603	622	UGGAGAAGCAGGGCACGUGC	GCAAGUGCCCUGCUUCUCCA	Cleavage
gma-miR164d	Glyma.15G254000	1	1	20	603	622	UGGAGAAGCAGGGCACGUGC	GCAAGUGCCCUGCUUCUCCA	Cleavage
gma-miR164e	Glyma.15G254000	1	1	21	602	622	UGGAGAAGCAGGGCACGUGCA	AGCAAGUGCCCUGCUUCUCCA	Cleavage
gma-miR164f	Glyma.15G254000	1	1	21	602	622	UGGAGAAGCAGGGCACGUGCA	AGCAAGUGCCCUGCUUCUCCA	Cleavage
gma-miR164g	Glyma.15G254000	1	1	21	602	622	UGGAGAAGCAGGGCACGUGCA	AGCAAGUGCCCUGCUUCUCCA	Cleavage
gma-miR164h	Glyma.15G254000	1	1	21	602	622	UGGAGAAGCAGGGCACGUGCA	AGCAAGUGCCCUGCUUCUCCA	Cleavage
gma-miR164i	Glyma.15G254000	1	1	21	602	622	UGGAGAAGCAGGGCACGUGCA	AGCAAGUGCCCUGCUUCUCCA	Cleavage
gma-miR164j	Glyma.15G254000	1	1	21	602	622	UGGAGAAGCAGGGCACGUGCA	AGCAAGUGCCCUGCUUCUCCA	Cleavage
gma-miR164k	Glyma.15G254000	1	1	21	602	622	UGGAGAAGCAGGGCACGUGCA	AGCAAGUGCCCUGCUUCUCCA	Cleavage
gma-miR164a	Glyma.12G226500	1	1	21	701	721	UGGAGAAGCAGGGCACGUGCA	AGCACGUGUCCUGUUUCUCCA	Cleavage
gma-miR164b	Glyma.12G226500	1	1	20	702	721	UGGAGAAGCAGGGCACGUGC	GCACGUGUCCUGUUUCUCCA	Cleavage
gma-miR164c	Glyma.12G226500	1	1	20	702	721	UGGAGAAGCAGGGCACGUGC	GCACGUGUCCUGUUUCUCCA	Cleavage
gma-miR164d	Glyma.12G226500	1	1	20	702	721	UGGAGAAGCAGGGCACGUGC	GCACGUGUCCUGUUUCUCCA	Cleavage
gma-miR164e	Glyma.12G226500	1	1	21	701	721	UGGAGAAGCAGGGCACGUGCA	AGCACGUGUCCUGUUUCUCCA	Cleavage
gma-miR164f	Glyma.12G226500	1	1	21	701	721	UGGAGAAGCAGGGCACGUGCA	AGCACGUGUCCUGUUUCUCCA	Cleavage
gma-miR164g	Glyma.12G226500	1	1	21	701	721	UGGAGAAGCAGGGCACGUGCA	AGCACGUGUCCUGUUUCUCCA	Cleavage
gma-miR164h	Glyma.12G226500	1	1	21	701	721	UGGAGAAGCAGGGCACGUGCA	AGCACGUGUCCUGUUUCUCCA	Cleavage
gma-miR164i	Glyma.12G226500	1	1	21	701	721	UGGAGAAGCAGGGCACGUGCA	AGCACGUGUCCUGUUUCUCCA	Cleavage
gma-miR164j	Glyma.12G226500	1	1	21	701	721	UGGAGAAGCAGGGCACGUGCA	AGCACGUGUCCUGUUUCUCCA	Cleavage
gma-miR164k	Glyma.12G226500	1	1	21	701	721	UGGAGAAGCAGGGCACGUGCA	AGCACGUGUCCUGUUUCUCCA	Cleavage
gma-miR10186a	Glyma.19G002900	3	1	22	1116	1137	UUGGGAAUUAAAAUGUAAACUU	UAAUUUACAAUUUGGUUCUCAA	Cleavage
gma-miR10186b	Glyma.19G002900	3	1	24	1116	1139	UUUUGGGAAUUAAAAUGUAAACUU	UAAUUUACAAUUUGGUUCUCAAGA	Cleavage
gma-miR10186c	Glyma.19G002900	3	1	24	1116	1139	UUUUGGGAAUUAAAAUGUAAACUU	UAAUUUACAAUUUGGUUCUCAAGA	Cleavage

gma-miR10186d	Glyma.19G002900	3	1	24	1116	1139	UUUUGGGAAUUAAAAUGUAAACUU	UAAUUUACAAUUUGGUUCUCAAGA	Cleavage
gma-miR10186e	Glyma.19G002900	3	1	22	1116	1137	UUGGGAAUUAAAAUGUAAACUU	UAAUUUACAAUUUGGUUCUCAA	Cleavage
gma-miR10186f	Glyma.19G002900	3	1	24	1116	1139	UUUUGGGAAUUAAAAUGUAAACUU	UAAUUUACAAUUUGGUUCUCAAGA	Cleavage
gma-miR10186g	Glyma.19G002900	3	1	24	1116	1139	UUUUGGGAAUUAAAAUGUAAACUU	UAAUUUACAAUUUGGUUCUCAAGA	Cleavage
gma-miR10186h	Glyma.19G002900	3	1	24	1116	1139	UUUUGGGAAUUAAAAUGUAAACUU	UAAUUUACAAUUUGGUUCUCAAGA	Cleavage
gma-miR10186i	Glyma.19G002900	3	1	24	1116	1139	UUUUGGGAAUUAAAAUGUAAACUU	UAAUUUACAAUUUGGUUCUCAAGA	Cleavage
gma-miR10186j	Glyma.19G002900	3	1	24	1116	1139	UUUUGGGAAUUAAAAUGUAAACUU	UAAUUUACAAUUUGGUUCUCAAGA	Cleavage
gma-miR10186k	Glyma.19G002900	3	1	24	1116	1139	UUUUGGGAAUUAAAAUGUAAACUU	UAAUUUACAAUUUGGUUCUCAAGA	Cleavage
gma-miR10186l	Glyma.19G002900	3	1	24	1116	1139	UUUUGGGAAUUAAAAUGUAAACUU	UAAUUUACAAUUUGGUUCUCAAGA	Cleavage
gma-miR1514a-5p	Glyma.07G048000	0.5	1	21	712	732	UUCAUUUUUAAAAUAGGCAUU	AAUGCCUAUUUUAGAAAUGAA	Cleavage
gma-miR1514b-5p	Glyma.07G048000	1.5	1	21	712	732	UUCAUUUUUAAAAUAGACAUU	AAUGCCUAUUUUAGAAAUGAA	Cleavage
gma-miR1514a-5p	Glyma.07G048100	0.5	1	21	715	735	UUCAUUUUUAAAAUAGGCAUU	AAUGCCUAUUUUAGAAAUGAA	Cleavage
gma-miR1514b-5p	Glyma.07G048100	1.5	1	21	715	735	UUCAUUUUUAAAAUAGACAUU	AAUGCCUAUUUUAGAAAUGAA	Cleavage
gma-miR390a-5p	Glyma.04G175800	3	1	21	55	75	AAGCUCAGGAGGGAUAGCGCC	GCCUCUAUCAUUCCUGAGCUU	Cleavage
gma-miR390f	Glyma.04G175800	3	1	21	55	75	AAGCUCAGGAGGGAUAGCGCC	GCCUCUAUCAUUCCUGAGCUU	Cleavage
gma-miR390g	Glyma.04G175800	3	1	21	55	75	AAGCUCAGGAGGGAUAGCGCC	GCCUCUAUCAUUCCUGAGCUU	Cleavage
gma-miR390a-5p	Glyma.08G360200	3	1	21	127	147	AAGCUCAGGAGGGAUAGCGCC	GCCUCUAUCAUUCCUGAGCUU	Cleavage
gma-miR390f	Glyma.08G360200	3	1	21	127	147	AAGCUCAGGAGGGAUAGCGCC	GCCUCUAUCAUUCCUGAGCUU	Cleavage
gma-miR390g	Glyma.08G360200	3	1	21	127	147	AAGCUCAGGAGGGAUAGCGCC	GCCUCUAUCAUUCCUGAGCUU	Cleavage

Table S9. miRNAs, transcription factors (TFs), and their target genes associated with starch biosynthesis in soybean and chickpea

TF	Target_Acc	Co-expressed gene	Protein/gene	miRNA_Acc
bZIP	Ca_07056	Ca_04774	APL	
	Ca_07056	Ca_07360	ISA2	Cat-miR166k, Cat-miR166b.2, Cat-miR166e.2, Cat-miR166d.2, Cat-miR166f, Cat-miR166j-3p, Cat-miR166u, Cat-miR166m.2
	Ca_07056	Ca_10512	SSII	
	Ca_10228	Ca_22418	GBSS	Cat-miR319a-3p, Cat-miR319c.2, Cat-miR319a.2, Cat-miR319g.2
	Glyma.07G213100	Glyma.06G005400	SSI	
	Glyma.07G213100	Glyma.13G325200	SSI	gma-miR482a-3p, gma-miR482c-3p
	Glyma.02G131700	Glyma.13G325200	SSI	
NAC36	Ca_06348	Ca_04774	APL	
	Ca_06348	Ca_07632	ApS1	
	Ca_06348	Ca_00773	BE	Cat-miR166b.2, Cat-miR166d.2 Cat-miR166e.2, Cat-miR166j-3p
	Ca_06348	Ca_07360	ISA2	
	Ca_06348	Ca_10512	SSII	
	Ca_13537	Ca_03985	SSI	
	Ca_13537	Ca_00636	SSIII	Cat-miR164a.1, Cat-miR164a.2, Cat-miR164a-5p, Cat-miR164b, Cat-miR164, Cat-miR164c.1, Cat-miR164c.2, Cat-miR164c-5p, Cat-miR164d, Cat-miR164e, Cat-miR164f, Cat-miR164h-5p
	Ca_13537	Ca_05169	SSIV	
	Glyma.08G173400	Glyma.04G235200	SSI	
	Glyma.15G254000	Glyma.04G235200	SSI	gma-miR164a, gma-miR164b, gma-miR164e, gma-miR164f, gma-miR164g, gma-miR164h, gma-miR164i, gma-miR164j, gma-miR164k
	Glyma.12G226500	Glyma.13G204700	SSIII	

	Glyma.19G002900	Glyma.04G235200	SSI	gma-miR10186a, gma-miR10186b, gma-miR10186c. gma-miR10186d, gma-miR10186e, gma-miR10186f, gma-miR10186g, gma-miR10186h, gma-miR10186i, gma-miR10186k
	Glyma.07G048000	Glyma.04G235200	SSI	gma-miR1514a-5p, gma-miR1514b-5p
	Glyma.07G048100	Glyma.04G235200	SSI	
	Glyma.04G175800	Glyma.04G235200	SSI	gma-miR390a-5p, gma-miR390f, gma-miR390g
	Glyma.08G360200	Glyma.04G235200	SSI	
	Glyma.19G021900	Glyma.13G204700	SSIII	gma-miR319a, gma-miR319b, gma-miR319c, gma-miR319e, gma-miR319g, gma-miR319h, gma-miR319j, ma-miR319k, gma-miR319l, gma-miR319m
DOF	Ca_19433	Ca_22418	GBSS	Cat-miR167h-5p, t-miR167-5p, Cat-miR167a, Cat-miR167a-5p, Cat-miR167c.1, Cat-miR167c.2, Cat-miR167d-5p, Cat-miR167d.1, Cat-miR167d.2
